# Versatile Para‐Substituted Pyridine Lanthanide Coordination Complexes Allow Late Stage Tailoring of Complex Function

**DOI:** 10.1002/chem.202103243

**Published:** 2021-11-16

**Authors:** Matthieu Starck, Jack D. Fradgley, Davide F. De Rosa, Andrei S. Batsanov, Maria Papa, Michael J. Taylor, Janet E. Lovett, Jacob C. Lutter, Matthew J. Allen, David Parker

**Affiliations:** ^1^ Department of Chemistry Durham University South Road Durham DH1 3LE UK; ^2^ SUPA School of Physics and Astronomy and BSRC University of St Andrews North Haugh St Andrews KY16 9SS UK; ^3^ Department of Chemistry Wayne State University 5101 Cass Avenue Detroit MI 48202 USA

**Keywords:** europium, EPR, luminescence, gadolinium

## Abstract

A series of cationic and neutral *p*−Br and *p*−NO_2_ pyridine substituted Eu(III) and Gd(III) coordination complexes serve as versatile synthetic intermediates. Nucleophilic aromatic substitution occurs readily at the *para* position under mild conditions, allowing C−N and C−C bond forming reactions to take place, permitting the introduction of azide, amino and alkynyl substituents. For Eu(III) complexes, this approach allows late stage tuning of absorption and emission spectral properties, exemplified by the lowering of the energy of an LMCT transition accompanied by a reduction in the Eu−N_py_ bond length. Additionally, these complexes provide direct access to the corresponding Eu(II) analogues. With the Gd(III) series, the nature of the *p*‐substituent does not significantly change the EPR properties (linewidth, relaxation times), as required for their development as EPR spin probes that can be readily conjugated to biomolecules under mild conditions.

## Graphical abstract

**A series of cationic** and neutral *p*‐bromo and *p*‐nitropyridine substituted Eu(III) and Gd(III) coordination complexes serve as versatile synthetic intermediates. This approach allows the late stage tuning of absorption and emission spectroscopic properties for Eu(III) complexes, and provides easy access to the corresponding Eu(II) analogues, aiding the development of Gd(III) EPR spin probes that can be readily conjugated to biomolecules, under mild conditions.

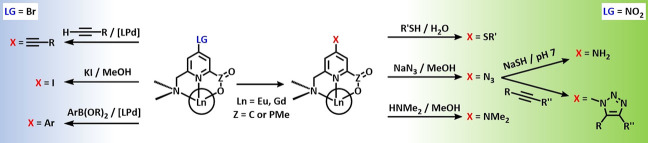

## Introduction

The preparation of metal coordination complexes is normally undertaken using a convergent process, introducing the metal ion in the final step. Such a strategy is often adopted, as it permits ligand synthesis in relatively non‐polar media, aided by classical purification techniques such as normal phase chromatography. An alternative divergent approach uses a robust metal coordination complex that can itself serve as a common intermediate for the synthesis of a family of structurally related complexes. When designed with appropriate functional groups that can be modified or elaborated under relatively mild conditions, this divergent approach greatly increases the efficiency of the overall synthetic process.

Such an approach has been adopted in some areas of modern coordination chemistry, particularly with kinetically inert metal complexes. Williams, for example, has explored the reactivity of several Ir(III) complexes, notably with aryl bromide functionality, using a variety of Suzuki coupling reactions. In this way, their conjugation length could be varied tuning the HOMO‐LUMO energy gap, or multimeric systems could be created using Pd catalysed reactions.[^1^, ^2^, ^3^] In lanthanide coordination chemistry, the modular synthesis of hetero‐metallic complexes has been devised with kinetically stable complexes of heptadentate and octadentate ligands, via different strategies that include the use of a series of Pd(0), Ugi or Cu(I) catalysed ‘click’ reactions.[^4^, ^5^, ^6^, ^7^, ^8^, ^9^, ^10^, ^11^]

**Scheme 1:**
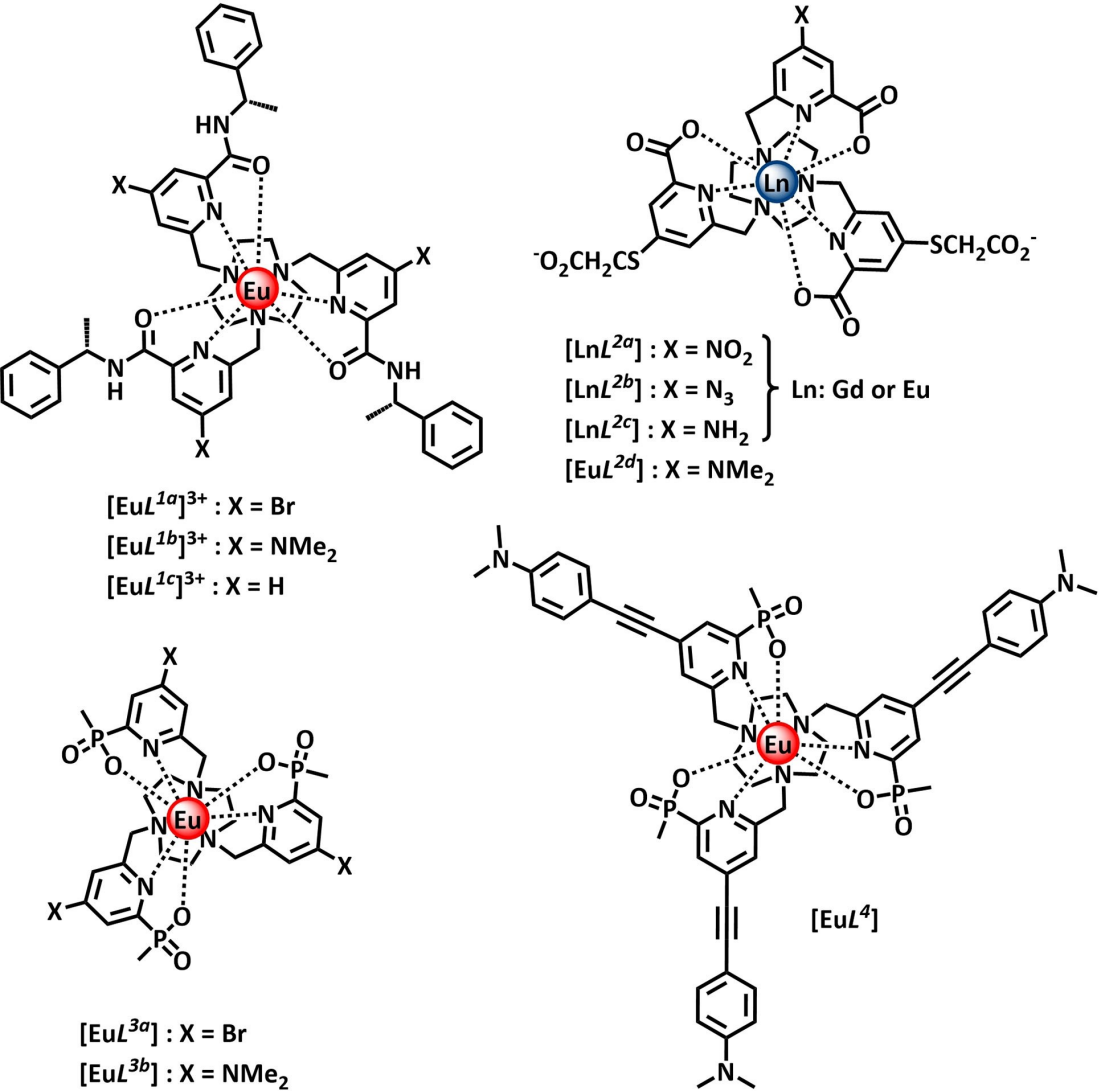
Lanthanide coordination complexes examined here.

**Scheme 2:**
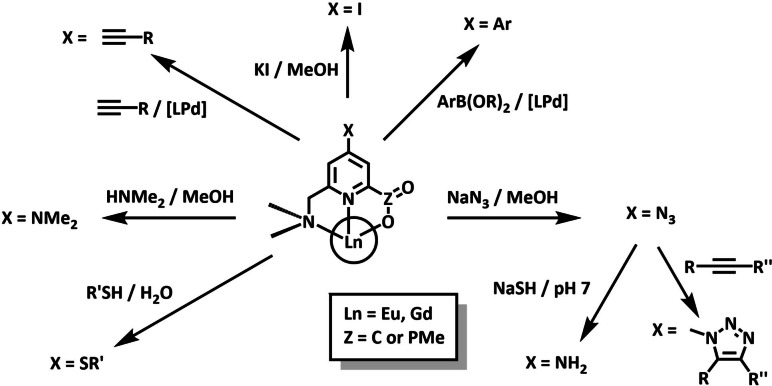
Synthetic transformations of *p*‐substituted pyridyl complexes.

In this work, we exemplify the synthetic versatility of the *p*−Br and *p*−NO_2_ pyridine lanthanide coordination complexes, [Eu ***L*** ^***1a‐c***^], [Ln ***L*** ^***2a‐2d***^] and [Eu ***L*** ^***3a***^] (Scheme 1), where displacement of the *para* substituent by a range of charged and uncharged nucleophiles is possible under mild conditions, (Scheme 2). This late stage functionalisation strategy avoids the need for separate ligand syntheses (exemplified here, for comparison, in the rather laborious stepwise synthesis of the non‐emissive complex, [Eu ***L*** ^***4***^]) (given in the Supporting Information), and allows the desired property of the complex to be imparted, or tuned, at the end of the synthetic pathway by a one‐step structural transformation of the complex. In earlier work we have shown how this approach permits C−C bond formation, by activation of the *p*−Br pyridyl bond under palladium catalysis.^[11]^

With Eu(III) complexes, we show here how this approach opens up various possibilities: it enables the tailoring of excited state energies involving the chromophore and modifies the metal ion excited state lifetime for luminescent probe development;[^12^, ^13^, ^14^] it permits the introduction of functionality that allows subsequent one‐step conjugation processes;[^15^, ^16^, ^17^, ^18^] it allows access to simple derivatives that may serve as activity based probes,[^19^, ^20^, ^21^] for example for H_2_S detection;[^22^, ^23^, ^24^] it provides precursors that are one reduction step away from the corresponding Eu(II) complexes that might function as magnetic or luminescent probes or photocatalysts.[^25^, ^26^, ^27^] For each of these aspects, the *p*−NO_2_ pyridyl complexes are particularly attractive, and their potential has been highlighted earlier, notably in their reactivity towards Cys‐thiol residues for selective peptide or protein conjugation.[^15^, ^16^, ^17^, ^28^, ^29^] The Eu emission intensity (and for that matter, luminescence from any Ln(III) ion) is very weak following excitation into a proximate chromophore, as intramolecular electron transfer to the nitro‐aryl moiety efficiently quenches the singlet excited state of the sensitising chromophore, severely limiting the overall efficiency of metal based emission. On the other hand, the more electron donating thio, azido, halo, and amino substituents on aryl rings are not so prone to this particular quenching pathway, and emission from the lanthanide ion switches on following reaction; such lanthanide complexes are intrinsically much more brightly luminescent. Following reduction of Eu(III) to Eu(II), 4*f*–5*d* absorbance and emission bands are observed as well as characteristic EPR signals of Eu(II).

With the Gd(III) examples, (S=7/2), owing to the absence of orbital angular momentum on the ion, ligand field effects at the metal centre are considered to be less relevant. Furthermore, the Gd^3+^ ion shows a reduced propensity to accept electron density (i.e. to act as a charge sink), so that LMCT transitions are of much higher energy, particularly when compared to Eu^3+^ (and Yb^3+^) complexes. We have therefore taken advantage of the late stage functionalisation strategy to investigate the zero field splitting and relaxation behaviour of a small set of Gd(III) complexes via powder line‐shape and relaxation measurements. Such parameters are of particular importance in defining their applicability as EPR spin labels.[^28^, ^29^, ^30^]

## Results and Discussion

### Complex synthesis and synthetic versatility

**Scheme 3:**
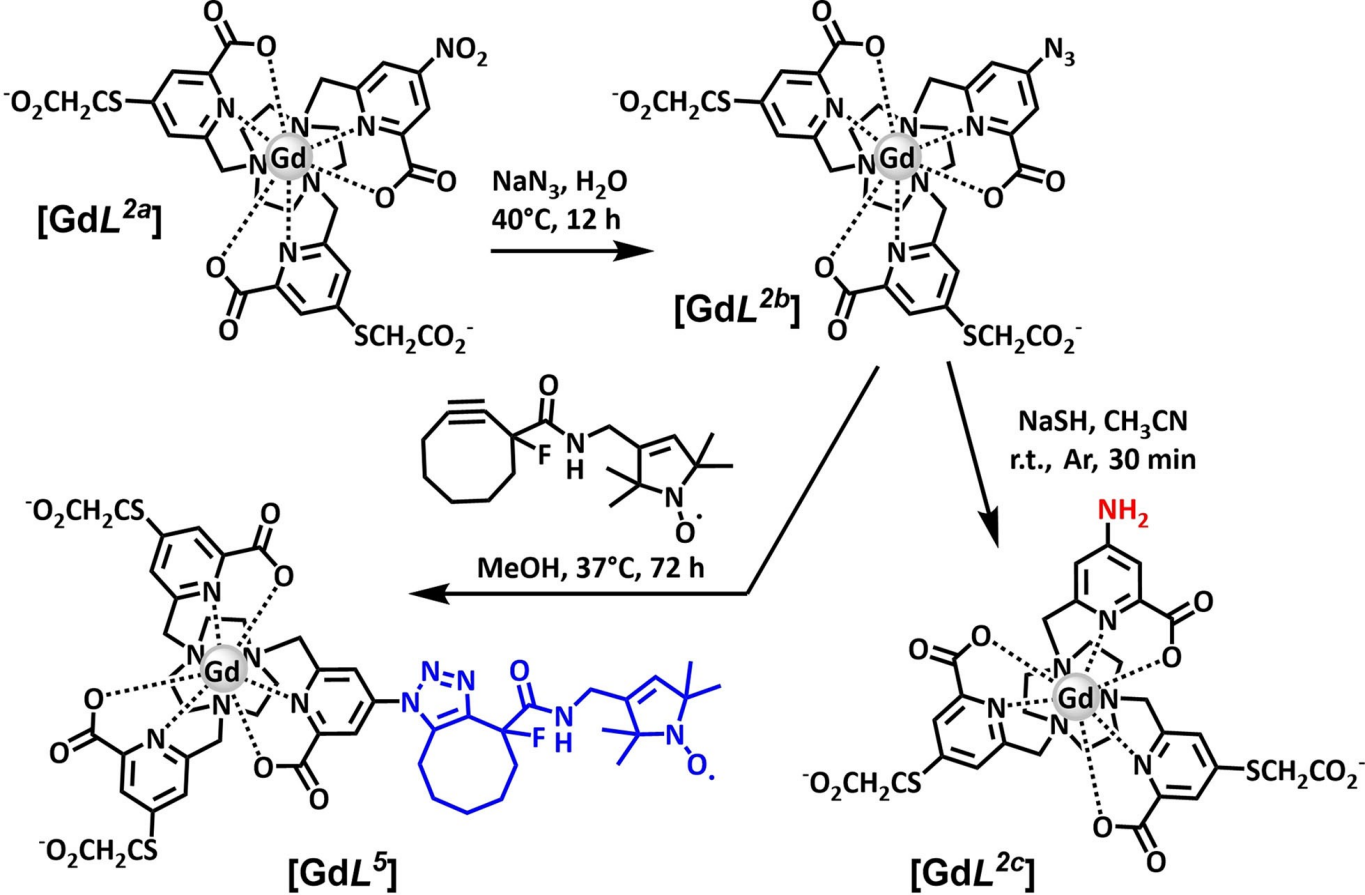
Transformations of Gd(III) complexes.

A series of simple functional group transformations was examined, (Scheme 1), to highlight the reactivity and synthetic versatility of the 4‐substitued pyridyl series of kinetically stable lanthanide complexes. The stepwise synthesis of the ligand ***L*** ^***1a***^ was undertaken following standard methods reported for the parent complex, ***L*** ^***1c***^, and details are given in the SI.[^31^, ^32^] The synthesis of the ligand ***L*** ^***2a***^ (and its Gd complex) and ***L*** ^***3a***^ have been reported earlier.[^11^, ^28^] Displacement of the Br or NO_2_ group by a dimethylamino group occurred cleanly at room temperature in methanol with [Eu ***L*** ^***1a***^]^3+^, [Eu ***L*** ^***2a***^], and [Eu ***L*** ^***3a***^] to give [Eu ***L*** ^***1b***^]^3+^, [Eu ***L*** ^***2d***^] and [Eu ***L*** ^***3b***^], respectively. Previously, we have shown that the *p*−Br substituent in [Eu ***L*** ^***3a***^] can be directly displaced by azide in DMF.^[11]^ It was found that this reaction can also be undertaken in water, and proceeded cleanly within 12 h at 40 °C when applied to the synthesis of the Eu and Gd complexes, [Ln ***L*** ^***2b***^], (Scheme 3 for the Gd series).

The aryl azides reacted with H_2_S in aqueous solution or with NaSH in MeCN in under 30 minutes to afford the 4‐amino‐complexes, [Ln ***L*** ^***2c***^], consistent with their established utility as activity based sensors for H_2_S in solution.^[22]^ The azide group in [Gd ***L*** ^***2b***^] also can undergo dipolar cycloaddition reactions, and using strained alkynes, the reaction requires no copper catalysis. Accordingly, reaction with the cyclooctyne derivative of a stable nitroxide radical^[33]^ was undertaken in MeOH at 37 °C. Reaction progress was monitored by LCMS, revealing clean transformation to a single compound, the triazole, [Gd ***L*** ^***5***^], (Scheme 3). The constitution of the triazole is assumed to be that shown, based on literature precedent and the minimisation of steric strain in the transition state leading to product formation.

Reductions of Eu(III) complexes [Eu ***L*** ^***1a‐c***^]^3+^, [Eu ***L*** ^***3b***^], and [Eu ***L*** ^***4***^] were performed using zinc dust in MeOH under an inert atmosphere. Of the reduced species, [Eu ***L*** ^***1a***^] and [Eu ***L*** ^***1c***^] show 4*f*–5*d* absorption and 5*d*–4*f* emission bands consistent with the presence of Eu(II), but all five reduced complexes have EPR signals consistent with the presence of Eu(II).^[34]^ In the case of [Eu ***L*** ^***3b***^] and [Eu ***L*** ^***4***^] the neutral charge of these complexes might suppress the zinc to europium electron transfer process compared to cationic complexes such as [Eu ***L*** ^***1a‐c***^]^3+^. For [Eu ***L*** ^***1b***^]^3+^, the electron‐donating NMe_2_ group places more charge on the N_py_ atoms, stabilizing the Eu(III) species relative to the Eu(II) species.

### Absorption and emission spectroscopic behaviour

**Figure 1:**
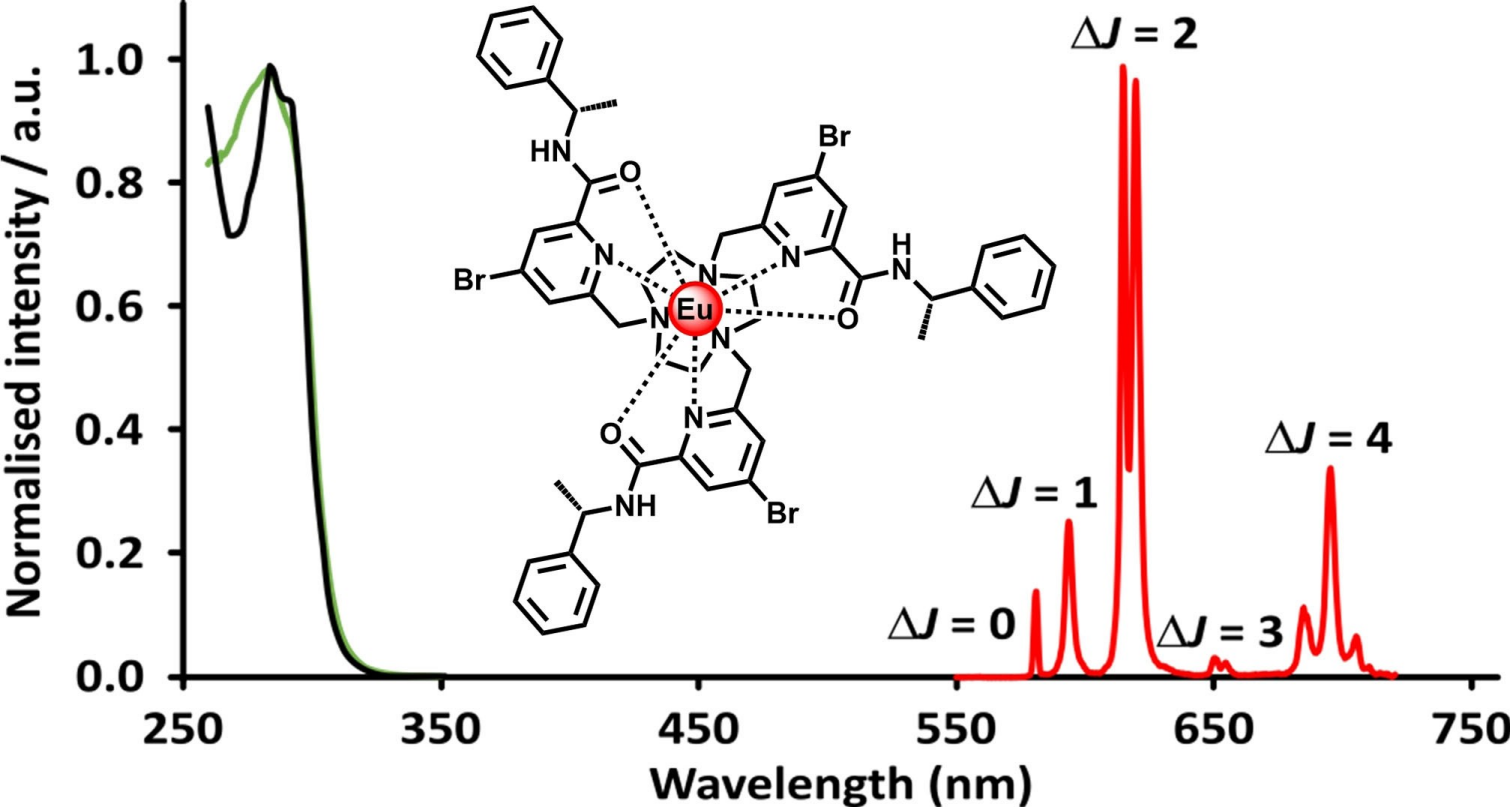
The absorption (*black*), excitation (*green, λ*_em_ 620 nm) and emission (*red*, *λ*_ex_ 280 nm) spectra for [Eu ***L*** ^**1a**^](CF_3_SO_3_)_3_ (295 K, MeOH).

The absorption and excitation spectra recorded for [Eu ***L*** ^***1a***^]^3+^ are very similar, with an absorption band at 290 nm, typical of the π‐π* transition of the pyridyl chromophore (Figure 1). The emission spectrum of [Eu ***L*** ^***1a***^]^3+^ (Figure 1) is similar to those observed for related Eu(III) complexes[^31^, ^32^, ^35^] based on triazacyclononane, with the hypersensitive Δ*J*=2 transition around 620 nm, (^5^D_0_→^7^F_2_), being the most intense. In this case, two major components were observed and three main transitions were evident in the Δ*J*=4 manifold, as found with structurally related Eu(III) complexes in C_3_ symmetry.^[36]^

**Figure 2:**
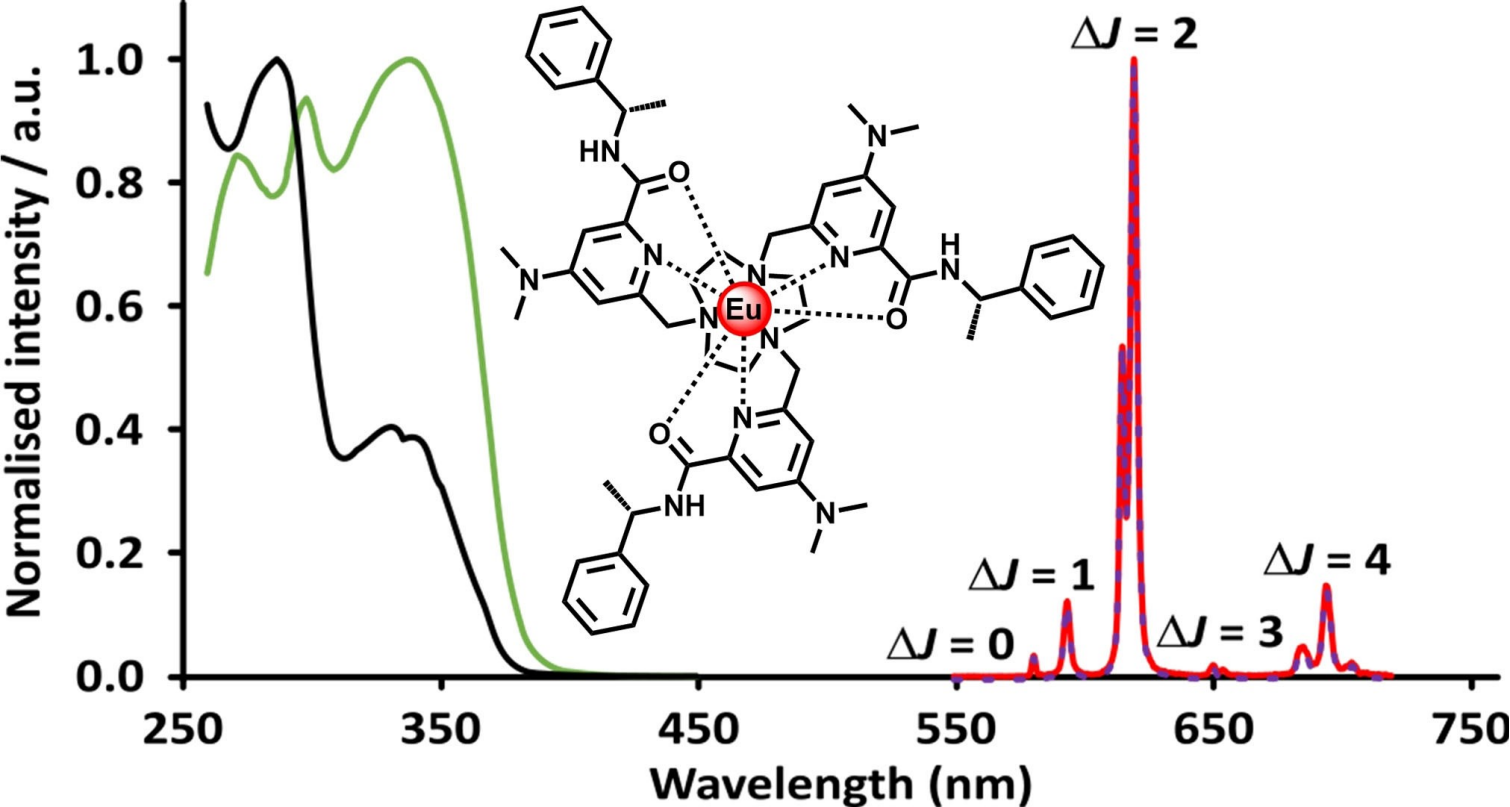
The absorption (*black*), excitation (*green, λ*_em_ 620 nm) and emission spectra (red, *λ*_ex_ 290 nm; *purple dots, λ*_ex_ 340 nm) for [Eu ***L*** ^***1b***^]^3+^ (MeOH, 295 K).

For [Eu ***L*** ^***1b***^]^3+^ a strong absorption band (Figure 2) also occurred at 290 nm, but an additional broad band was apparent centred at 345 nm corresponding to a well‐defined and relatively narrow internal ligand to metal charge transfer (LMCT) transition. No change in Eu emission lifetime in MeOH was found for these two complexes. However, the form of the Eu emission spectrum was slightly different: the Δ*J*=2 band for [Eu ***L*** ^***1a***^]^3+^ was centred around 619 nm and was the most intense transition observed, but the lower energy component doubled in relative intensity.

The Δ*J*=2 transition manifold in Eu(III) complexes is electric dipole allowed, and is often described as ‘hypersensitive’.^[36]^ Here, the lower energy component was much more intense for [Eu ***L*** ^***1b***^]^3+^ than with the *p*−Br analogue. Such a change can be interpreted in terms of the much stronger dipole moment present in the former case, where electron density from the π‐conjugated N lone pair is donated to the charge sink of the pyridine‐coordinated tripositive Eu cation. This effect, which causes the LMCT band to appear at longer wavelength, perturbs the polarisability of the pyridyl moiety, modifying the multipolar electrostatic interaction, including that between the quadrupole moment on the lanthanide ion and the induced dipole moment on the pyridyl group.

**Figure 3:**
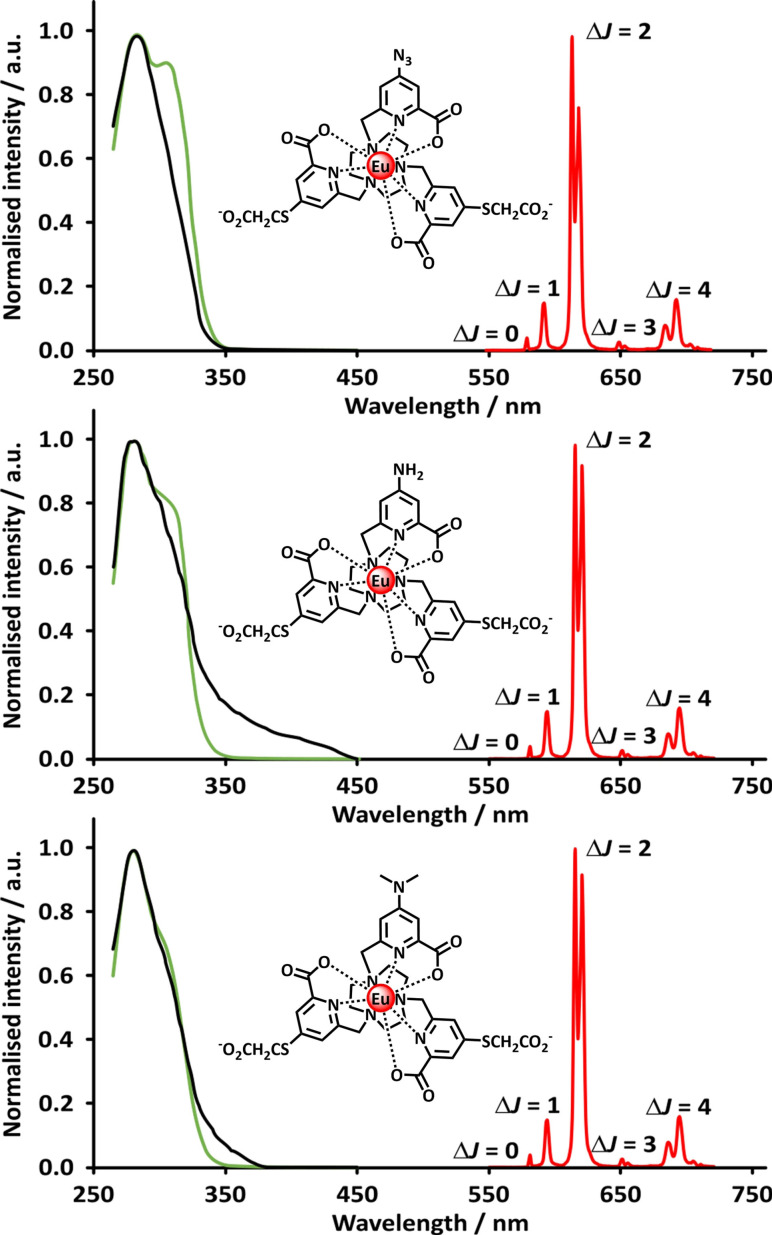
(Top) The absorption (*black*), excitation (*green*, *λ*_em_ 615 nm), and emission (*red*, *λ*_exc_ 280 nm) spectra for [Eu ***L*** ^***2b***^] (H_2_O, 295 K); (centre) corresponding spectra for [Eu ***L*** ^***2c***^] and (bottom) [Eu ***L*** ^***2d***^] under the same conditions. The *p*‐nitro complex, [Eu ***L*** ^***2a***^], shows virtually no sensitised Eu luminescence, under these conditions, owing to quenching of the intermediate ligand S_1_ state by electron transfer (see Supporting Information).

An LMCT absorption band is also apparent for the 4‐amino‐pyridyl complex, [Eu ***L*** ^***2c***^] and to a lesser extent with the *N,N*‐dimethylamino analogue, [Eu ***L*** ^***2d***^], when comparing absorption and excitation spectral behaviour with the 4‐nitro and 4‐azido precursors, [Eu ***L*** ^***2a,b***^] (Figure 3).

In these two cases, compared to [Eu ***L*** ^***1b***^]^3+^, the lowest energy LMCT absorption band is both less intense and broader, extending to 450 nm for [Eu ***L*** ^***2c***^]. The greater breadth of the LMCT absorption band for the *p*‐NH_2_ substituted complex compared to the NMe_2_ analogue accords with classical steric inhibition to lone pair conjugation in the latter case, caused by unfavourable interactions between the methyl groups and the 3,5 pyridine ring hydrogen atoms that inhibit the N lone pair from being at 90° to the plane of the pyridyl ring.

The similarity of the three excitation spectra strongly suggests that the thio‐substituted chromophores are functioning as the sensitising antenna in each case. Europium emission spectra are nearly identical, and the lifetimes of [Eu ***L*** ^***2a***^], [Eu ***L*** ^***2b***^], [Eu ***L*** ^***2c***^] and [Eu ***L*** ^***2d***^] in water were 1.27, 0.90, 0.83 and 0.74 ms, respectively, indicating some quenching of the Eu excited state for the latter three complexes. Such behaviour is consistent with potential energy surface crossing of the Eu ^5^D_0_ excited state with the ligand to metal charge transfer state (LMCT) reducing the Eu emission lifetime slightly.[^12^, ^37^]

In contrast to the Eu series of complexes, (Figures S34–S41 and S43, S45) the absorption spectra of [Gd ***L*** ^***2a‐c***^] showed no evidence of a low energy charge transfer band (Figure S42). This behaviour strongly suggests that the Eu(III) ion serves as an essential charge sink, lowering the energy of the ligand to metal charge transfer state. The effect is most well‐defined when the Eu ion bears net positive charge, i.e. with [Eu ***L*** ^***1b***^]^3+^. Accordingly, evidence was sought to establish if there were any significant differences in the structures of the cationic 4‐substituted series of complexes.

### Structural Analysis of [EuL^1a‐c^]^3+^

The X‐ray crystal structures of the cationic Eu(III) complexes of ***L*** ^***1a***^ and ***L*** ^***1b***^ were determined at 120 K, following crystallisation from aqueous methanol. Crystallographic analyses of the lanthanide(III) triflate salts of the parent complex, [Ln ***L*** ^***1c***^]^3+^ (Ln=Eu to Yb) had been carried out earlier, revealing an isomorphous series.[^31^, ^32^] For [Eu ***L*** ^***1a***^]^3+^, crystals of the trifluoromethanesulfonate salt were also examined. The crystals of the complex of ***L*** ^***1b***^ were obtained from the reaction mixture, following displacement of the ring bromide in [Eu ***L*** ^***1a***^]^3+^. The isolated crystals were found to be the mono‐triflate dibromide mixed anion salt, and the presence of different anions in the lattice was not expected to perturb the coordination geometry of the 9‐coordinate cation significantly.

**Figure 4:**
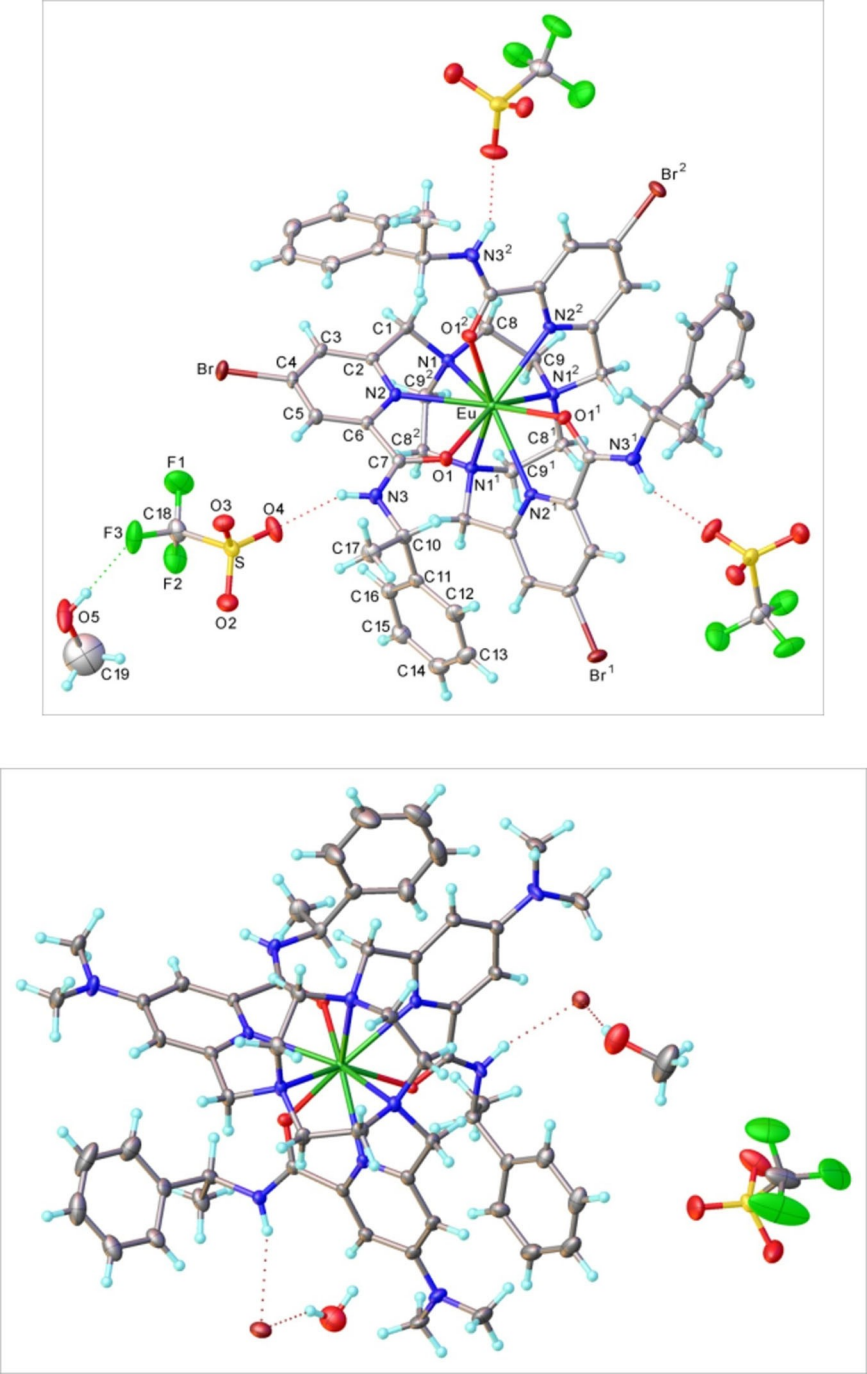
Views of the structures of the trivalent cations of S‐Δ‐(*λλλ*)‐[Eu ***L*** ^***1a***^] (top) and [Eu ***L*** ^***1b***^] (bottom) (120 K); (Deposition Number(s) 2096811 and 2096812 contain(s) the supplementary crystallographic data for this paper. These data are provided free of charge by the joint Cambridge Crystallographic Data Centre and Fachinformationszentrum Karlsruhe http://www.ccdc.cam.ac.uk/structures Access Structures service.

No variation was found in the mean Eu−O or Eu−N_ring_ bond distances across the three complexes considered here, (Table 1, Figure 4), but the Eu−N_py_ bond length was shorter by 0.05 Å for the dimethylamino‐substituted complex, [Eu ***L*** ^***1b***^]^3+^. Such a bond length decrease accords with a stronger ion‐dipole interaction, associated with the larger dipole moment of the pyridyl groups in [Eu ***L*** ^***1b***^]^3+^, and is consistent with the presence of the low energy sensitising LMCT band observed for [Eu ***L*** ^***1b***^]^3+^ in its absorption and excitation spectra.

**Table 1:** Bond lengths (±0.015 Å) to the europium ion for [Eu ***L*** ^***1a‐c***^]^3+^.^[a,b]^

Bond	[Eu*L* ^*1a*^]^3+^	[Eu*L* ^*1b*^]^3+^	[Eu*L* ^*1c*^]^3+[a]^
Eu−O	2.412(3)	2.410(3) 2.403(3) 2.430(3)	2.405(3)
Eu−N_py_	2.569(3)	2.514(3) 2.521(3) 2.516(4)	2.567(3)
Eu−N_ring_	2.633(3)	2.627(3) 2.634(3) 2.645(4)	2.628(3)

[a] data from references 28 and 29. [b] complexes of ***L*** ^***1a***^ and ***L*** ^***1c***^ crystallised as the simple triflate salt in R3, and for ***L*** ^***1b***^ (isolated as the mono‐triflate‐dibromide salt) in P21; see Supporting Information for details of the structural refinement: CCDC 2096811 and 2096812; see 965909 for [Eu ***L*** ^***1c***^]^3+^.

### Spectroscopic properties of Eu(II) complexes

**Figure 5:**
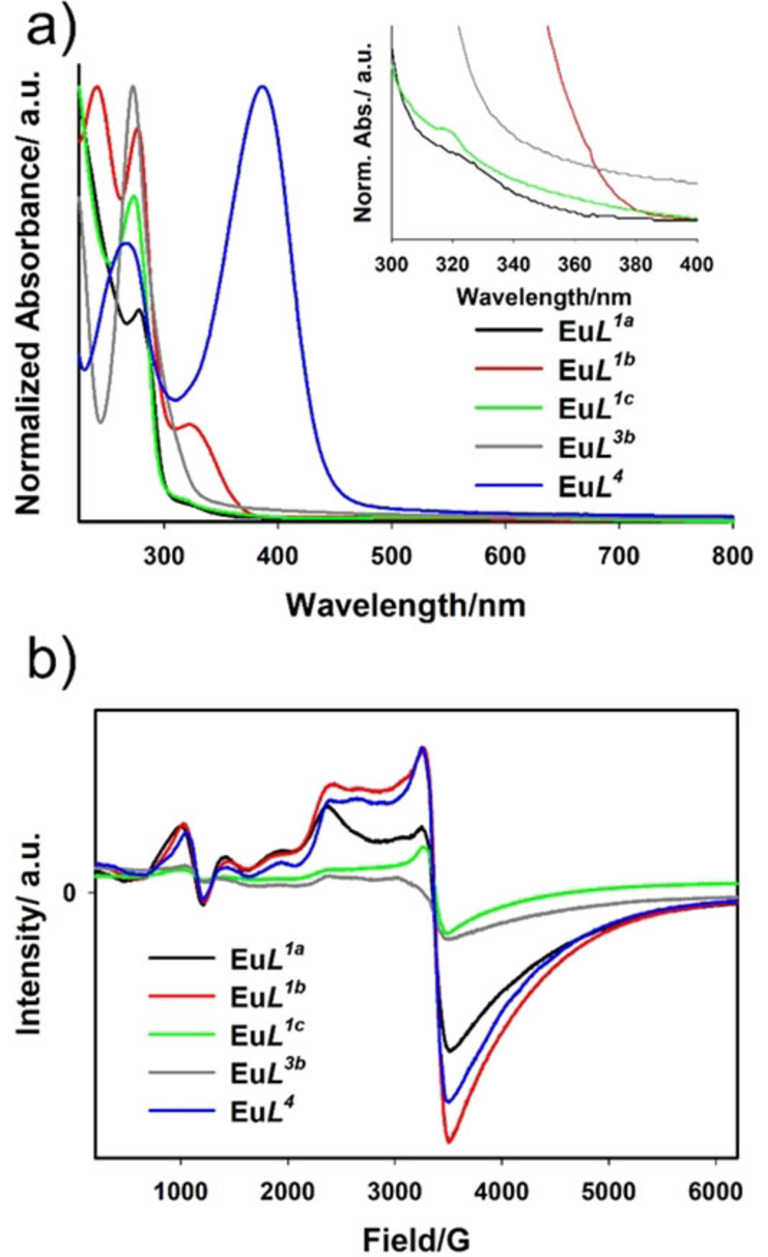
a) UV‐visible spectra of [Eu ***L*** ^***1a‐c***^] [Eu ***L*** ^***3b***^] and [Eu ***L*** ^***4***^] following reduction with zinc in methanol at ambient temperature (inset: expansion of the 300–400 nm range) and b) EPR spectra in methanol at 100 K.

Methanolic solutions of complexes [Eu ***L*** ^***1a‐c***^]^3+^, [Eu ***L*** ^***3b***^], and [Eu ***L*** ^***4***^] were examined using UV‐visible, luminescence, and EPR spectroscopies after reduction in the presence of Zn^0^. UV‐visible spectra of [Eu ***L*** ^***1a***^]^3+^ and [Eu ***L*** ^***1c***^]^3+^ show two new bands after reduction, and the other compounds did not have observable differences in their spectra between before and after reduction. The two new bands include a 4*f*–5*d* absorbance at 350 nm (seen as a shoulder due to ligand‐based absorbance) and a broad band at 575 nm (Figures 5a and S46, Supporting Information). Luminescence spectroscopy also displays broad 5*d*–4*f* emission bands at 430 and 440 nm for [Eu ***L*** ^***1a***^] (*λ*_ϵexc_ 350 nm) and [Eu ***L*** ^***1c***^] (*λ*_ϵexc_ 375 nm) respectively, as well as a lack of emission from Eu(III). The other complexes showed strong emission from Eu(III) in addition to weak 5*d*–4*f* emissions at 430 [Eu ***L*** ^***1b***^], 460 [Eu ***L*** ^***3b***^], and 475 nm [Eu ***L*** ^***4***^], (Figure S47, Supporting Information). X‐band EPR spectroscopy at 100 K of all five complexes have features characteristic of Eu(II) complexes, including bands at approximately 1100, 2200, and 3400 G (Figure 5b). Sharp bands related to organic radicals are not observed. These results suggest that each of the five complexes can be reduced from Eu(III) to Eu(II) to some extent, and that the late‐stage modifications of the precursor Eu(III) complexes impart tunability to the luminescence properties of the Eu(II) species.

### EPR properties of the Gd complexes

Paramagnetic Gd(III) complexes have a significant zero‐field splitting (ZFS) interaction, characterised by two parameters, *D* and *E*, with values that are postulated to be related to the symmetry and rigidity of the coordination environment. In high symmetry, |*D*| is near zero, and |*E*|≤|*D*/3|.^[38]^ At Q‐band (34 GHz, *B*_0_ 1.21 T) EPR frequencies and above, complexes with smaller *D* values are in the high field limit, i.e. *D*≪*gμ*_B_ *B*_0_. In this case, the frozen solution (powder) EPR absorption spectrum is broad and featureless for the various transitions due to a distribution in the ZFS parameters, except for a relatively narrow central line from the −1/2 →+1/2 transition. The width of the central transition depends on *D* to second order and is proportional to D^2^/*gB*_0_.

**Figure 6:**
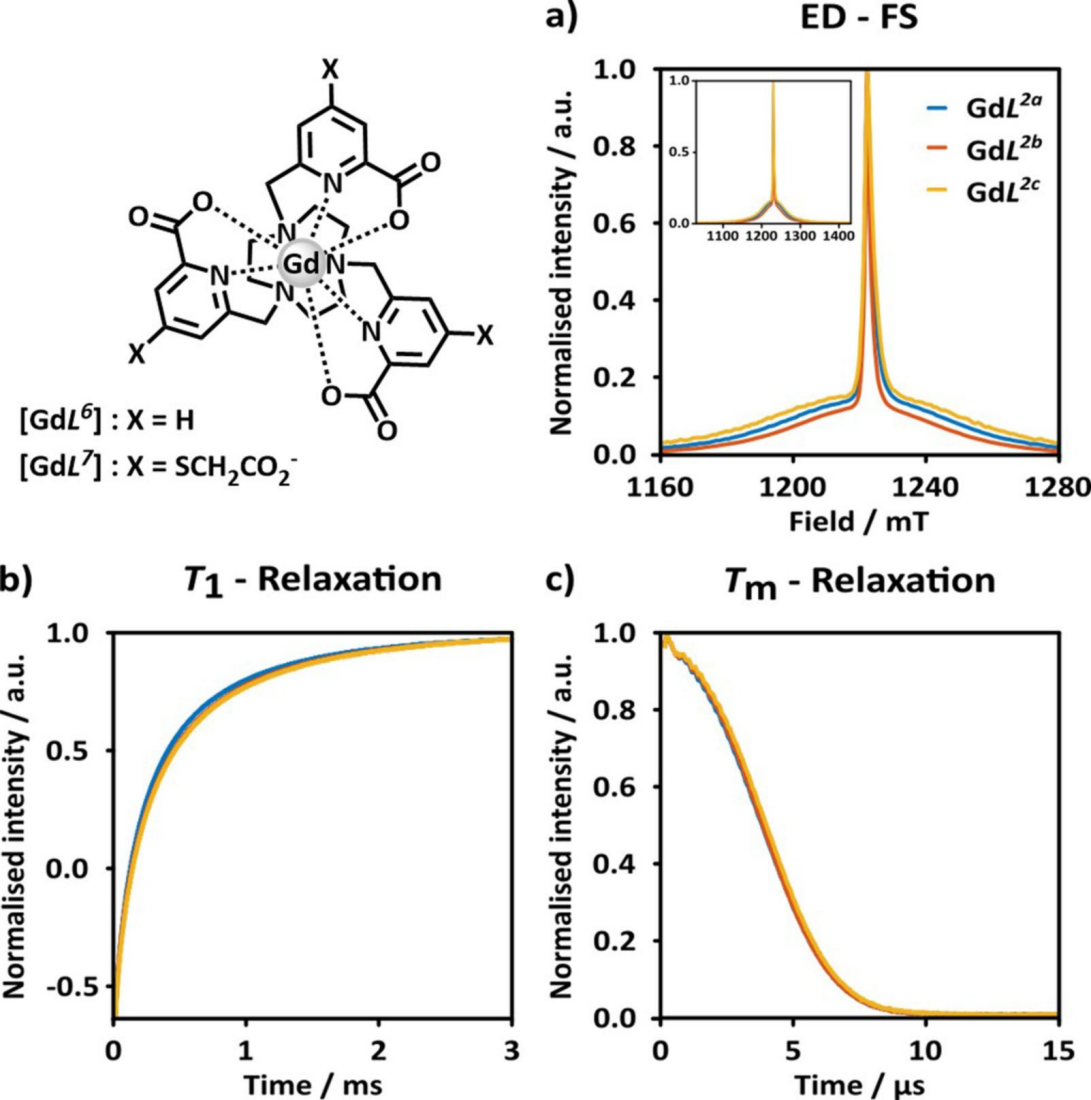
EPR characterisation of the Gd(III) complexes. Measurements taken at Q‐band, 10 K and normalised to their maximum. a) Echo‐Detected Field‐Swept (ED‐FS) data for the Gd(III) complexes for [Gd ***L*** ^***2a***^] (*blue*), [Gd ***L*** ^***2b***^] (*red*), and [Gd ***L*** ^***2c***^] (*yellow*) in water/methanol/glycerol. The full sweep is shown in the insert with a close‐up view on the main axis. b) Inversion recovery data for the Gd(III) complexes to measure *T*_1_. c) Echo decay curves to measure the transverse relaxation time *T*_m_, with time axis 2τ.

The echo‐detected field swept data and electron relaxation times (*T*_1_ and *T*_m_) for the Gd series [Gd ***L*** ^***2a***^], [Gd ***L*** ^***2b***^], and [Gd ***L*** ^***2c***^] were measured at Q‐band and 10 K in a water/methanol/glycerol mixture. Despite the difference in the electron withdrawing properties of the nitro, azide and amine groups the spectroscopic properties remain remarkably similar. The results are shown in Figure 6 and Table 2; further details, including fitting, are given in the Supporting Information. All three have an identical effective *g*‐value of 1.992 and possess almost identical relaxation times. It has been suggested earlier[^28^, ^29^] that the magnitude of the ZFS and the width of the central transition (−1/2 to +1/2 ) may be sensitive to the nature of the pyridine 4‐substituent. The azide complex, [Gd ***L*** ^***2b***^], was found to have the smallest ZFS (FWHH of the central transition 1.94 mT) while the other two complexes behaved similarly and were only slightly broader (FWHH 2.78 and 3.41 mT for nitro and amine respectively). These values are in line with previous results, where values of 1.72 mT and 2.10 mT were found for the C_3_‐symmetric complexes, [Gd ***L*** ^***6***^] and [Gd ***L*** ^***7***^], compared to 3.58 mT for [Gd ***L*** ^***2a***^] (hitherto termed [Gd.sTPATCN]‐SL) previously measured in water/glycerol at 10 K and Q‐band frequency.^[28]^ The EPR behaviour of the nitroxide‐ conjugate, [Gd ***L*** ^***5***^] will be reported elsewhere.

**Table 2:** Characteristic parameters found from analysis of the EPR data

Complex	*D*/MHz^[a]^	FWHH/mT^[b]^	*T*_1_/ms^[c]^	*T*_m,10%_/μs^[d]^
[Gd*L* ^*2a*^]	603±49	2.78	0.23	6.53
[Gd*L* ^*2*b^]	533±39	1.94	0.27	6.50
[Gd*L* ^*2c*^]	668±55	3.41	0.29	6.54

[a] The ZFS parameter *D* found from fits to one set of experimental data (MHz),^[38]^ see Supporting Information for further details. [b] FWHH of the central transition line of the echo‐detected field‐swept spectra. [c] *T*_1_ estimate assuming a single exponential decay. [d] Measure of *T*_m_ given by the time taken for the echo to decay to 10 % of its initial value. More precise values for *T*_1_ and *T*_m_ from fitting the EPR measurements are given in the Supporting Information which also contains matching parameters from sample repeats.

### Summary and Conclusions

The *p*−Br and *p*−NO_2_ pyridine coordination complexes, [Eu ***L*** ^***1a‐c***^], [Ln ***L*** ^***2a‐2d***^] and [Eu ***L*** ^***3a***^] serve as versatile synthetic precursors. The displacement of the *para* substituent occurs with a range of charged and uncharged nucleophiles in a nucleophilic aromatic substitution reaction that takes place under mild conditions in protic media, exemplified by the synthesis of the azide derivative from the nitro precursor in water at ambient temperature. Such a late stage functionalisation strategy obviates separate time‐consuming ligand syntheses. The nature of the *p*‐substituent can have a significant effect on the absorption and emission spectral properties of the Eu(III) complexes, particularly where lone pair conjugation occurs, highlighted in cationic complexes where the Eu ion serves as a powerful charge sink. Thus, the dimethylamino‐substituted complex, [Eu ***L*** ^***1b***^]^3+^ possesses a well‐defined absorption band at 350 nm, permitting selective excitation above 340 nm. This LMCT transition is associated with a buildup of negative charge on the pyridyl N atom, an increased local dipole moment and a strengthened overall Eu−N_py_ electrostatic interaction. Indeed, the Eu−N_py_ bond length in the X‐ray structure of [Eu ***L*** ^***1b***^]^3+^ was 0.05 Å shorter than in the *p*−Br and *p*−H analogues, [Eu ***L*** ^***1a***^]^3+^ and [Eu ***L*** ^***1c***^]^3+^, where the LMCT transition is not evident above 300 nm (Figure 1 vs. Figure 2). The Eu(III) complexes, [Eu ***L*** ^***1a‐c***^], [Eu ***L*** ^***3b***^], and [Eu ***L*** ^***4***^] are reduced from Eu(III) to Eu(II) using Zn^0^, and the ligand differences imparted to the Eu(III) complexes prior to reduction influence the luminescence properties after reduction.

The narrow linewidths, small ZFS values and long transverse relaxation times (*T*_m_) of the parent and modified Gd complexes are attractive properties for spin labels and for distance measurements using EPR methods, since these properties confer detection sensitivity. The ease of the substitution chemistry and similarity in the EPR lineshape and relaxation times of the gadolinium complexes, at 10 K and Q‐band frequency, following variation of the pyridine 4‐substituent, demonstrate that this ligand framework offers a convenient starting point for adding functionality and therefore serves as the basis of a versatile spin label.

The Supporting Information includes experimental details of ligand and complex synthesis, characterisation by NMR, MS and HPLC data; x‐ray structural refinement and spectroscopic data.

## Conflict of interest

The authors declare no conflict of interest.

## Supporting information

As a service to our authors and readers, this journal provides supporting information supplied by the authors. Such materials are peer reviewed and may be re‐organized for online delivery, but are not copy‐edited or typeset. Technical support issues arising from supporting information (other than missing files) should be addressed to the authors.

Supporting Information
