# Supplementary material for: Versatile Para‐Substituted Pyridine Lanthanide Coordination Complexes Allow Late Stage Tailoring of Complex Function
Source: Chemistry. 2021 Nov 16;27(71):17921–7. doi: 10.1002/chem.202103243 (PMC8688332; doi:10.1002/chem.202103243)
Supplement: Supplementary file 1 — Supporting Information [file CHEM-27-17921-s001.pdf]

Chemistry–A European Journal

Supporting Information

Versatile Para-Substituted Pyridine Lanthanide Coordination Complexes Allow Late Stage Tailoring of Complex Function

Matthieu Starck,* Jack D. Fradgley, Davide F. De Rosa, Andrei S. Batsanov, Maria Papa, Michael J. Taylor, Janet E. Lovett, Jacob C. Lutter, Matthew J. Allen, and David Parker*

Reagents 3
Analytical Methods 3
Synthesis of ligands and complexes 4
Synthesis of Ligand L^{1a} and Complex $[[EuL^{1a}]^{3+}.(CF_3SO_3^-)_3]$ and $[[EuL^{1b}]^{3+}.(Br^-)_2 (CF_3SO_3^-)]$ 5
Synthesis of Complexes $[EuL^{2a-d}]$ 8
Synthesis of Complexes $[GdL^{2b}]$ and $[GdL^{2c}]$. 10
Synthesis of complex $[EuL^{3b}]$ 10
Synthesis of complex $[EuL^{4}]$ 11
Synthesis of complex $[GdL^{5}]$ 14
NMR Spectra 15
HPLC Traces 31
Optical measurements and photophysical data of Eu(III) complexes 37
X-ray diffraction 44
Eu(II) complexes characterisation 45
Electron paramagnetic resonance of Gd(III) complexes 47
EPR ED-FS Fits 50
EPR Relaxation Fits 55
References 58

Reagents

All reagents were purchased and used as received. Analytical solvents were purchased from Fisher Scientific and Sigma Aldrich and were HPLC grade. Anhydrous solvents were freshly distilled over the appropriate drying agent and stored under argon in a septum-capped bottle or purchased and used as received. Water was purified by the 'Purite$_{STILL}$plus' system, with conductivity of ≤ 0.04 μS cm^{-1}. Air sensitive reactions were carried out under an atmosphere of argon using Schlenk-line techniques.

Analytical Methods

Thin layer Chromatography was carried out on neutral aluminium silica plates (Merck 5554) or neutral aluminium oxide plates (Merck 5550) and visualised under UV irradiation (254/365 nm). Preparative column chromatography was performed using silica gel (Merck Silica Gel 60, 230-400 mesh) or neutral aluminium oxide (Merck 90, 70-320 mesh).

NMR Spectroscopy. Proton (^{1}H), carbon (^{13}C), and phosphorus (^{31}P) NMR spectra were recorded on Bruker Avance III-HD-400 spectrometers with operating frequencies of 399.95 MHz or 400.07 MHz for ^{1}H and 100.57 MHz or 100.60 MHz for ^{13}C or Bruker Neo-400 spectrometers with operating frequencies of 400.20 MHz for ^{1}H and 100.63 MHz for ^{13}C. The operating temperature of the spectrometers (usually 295 K) was measured with the aid of an internal calibration solution of ethylene glycol.

Melting points were recorded using a Gallenkamp (Sanyo) apparatus and are uncorrected.

Reverse phase preparative HPLC purification was performed at 295 K using a Shimadzu system consisting of a Degassing Unit (DGU-20A$_{5R}$), a Prominence Preparative Liquid Chromatograph (LC-20AP), a Prominence UV/Vis Detector (SPD-20A) and a Communications Bus Module (CBM-20A). An XBridge C18 OBD 19 × 100 mm, i.d. 5 μM column was used with a flow rate of 17 mL/min (prep). Conditions were:

- ***A.*** The 17 ml/min solvent gradient ran from 10% acetonitrile in water both containing 0.1% formic acid to 100% acetonitrile over 10 minutes

B. The 17 ml/min solvent gradient ran from 10% acetonitrile in ammonium bicarbonate buffer (25 mM in water, pH = 7.4) to 100% acetonitrile over 10 minutes.

Mass spectrometry analyses were performed on a QToF Premier equipped with an Acquity UPLC (Waters Corp.). Reverse phase gradient separation was achieved using an Acquity UPLC BEH C18 column 1.7 µm (2.1 mm × 100 mm) (Waters Corp.). The 0.4 mL/min solvent gradient ran from 100% water containing 0.1% formic acid to 100% acetonitrile over 5 minutes. Positive ions from the electrospray ion source were recorded as a full MS spectrum. The desired precursor ion was mass selected by the quadrupole with an isolation window that transmitted the full isotopic envelope. A reference spray provided a 'lock mass' that enable a calibration correction for accurate mass determination of the MS data.

Synthesis of ligands and complexes

Methyl 4-bromo-6-(hydroxymethyl)picolinate **1**,[1] ethyl (4-bromo-6-(hydroxymethyl) pyridin-2-yl)(methyl)phosphinate **7**,[2] the cyclooctyne spin label 1-fluoro-N-[(1-oxyl-2,2,5,5-tetramethyl-2,5-dihydro-1H-pyrrol-3-yl)methyl]cyclooct-2-ynecarboxamide radical ***12***,[3] complex **[EuL^{1c}]$^{3+}$.($CF_3SO_3^-$)$_3$**,[4] ligand ***L^{2a}***,[5] complex **[GdL^{2a}]**,[5] complex **[EuL^{3a}]**[2] were prepared according to the literature.

(*S*)-(-)-α-methylbenzylamine **3** and 4-ethynyl-*N*,*N*-dimethylaniline **8** were purchased from Merck and triazacyclononane trihydrocloride **6** was purchased from Chematech.

Synthesis of Ligand L^{1a} and Complex $[[EuL^{1a}]^{3+}.(CF_3SO_3^-)_3]$ and $[[EuL^{1b}]^{3+}.(Br^-)_2(CF_3SO_3^-)]$

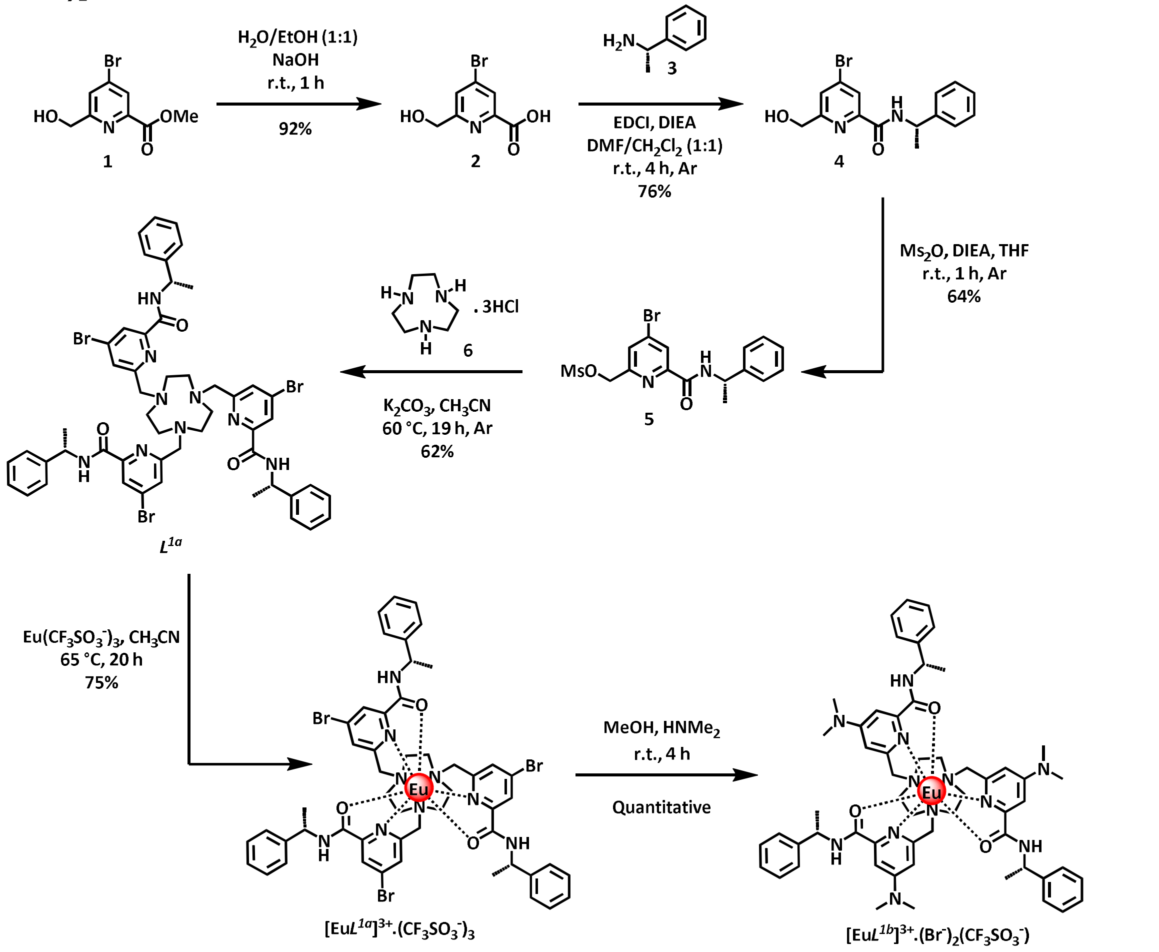

Synthesis of 4-bromo-6-(hydroxymethyl)picolinic acid, 2. Methyl 6-(hydroxymethyl)picolinate **1** (300 mg, 1.22 mmol) was dissolved in aqueous ethanol (12 ml, 1:1) followed by the slow addition of NaOH pellets (97 mg, 2.44 mmol). The solution was stirred for 1 h at room temperature. After this time, the solvent was removed under reduced pressure and the residue dissolved in water (5 mL) and acidified to pH 4. The resulting solution was extracted using EtOAc (4 × 50 ml) and the combined organic layers dried over Na_2SO_4 before removal of the solvent under reduced pressure to afford a white solid (259 mg, 92%). TLC analysis **R_f** 0.16 (silica, 15% MeOH in DCM); **UPLC** (CH_3CN/H_2O, 0.1% FA) t_R = 0.99 min; **^{1}H NMR** (400 MHz, D_2O) δ_H 8.23 (1H, s), 8.05 (1H, s), 4.77 (2H, s); **^{13}C NMR** (101 MHz, D_2O) δ_C 164.6, 158.5, 146.9, 140.8, 128.4, 128.0, 60.9; **(HRMS$^+$)** *m/z* 231.9608 ($C_7H_7NO_3Br$ requires 231.9609).

Synthesis of (S)-4-bromo-6-(hydroxymethyl)-N-(1-phenylethyl)picolinamide, 4. Hydroxybenzotriazole hydrate (227 mg, 1.68 mmol), EDC (322 mg, 1.68 mmol), DIPEA (0.40 ml, 2.24 mmol), and (*S*)-(-)-α-methylbenzylamine **3** (0.15 ml, 1.23 mmol) were dissolved in anhydrous DMF:DCM (4 ml, 1:1). To this solution was added a solution of the carboxylic acid **2** (259 mg, 1.12 mmol) in DMF (1 mL) slowly dropwise. The reaction was stirred for 4 h at room temperature under argon. After this time, the solvent was removed under reduced pressure and water (5 mL) was added to the residue followed by repeated extraction with EtOAc (4 × 40 ml). The organic layers were combined, washed with water (40 ml), dried over Na_2SO_4, and the solvent was removed under reduced pressure to give a crude residue. This residue was purified by column chromatography over silica (MeOH in DCM, 0 to 3%) to afford a pale yellow oil (285 mg, 76%). TLC analysis $\mathbf{R}_f$ 0.49 (silica, 5% MeOH in DCM); **UPLC** (CH_3CN/H_2O, 0.1% FA) t_R = 2.87 min; **^{1}H NMR** (400 MHz, $CDCl_3$) δ_H 8.11 (1H, s), 7.99 (1H, d, *J* = 8 Hz), 7.86 (1H, s), 7.33 – 7.17 (5H, m), 5.28 – 5.18 (1H, m), 4.69 (2H, s), 1.54 (3H, d, *J* = 8 Hz); **^{13}C NMR** (101 MHz, $CDCl_3$) δ_C 162.5, 161.5, 149.9, 142.9, 135.0, 128.6, 127.3, 126.2, 124.2, 108.0, 64.3, 49.0, 21.7; **(HRMS$^+$)** *m/z* 335.0400 ($C_{15}H_{16}N_2O_2Br$ requires 335.0395).

Synthesis of (S)-(4-bromo-6-((1-phenylethyl)carbamoyl)pyridin-2-yl)methyl methanesulfonate, 5. The alcohol **4** (285 mg, 0.85 mmol) and methanesulfonic anhydride (371 mg, 2.13 mmol) were dissolved in anhydrous THF (3 ml) and DIEA (0.4 ml) was added. The mixture was left to stir for 1 h at room temperature under an argon atmosphere. The solvent was removed under reduced pressure, and DCM (10 ml) and water (10 ml) were added to the resulting residue. The aqueous layer was separated and further extracted with DCM (3 × 10 ml), and the combined organic layers were dried over Na_2SO_4. The solvent was removed under reduced pressure to afford compound 5 as a pale orange oil that was used directly without further purification (200 mg, 64%). TLC analysis $\mathbf{R}_f$ 0.64 (silica, 3% MeOH in DCM); **^{1}H NMR** δ_H 8.28 (1H, s), 8.21 (1H, d, *J* = 8 Hz), 7.72 (1H, s), 7.38 – 7.19 (5H, m), 5.31 – 5.25 (3H, m), 3.09 (3H, s), 1.59 (3H, d, *J* = 8 Hz).

Synthesis of Ligand, L^{1a}. Mesylate **5** (170 mg, 0.41 mmol) and 1,4,7-triazacyclononane trihydrochloride (28 mg, 0.12 mmol) were dissolved in anhydrous CH_3CN (2 ml). K_2CO_3 (97 mg, 0.70 mmol) was added and the reaction was heated at 60 °C for 19 h under argon. The solution was filtered to remove the salts and the

solvent was removed under reduced pressure. The product was purified by column chromatography over silica (MeOH in DCM, 0 to 3%), affording a clear bright orange oil (79 mg, 62%). TLC analysis **R_f** 0.13 (silica, 3% MeOH in DCM); **UPLC** (CH_3CN/H_2O, 0.1% FA) t_R = 3.47 min; **^{1}H NMR** (400 MHz, $CDCl_3$) δ_H 8.34 (3H, d, J = 8 Hz), 8.27 (3H, s), 7.79 (3H, s), 7.41 – 7.23 (15H, m), 5.37 – 5.30 (3H, m), 3.79 (6H, s), 2.82 (12H, s), 1.63 (9H, d, J = 8 Hz); **^{13}C NMR** (101 MHz, $CDCl_3$) δ_C 162.3, 160.6, 153.7, 150.5, 143.0, 134.9, 128.7, 128.5, 127.4, 126.3, 124.4, 63.8, 56.0, 48.9, 21.8; **(HRMS$^+$)** *m/z* 1078.1975 ($C_{51}H_{54}Br_3N_9O_3$ requires 1078.1978).

Synthesis of complex [EuL^{1a}]$^{3+}$ ($CF_3SO_3^-$)$_3$. Europium (III) triflate (13.1 mg, 0.022 mmol) and ***L^{1a}*** (23.5 mg, 0.022 mmol) were dissolved in anhydrous CH_3CN (1.5 ml) and the mixture was stirred at 65 °C for 20 h. The solution volume was reduced by half under reduced pressure and was slowly added dropwise to cold diethyl ether (30 ml) causing a white precipitate to form. The solvent was carefully decanted, and the precipitate dried under reduced pressure to afford a white glassy solid (20 mg, 75%). **^{1}H NMR** (200 MHz, CD_3OD) δ_H 7.89 (1H, s, py-CHN), 7.40 (4H, br. s, Hmeta + py-H_3 + py-H_5), 6.98 (2H, s, H_{ortho}), 6.48 (1H, s, H_{para}), 3.86 (1H, s, C*H*Me), 3.26 (1H, s, $H_{ax'}$), 1.15 (3H, s, CMe), -1.24 (1H, s, py-CH'N), -1.76 (1H, s, H_{eq}), -2.83 (1H, s, $H_{eq'}$), -6.84 (1H, s, H_{ax}); **UPLC** (CH_3CN/H_2O, 0.1% FA) t_R = 1.92 min; **(HRMS$^+$)** *m/z* 1230.1109 ($C_{51}H_{54}{}^{151}EuBr_3N_9O_3$ requires 1230.1112); λ_{abs} (MeOH) 280, 290 nm (ε 13000 M^{-1} cm^{-1}); τ (MeOH) 0.75 ms; ϕ_{Eu} (MeOH) = 0.6%.

The complex was dissolved in methanol/water (1:1, 2 ml total) and left to crystallize at room temperature. After 20 h, crystals had formed and an X-ray crystal structure was obtained at 120 K. **Crystal Data** for $C_{54.5}H_{56.003333}Br_3EuF_9N_9O_{12.5}S_3$ (M =1695.95 g/mol): trigonal, space group R3 (no. 146), a = 21.9132(17) Å, c = 11.3297(10) Å, V = 4711.5(8) Å^3, Z = 3, T = 120 K, μ(MoKα) = 3.101 mm^{-1}, $Dcalc$ = 1.793 g/cm^3, 32015 reflections measured (4.186° ≤ 2Θ ≤ 60.228°), 6165 unique (R_{int} = 0.0478, R_{sigma} = 0.0509) which were used in all calculations. The final R_1 was 0.0295 (I > 2σ(I)) and wR_2 was 0.0661 (all data).

Synthesis of the complex [EuL^{1b}]$^{3+}$.(Br^-)$_2$($CF_3SO_3^-$). The triflate salt of the complex **[EuL^{1a}]$^{3+}$** (15 mg, 0.01 mmol) was dissolved in anhydrous MeOH (0.5 ml) and dimethylamine (0.83 ml, 5.6 mM) was added. The mixture was left to stir for 4 h when the solvent was removed under reduced pressure to yield a white solid (11 mg). **^{1}H**

NMR (200 MHz, CD_3OD) δ_H 7.09 (3H, br. m, py-CHN + H_{meta}), 6.90 (2H, br. s, H_{ortho}), 5.47 (2H, br. m, $CH_{ax'}$ +H_{para}), 4.98 (1H, br. q, C*H*Me), 4.89 (1H, br. s, H_3), 4.36 (1H, s, H_5), 3.49 (6H, s, NMe_2), 1.49 (3H, br. d, MeC), 0.79 (1H, s, $H_{eq'}$), 0.60 (1H, s, H_{eq}), -0.96 (1H, s, py-CHN), -5.66 (1H, s, H_{ax}); **(HRMS$^+$)** *m/z* 1125.5059 ($C_{57}H_{72}EuN_{12}O_3$ requires 1125.5063); λ_{abs} (MeOH) 290 nm (ε 12000 M^{-1} cm^{-1}), 340 nm (ε 3700 M^{-1} cm^{-1}); τ (MeOH) 0.76 ms; ϕ_{Eu} (MeOH) 1.3%.

Single crystals of $C_{59}H_{78}Br_2EuF_3N_{12}O_8S$ were grown in aqueous methanol from the crude reaction mixture containing the dimethylammonium hydrobromide salt. A suitable crystal was selected and mounted on a Bruker D8 Venture diffractometer. The crystal was kept at 120 K during data collection. The crystal isolated was the dibromide-mono-triflate complex. **Crystal Data** for $C_{59}H_{78}Br_2EuF_3N_{12}O_8S$ (M =1484.55 g/mol): monoclinic, space group $P2_1$ (no. 4), a = 13.6926(8) Å, b = 23.9163(14) Å, c = 19.3436(11) Å, β = 90.739(2)°, V = 6334.0(6) Å^3, Z = 4, T = 120 K, μ(MoKα) = 2.359 mm^{-1}, $Dcalc$ = 1.557 g/cm^3, 165928 reflections measured (4.002° ≤ 2Θ ≤ 65.836°), 46976 unique (R_{int} = 0.0425, R_{sigma} = 0.0545) which were used in all calculations. The final R_1 was 0.0363 (I > 2σ(I)) and wR_2 was 0.0794 (all data).

Synthesis of Complexes [EuL^{2a-d}]

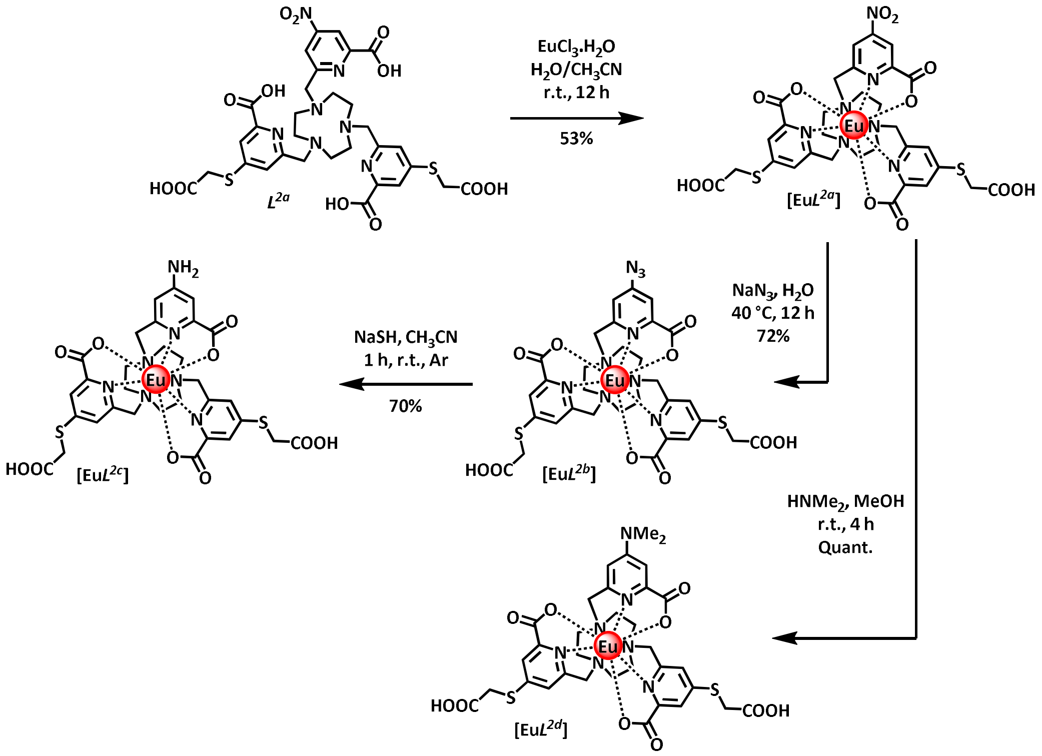

Synthesis of complex [Eu*L*^{2a}]. The hexa-acid ***L*^{2a}** (23.6 mg, 31.1 μmol) was dissolved in water/CH_3CN (2 mL, 1:1) at room temperature and the pH was adjusted to 6. The salt $EuCl_3.6H_2O$ (12.7 mg, 34.2 μmol) was added, and the pH was increased to 7 and the solution stirred at room temperature for 24 h. The solvent was removed under reduced pressure and the residue was purified by preparative reverse-phase HPLC (condition B) to afford a white solid (15 mg, 53%). UPLC (CH_3CN/H_2O, 0.1% FA) **t_R** = 1.09 min; **(HRMS$^+$)** *m/z* = 908.0656 $[M+H]^+$ ($C_{31}H_{31}{}^{151}EuN_7O_{12}S_2$ requires 908.0671); λ_{abs} (H_2O) 284 nm (ε 15000 M^{-1} cm^{-1}); τ (H_2O) 1.26 ms; ϕ_{Eu} (H_2O) <0.05%.

Synthesis of complex [Eu*L*^{2b}]. Complex **[Eu*L*^{2a}]** (8.4 mg, 8.4 μmol) was dissolved in water (3 mL) and NaN_3 (100 mg, 1.54 μmol) was added. The reaction mixture was stirred at 40 °C for 48 h with monitoring of the reaction by LC/MS and further addition of NaN_3 (50 mg, 0.77 μmol). A white solid (6.0 mg, 72%) was afforded after purification using HPLC (condition A). **(HRMS+)** m/z 904.0858 $[M+H]^+$ ($C_{31}H_{31}{}^{151}EuN_9O_{10}S_2$ requires 904.0834); **UPLC** (CH_3CN/H_2O, 0.1% FA) t_R = 0.96 min; λ_{abs} (H_2O) 276 nm (ε 22000 M^{-1} cm^{-1}); τ(H_2O) 0.90 ms; ϕ_{Eu} (H_2O) 0.4%.

Synthesis of complex [Eu*L*^{2c}]. Complex **[Eu*L*^{2b}]** (4 mg, 4.0 μmol) was dissolved in anhydrous CH_3CN (1 mL) and the solution was degassed by bubbling argon through the solution for 15 min. NaHS (25 mg, 0.45 mmol) was added and the reaction mixture was stirred at room temperature for 1 h. A pale-brown solid (2.7 mg, 70%) was isolated after purification using HPLC, using condition A. **(HRMS+)** *m/z* 878.0933 $[M+H]^+$ ($C_{31}H_{33}{}^{151}EuN_7O_{10}S_2$ requires 878.0929); **UPLC** (CH_3CN/H_2O, 0.1% FA) t_R = 0.90 min; λ_{abs} (H_2O) 280 nm (ε 19000 M^{-1} cm^{-1}); τ(H_2O) 0.40 ms; ϕ_{Eu} (H_2O) 1%.

Synthesis of complex [Eu*L*^{2d}]. Complex **[Eu*L*^{2a}]** (1.5 mg, 1.66 μmol) was dissolved in anhydrous MeOH (0.5 ml) and dimethylamine (2 M in THF, 0.5 mL, 1 mM) was added. The mixture was left to stir for 4 h when the solvent was removed under reduced pressure to yield a white solid (1.5 mg); **(HRMS+)** *m/z* 906.1249 $[M+H]^+$ ($C_{33}H_{37}{}^{151}EuN_7O_{10}S_2$ requires 906.1242); **UPLC** (CH_3CN/H_2O, 0.1% FA) t_R = 1.35 min; λ_{abs} (H_2O) 282 nm (ε 18000 M^{-1} cm^{-1}); τ(H_2O) 0.74 ms; ϕ_{Eu} (H_2O) <0.05%.

Synthesis of Complexes [GdL^{2b}] and [GdL^{2c}].

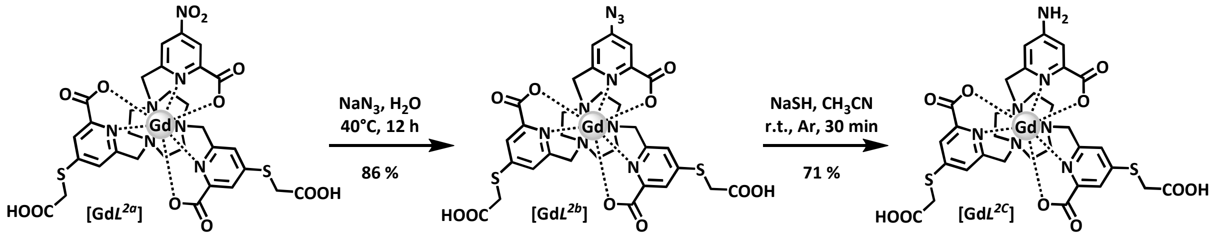

Synthesis of complex [Gd*L^{2b}*]. Complex **[Gd*L^{2a}*]** (20 mg, 21.9 µmol) was dissolved in water (5 mL) and NaN_3 (144 mg, 21.9 mmol) was added. The reaction mixture was stirred at 40 °C for 12 h. A pale brown solid (17.2 mg, 86%) was isolated after purification using HPLC, condition A. **(HRMS+)** *m/z* 907.0844 $[M+H]^+$ ($C_{31}H_{31}GdN_9O_{10}S_2$ requires 907.0824); **UPLC** (CH_3CN/H_2O, 0.1% FA) t_R = 0.95 min.

Synthesis of complex [Gd*L^{2c}*]. Complex **[Gd*L^{2b}*]** (5 mg, 5.5 µmol) was dissolved in anhydrous MeCN (1 mL) and the solution was degassed by bubbling argon through the solution for 15 min. NaHS (31 mg, 0.55 mmol) was added and the reaction mixture was stirred at room temperature for 30 min. A dark grey solid (3.5 mg, 71%) was isolated after purification using HPLC, using condition A. **(HRMS+)** *m/z* 881.0954 $[M+H]^+$ ($C_{31}H_{33}GdN_7O_{10}S_2$ requires 881.0939); **UPLC** (CH_3CN/H_2O, 0.1% FA) t_R = 1.105 min.

Synthesis of complex [EuL^{3b}]

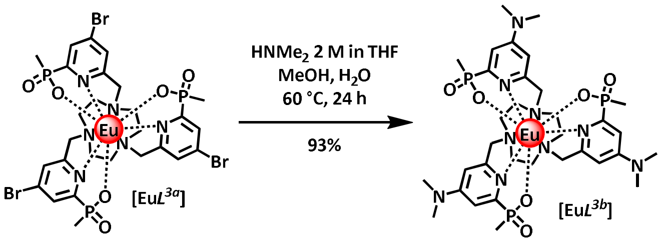

The complex **[Eu*L^{3a}*]** (9 mg, 8.8 µmol) was dissolved in a $MeOH/H_2O$ solution (4 mL, 1:1) and dimethylamine (2 M in THF, 0.8 mL, 1.6 mmol) was added. The solution was stirred at 60 °C for 12 h, before addition of further $HNMe_2$ (0.8 mL, 1.6 mmol) and the reaction was stirred at 60 °C for a further 12 h. The solvent was removed under reduced pressure and the residue was dissolved in MeOH (0.5 mL). A pale orange solid was isolated on precipitation of the complex by slow addition into cold Et_2O (7.5 mg, 93%). **UPLC** (CH_3CN/H_2O, 0.1% FA) t_R = 2.85 min; **(HRMS+)** *m/z* 914.2449 $[M+H]^+$ ($C_{33}H_{52}{}^{151}EuN_9O_6P_3$ requires 914.2452); **UPLC** (CH_3CN/H_2O, 0.1% FA) t_R =

1.17 min, λ_{max} (MeOH) 273 nm (ε 11000 M^{-1} cm^{-1}), τ (MeOH) = 0.83 ms, ϕ_{Eu} (H_2O) = 1.7%.

Synthesis of complex [EuL⁴]

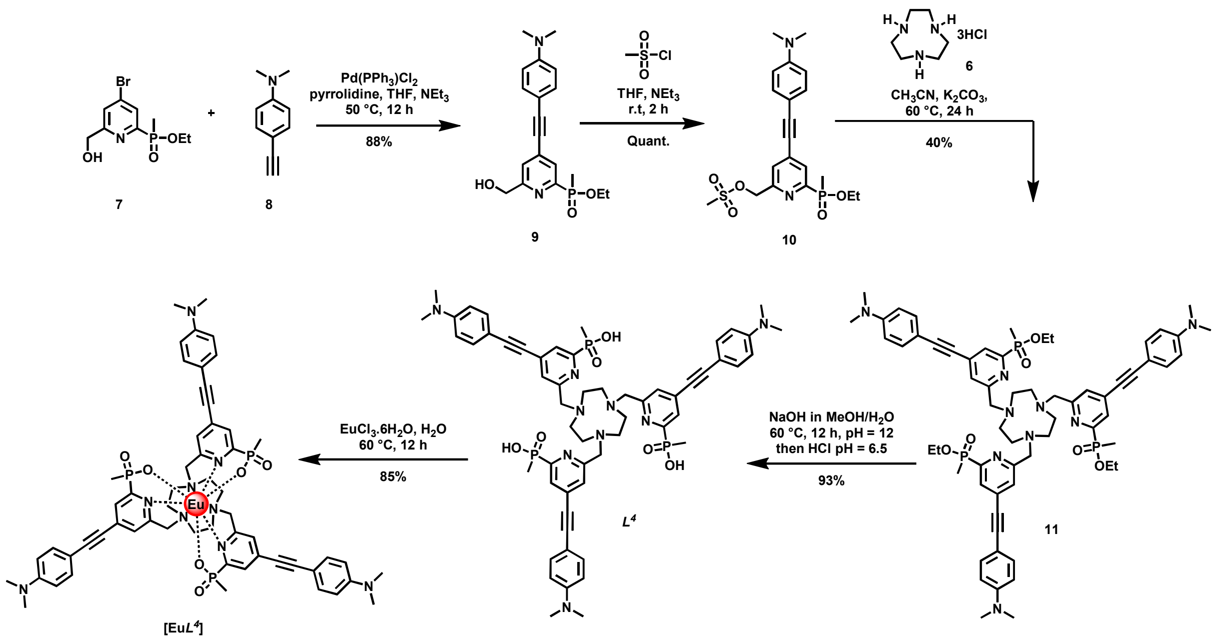

Ethyl (4-((4-(dimethylamino)phenyl)ethynyl)-6-(hydroxymethyl)pyridin-2-yl)(methyl)phosphinate, 9. To a solution of ethyl (4-bromo-6-(hydroxymethyl)pyridin-2-yl)(methyl)phosphinate **7** (128 mg, 0.43 mmol) and 4-ethynyl-*N,N*-dimethylaniline **8** (126 mg, 0.87 mmol) in anhydrous THF (2 mL) under argon was added pyrrolidine (186 µL, 2.2 mmol) and $Pd(PPh_3)_2Cl_2$ (32 mg, 0.04 mmol). The reaction mixture was stirred at 50 °C for 24 h, after which the solvent was removed under reduced pressure and DCM/water (20 mL, 1:1) was added to the residue. The aqueous layer was extracted with DCM (3 x 10 mL) and the combined organic layers were dried over Na_2SO_4 and the solvent removed under reduced pressure to yield a brown residue which was purified by column chromatography (Al_2O_3, neat DCM to 2% MeOH in DCM) to afford compound 9 as a golden oil (136 mg, 88%). **^{1}H-NMR** (400 MHz, $CDCl_3$) δ 7.98 (1H, br. d, *J* = 6 Hz), 7.49 (1H, s), 7.40 (2H, d, *J* = 9 Hz), 6.65 (2H, d, *J* = 9 Hz), 4.80 (2H, s), 4.15 – 3.84 (2H, m), 3.00 (3H, s), 1.79 (3H, d, *J* = 15 Hz), 1.27 (3H, t, *J* = 7 Hz); **^{13}C-NMR** (101 MHz, $CDCl_3$) δ 160.8 (d, *J* = 20 Hz), 152.6 (d, *J* = 160 Hz), 150.8, 133.7 (d, *J* = 11 Hz), 133.4, 127.8 (d, *J* = 22 Hz), 123.8 (d, *J* = 3 Hz), 111.7, 107.9, 98.2, 85.5 (d, *J* = 2 Hz), 64.1, 61.3 (d, *J* = 6 Hz), 40.1, 16.4 (d, *J* = 6 Hz), 13.4 (d, *J* = 105 Hz); **^{31}P{^{1}H}-NMR** (162 MHz, $CDCl_3$) δ 40.24; **UPLC**

(CH_3CN/H_2O, 0.1% FA) t_R = 2.85 min; **(HRMS+)** *m/z* 359.1526 $[M+H]^+$ ($C_{19}H_{23}N_2O_3P$ requires 359.1525).

(4-((4-(Dimethylamino)phenyl)ethynyl)-6-(ethoxy(methyl)phosphoryl)pyridine-2-yl)methyl methane-sulfonate, 10. The alcohol **9** (70 mg, 0.19 mmol) was dissolved in anhydrous THF (3 mL) under argon. Mesyl chloride (22 µL, 0.30 mmol) and NEt_3 (88 µL, 0.65 mmol) were added under argon and the reaction was stirred at room temperature for 2 h. After this time, the solvent was removed under reduced pressure and DCM/water (1:1, 20 mL) added to the residue. The aqueous layer was extracted with DCM (3 × 10 mL) and the combined organic layers were dried over Na_2SO_4 before removal of the solvent under reduced pressure to yield the mesylate 10 as a dark yellow oil (82 mg, quant.) that was judged sufficiently pure to be used in the next step without further purification. **^{1}H NMR** (400 MHz, $CDCl_3$) δ_H 8.01 (1H, dd, *J* = 6 Hz, *J* = 2 Hz), 7.56 (1H, s), 7.39 (2H, d, *J* = 9 Hz), 6.63 (2H, d, *J* = 9 Hz), 5.33 (2H, s), 4.15 – 4.05 (2H, m), 3.11 (3H, s), 2.99 (6H, s), 1.74 (3H, d, *J* = 15 Hz), 1.25 (3H, t, *J* = 7 Hz).

Triethyl-(((1,4,7-triazacyclononane-1,4,7-triyl)tris(methyene))tris(4-((4-(dimethyl-amino)phenylethy-nyl)pyridine-6,2-diyl))tris(methylphosphinate), 11. 1,4,7-Triazacyclononane trihydrochloride (13.6 mg, 57 µmol) and the mesylate **10** (82 mg, 190 µmol) were dissolved in anhydrous CH_3CN (2 mL), and K_2CO_3 (26.3 mg, 190 µmol) was added. The mixture was stirred under argon at 60 °C for 12 h. Excess potassium salts were removed by filtration and the solvent was removed under reduced pressure. The subsequent residue was dissolved in DCM (20 mL) and water (10 mL) was added. The aqueous layer was extracted with DCM (3 × 10 mL). The combined organic layers were dried over $MgSO_4$ and the solvent removed under reduced pressure to yield the crude ligand precursor. Compound **11** (26.3 mg, 40%) was isolated by HPLC (ammonium bicarbonate buffer, 25 mM) as a pale-yellow oil. **^{1}H-NMR** (400 MHz, $CDCl_3$) δ 7.99 (3H, dd, *J* = 6 Hz, *J* = 2 Hz), 7.68 (3H, t, *J* = 2 Hz), 7.42 (6H, d, *J* = 9 Hz), 6.66 (6H, d, *J* = 9 Hz), , 4.15 – 3.82 (6H, m), 3.98 (2H, br. s), 3.02 (18H, s), 3.00 (12H, s), 1.77 (9H, d, *J* = 15 Hz), 1.26 (9H, t, *J* = 7 Hz); **^{13}C-NMR** (101 MHz, $CDCl_3$) δ 160.9 (d, *J* = 20 Hz), 153.5 (d, *J* = 160 Hz), 150.8, 133.4, 133.2 (d, *J* = 12 Hz), 127.5 (d, *J* = 22 Hz), 126.4, 111.7, 108.1, 97.6, 85.2 (d, J= 2 Hz), 63.6, 61.0 (d, *J* = 6 Hz), 55.4, 40.1, 16.5 (d, *J* = 6 Hz), 13.4 (d, *J* = 105 Hz); **^{31}P{^{1}H}-**

NMR (162 MHz, $CDCl_3$) δ 40.16; **UPLC** (CH_3CN/H_2O, 0.1% FA) t_R = 3.36 min; **(HRMS+)** *m/z* 1150.537 $[M]^+$ ($C_{63}H_{78}N_9O_6P_3$ requires 1150.537).

(((1,4,7-Triazacyclononane-1,4,7-triyl)tris(methylene))tris(4-((4-(dimethylamino) phenyl)ethynyl)pyridine-6,2-diyl))tris(methylphosphinic acid), L^4. The ester **11** (26.3 mg, 22.9 µmol) was dissolved in a solution of MeOH/H_2O (2 ml, 1:1) and aqueous NaOH solution (2 M in water) was added slowly to reach pH 12. The solution was heated at 60 °C overnight before removal of the solvent under reduced pressure. The residue was dissolved in water (2 mL) and the pH was adjusted to 7 by addition of hydrochloric acid (1 M) solution. The solvent was removed under reduced pressure and the residue was dissolved in the minimum volume of MeOH, before Et_2O and THF were added in small aliquots to induce precipitation. A yellow powder was isolated by centrifugation (22.6 mg, 93%). **^{1}H-NMR** (400 MHz, CD_3OD) δ 7.83 (3H, app. d, J = 6 Hz,), 7.51 (3H, app. s), 7.38 (6H, d, J = 9 Hz), 6.71 (6H, d, J = 9 Hz), 3.96 (6H, br. s), 3.02 (18H, s), 2.89 (12 H, br. s), 1.52 (9H, d, J = 15 Hz); **^{31}P{^{1}H}-NMR** (162 MHz, $CDCl_3$) δ 26.27; **UPLC** (CH_3CN/H_2O, 0.1% FA) t_R = 3.24 min; **(HRMS+)** *m/z* 1066.446 $[M+H]^+$ ($C_{57}H_{66}N_9O_6P_3$ requires 1066.443).

Europium complex of (((1,4,7-triazacyclononane-1,4,7-triyl)tris(methyene))tris(4-((4-(dimethylamino)phenylethynyl)pyridine-6,2-diyl))tris(methylphosphinate), [EuL^4]. The ligand **L^4** (14 mg, 13.2 µmol) was dissolved in aqueous methanol (1 mL, 1:1) and $EuCl_3{\cdot}6\,H_20$ (5.3 mg, 14.5 µmol) was added. The solution was stirred at 60 °C for 12 h. The solution was removed under reduced pressure to yield the crude metal complex as a yellow solid that was purified by reverse-phase HPLC (ammonium bicarbonate buffer, 25 mM). A yellow powder (16 mg, 85%) was isolated after freeze-drying. **(HRMS+)** *m/z* 1214.338 $[M+H]^+$ ($C_{57}H_{63}{}^{151}EuN_9O_6P_3$ requires 1214.339); **UPLC** (CH_3CN/H_2O, 0.1% FA) t_R = 3.36 min, λ_{max} 388 nm; ε_{388nm} = 45 000 M^{-1} cm^{-1}, ε_{268nm} = 32 000 M^{-1} cm^{-1}.

Synthesis of complex [GdL⁵]

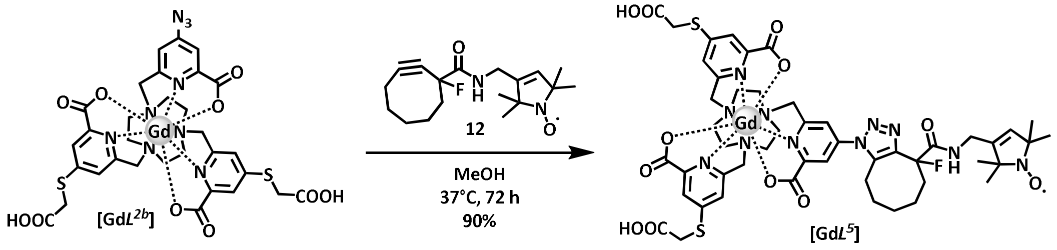

Complex **[Gd*L*^{2b}]** (2.8 mg, 3.11 µmol) was dissolved in methanol (1 mL). The cyclooctyne spin label, 1-fluoro-*N*-[(1-oxyl-2,2,5,5-tetramethyl-2,5-dihydro-1H-pyrrol-3-yl)methyl]cyclooct-2-ynecarboxamide **12** (2 mg, 6.22 µmol) was added and the reaction mixture was stirred at 37 °C for 72 h. The pure complex **[Gd*L*^{5}]** was obtained as a pale white solid (3.4 mg, 90%) after purification using HPLC with condition B. **(HRMS+)** *m/z* 1228.2797 $[M+H]^+$ ($C_{49}H_{57}FGdN_{11}O_{12}S_2$ requires 1228.2822); **UPLC** (CH_3CN/H_2O, 0.1% FA) t_R = 2.36 min.

NMR Spectra

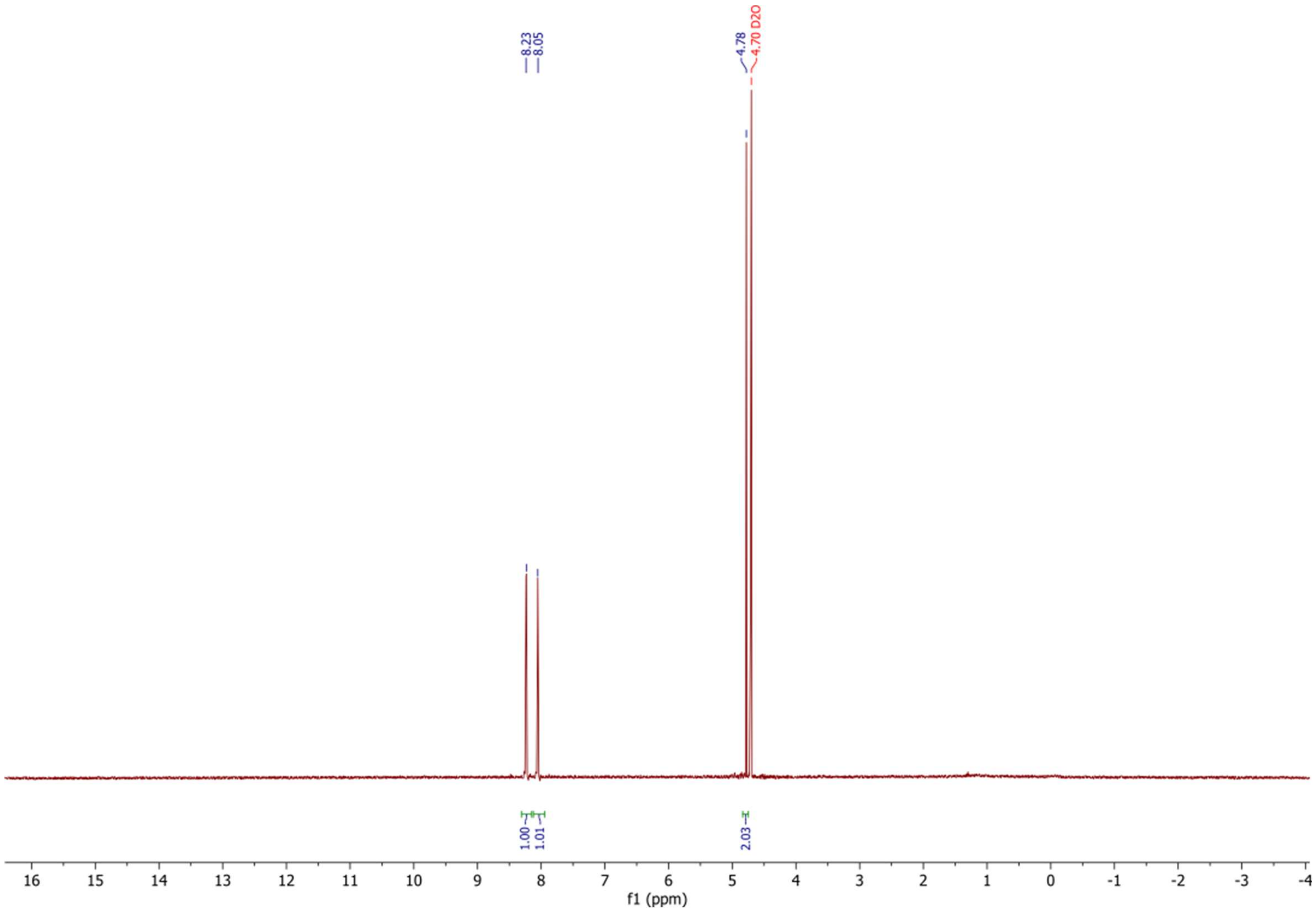

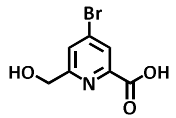

Figure S1. ^{1}H NMR (D_2O, 400 MHz) spectrum of compound **2**.

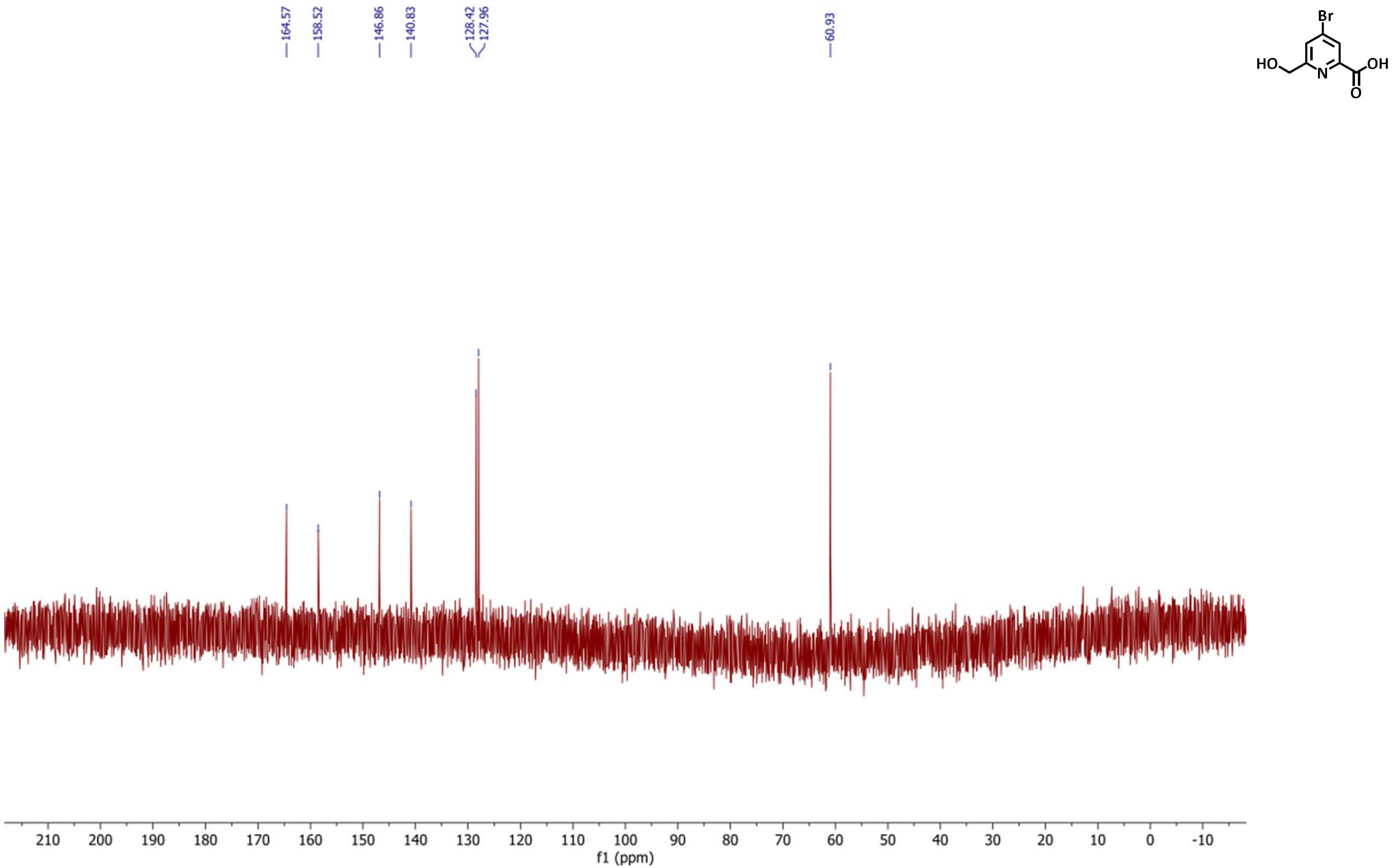

Figure S2. ^{13}C NMR (D_2O, 100 MHz) spectrum of compound **2**.

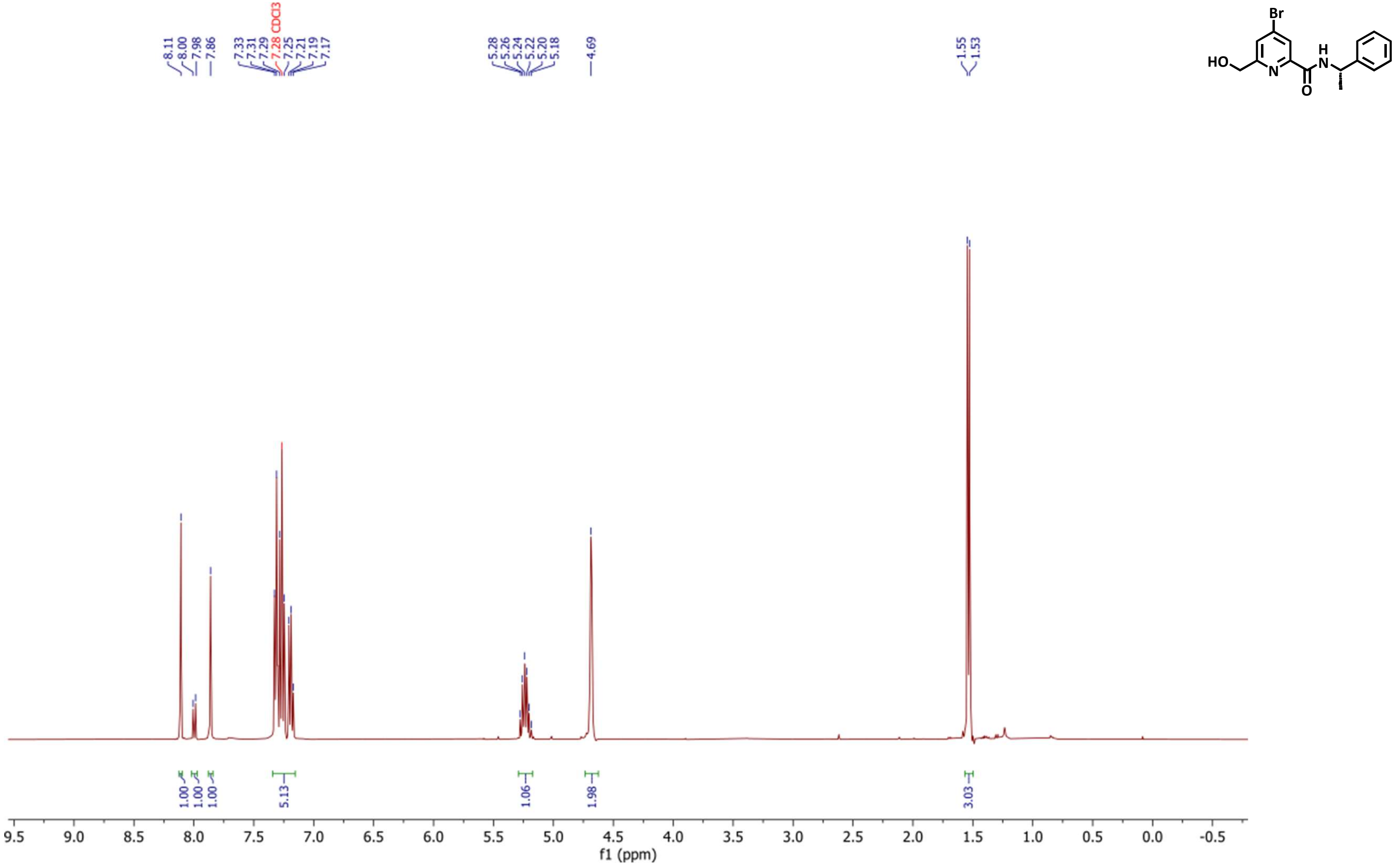

Figure S3. ^{1}H NMR ($CDCl_3$, 400 MHz) spectrum of compound **4**.

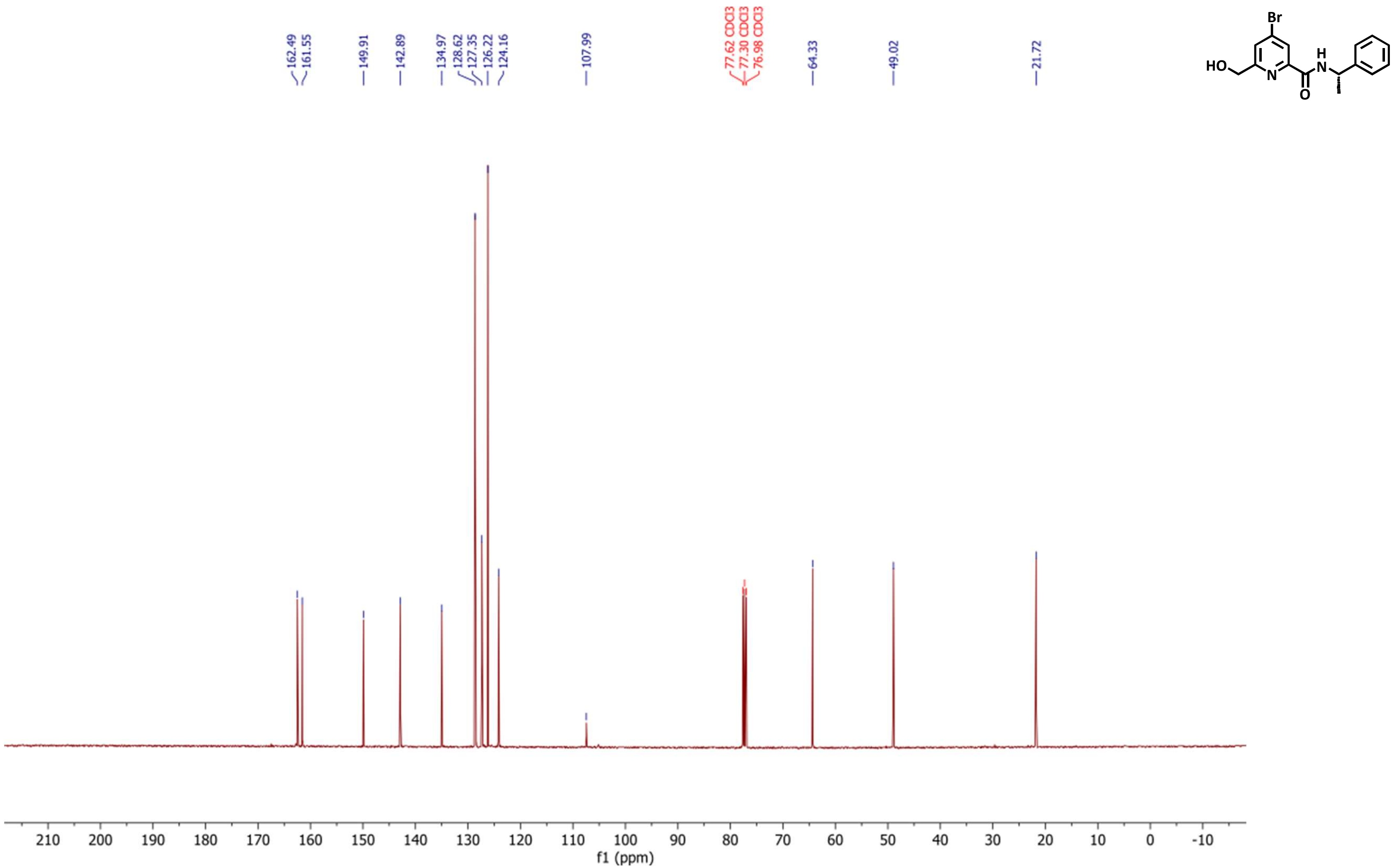

Figure S4. ^{13}C NMR ($CDCl_3$, 100 MHz) spectrum of compound **4**.

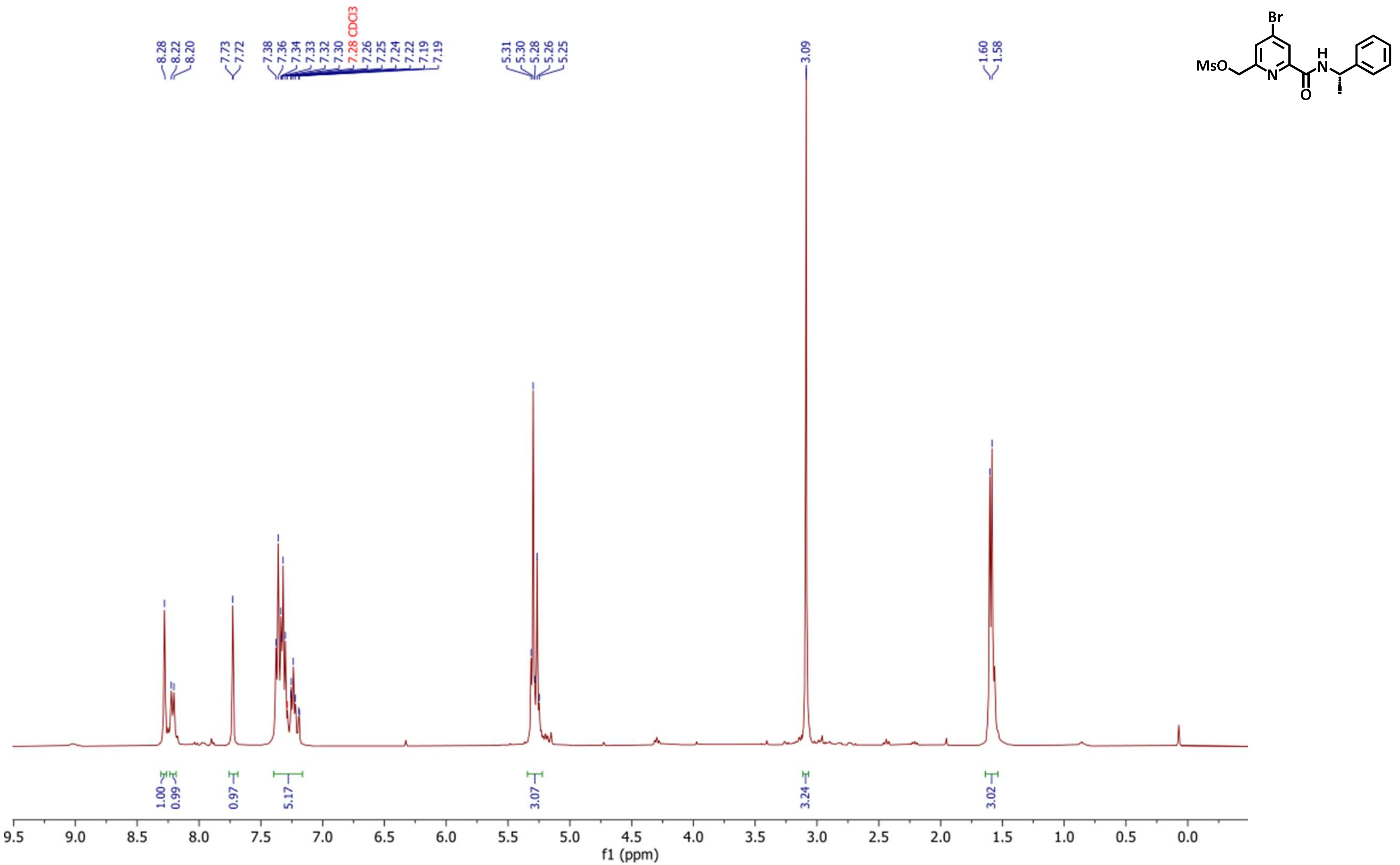

Figure S5. ^{1}H NMR ($CDCl_3$, 400 MHz) spectrum of compound **5**.

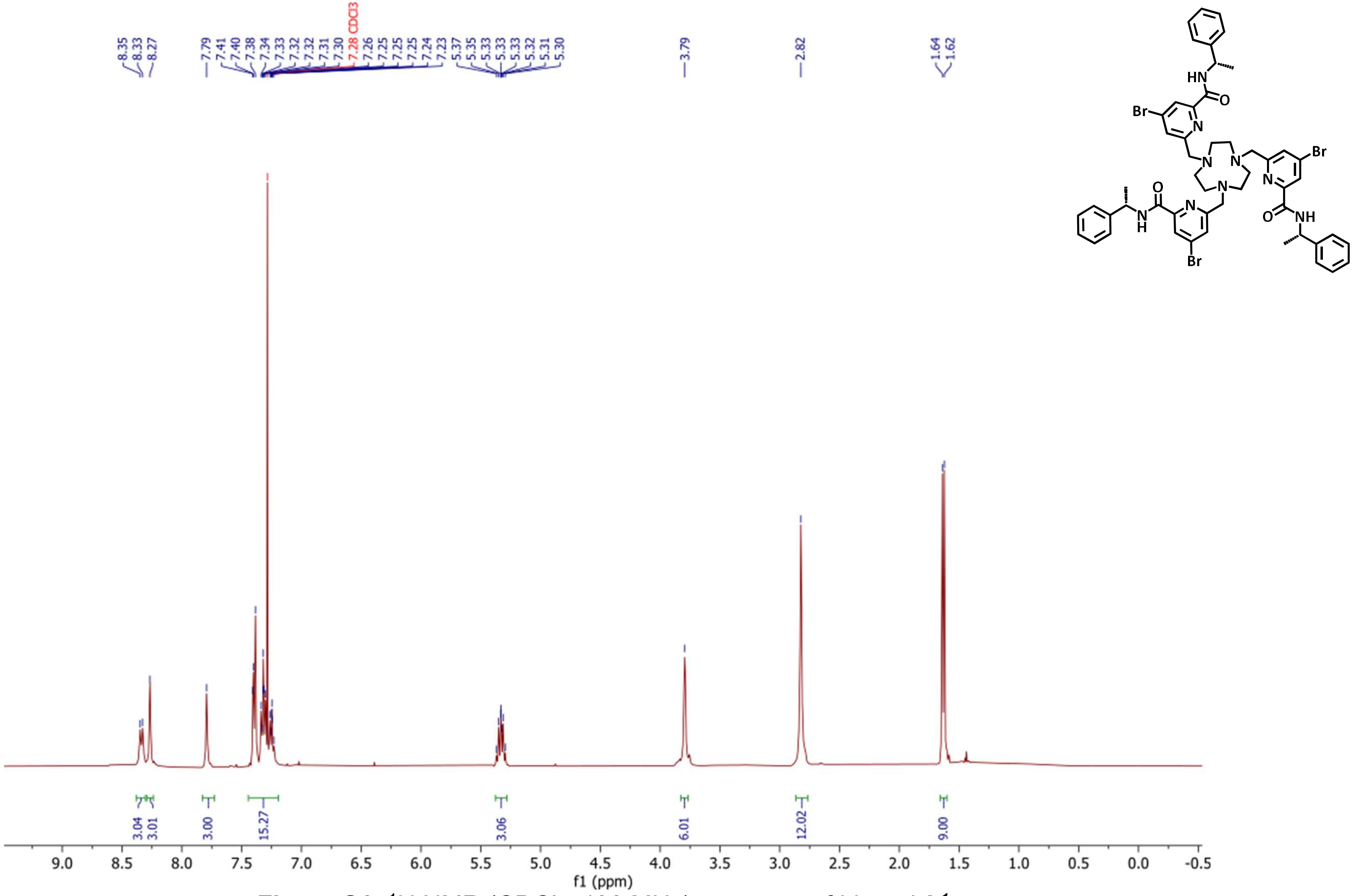

Figure S6. ^{1}H NMR ($CDCl_3$, 400 MHz) spectrum of Ligand ***L^{1a}***

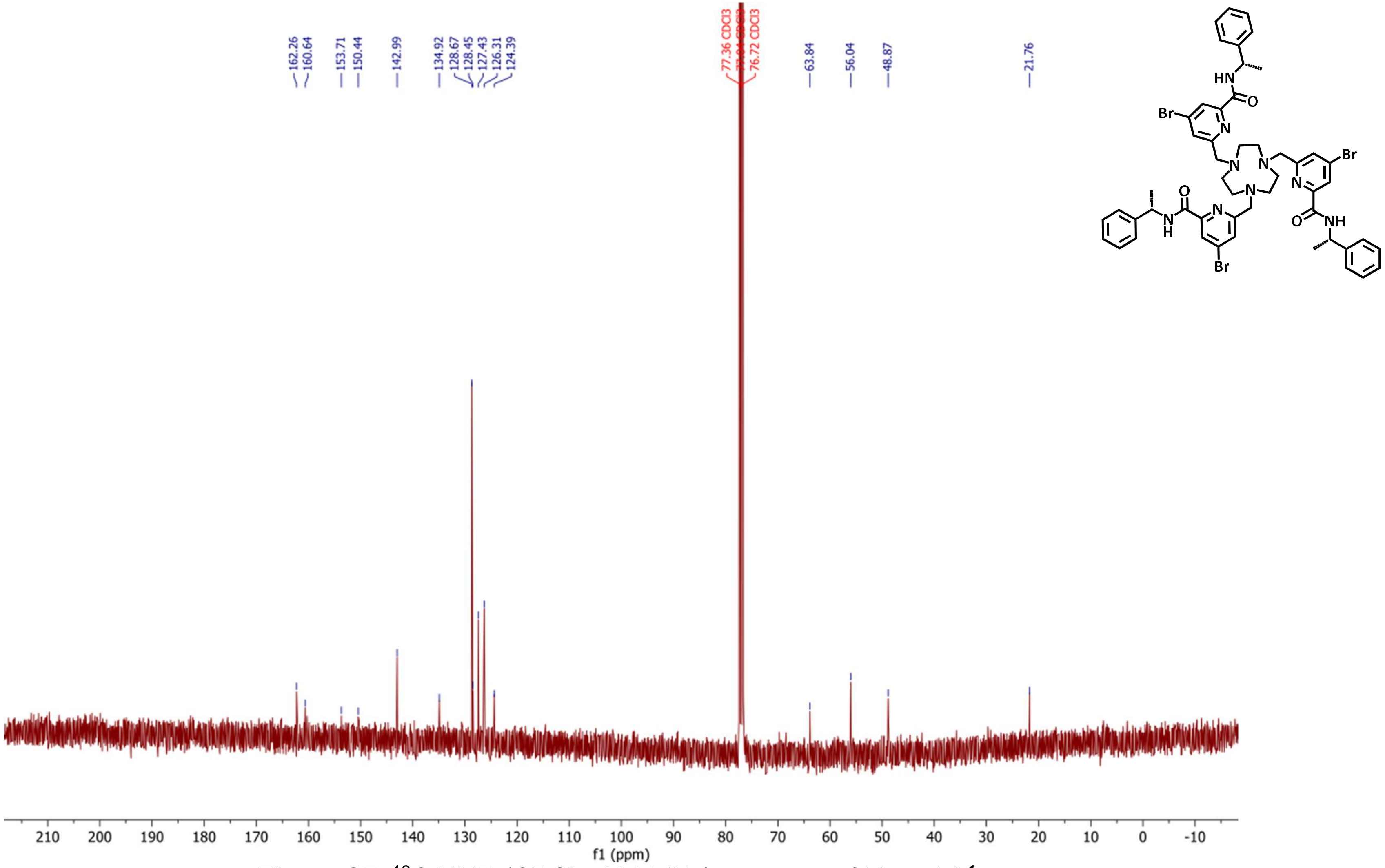

Figure S7. ^{13}C NMR ($CDCl_3$, 100 MHz) spectrum of Ligand ***L^1a***

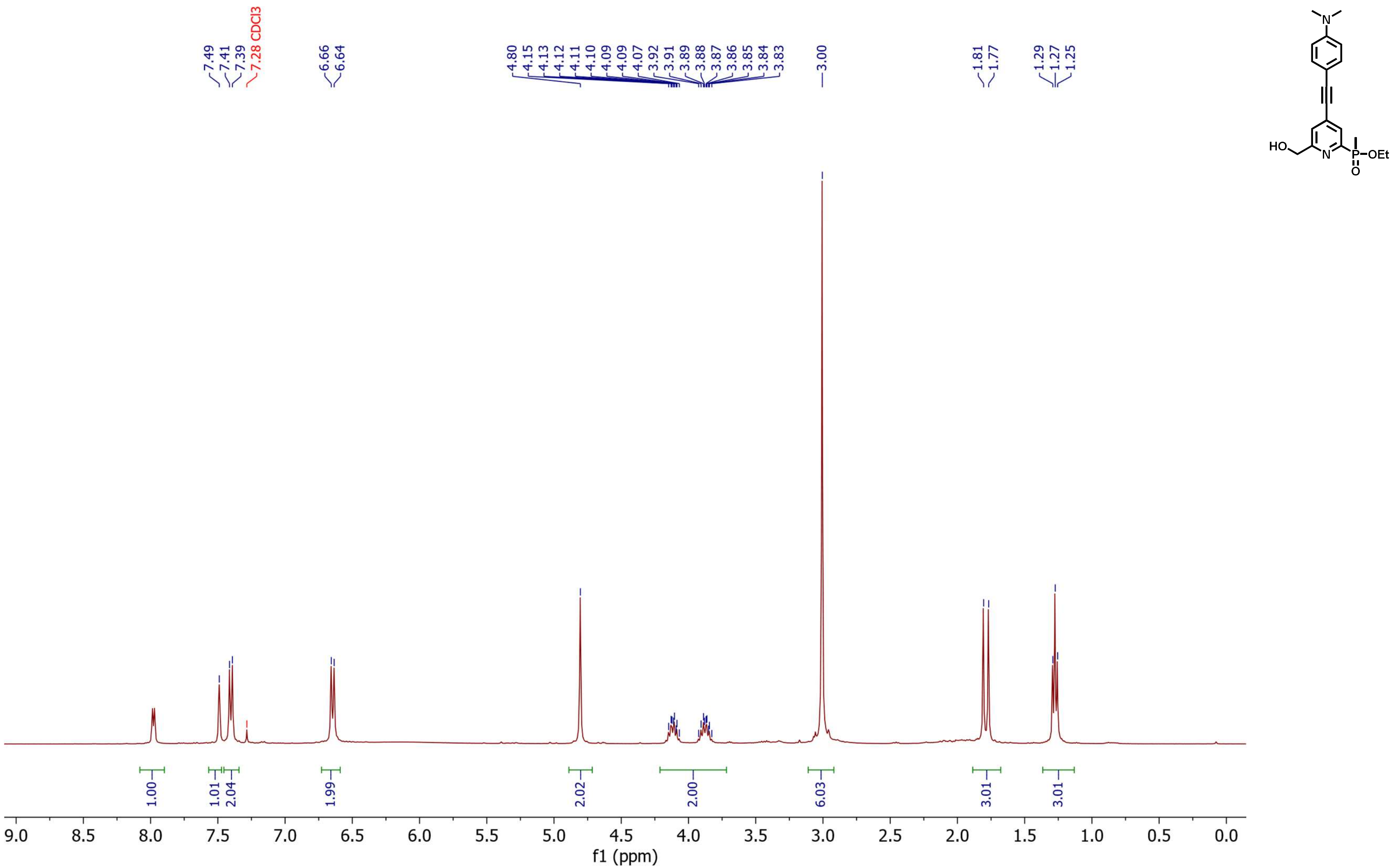

Figure S8. ^{1}H NMR ($CDCl_3$, 400 MHz) spectrum of compound **10**.

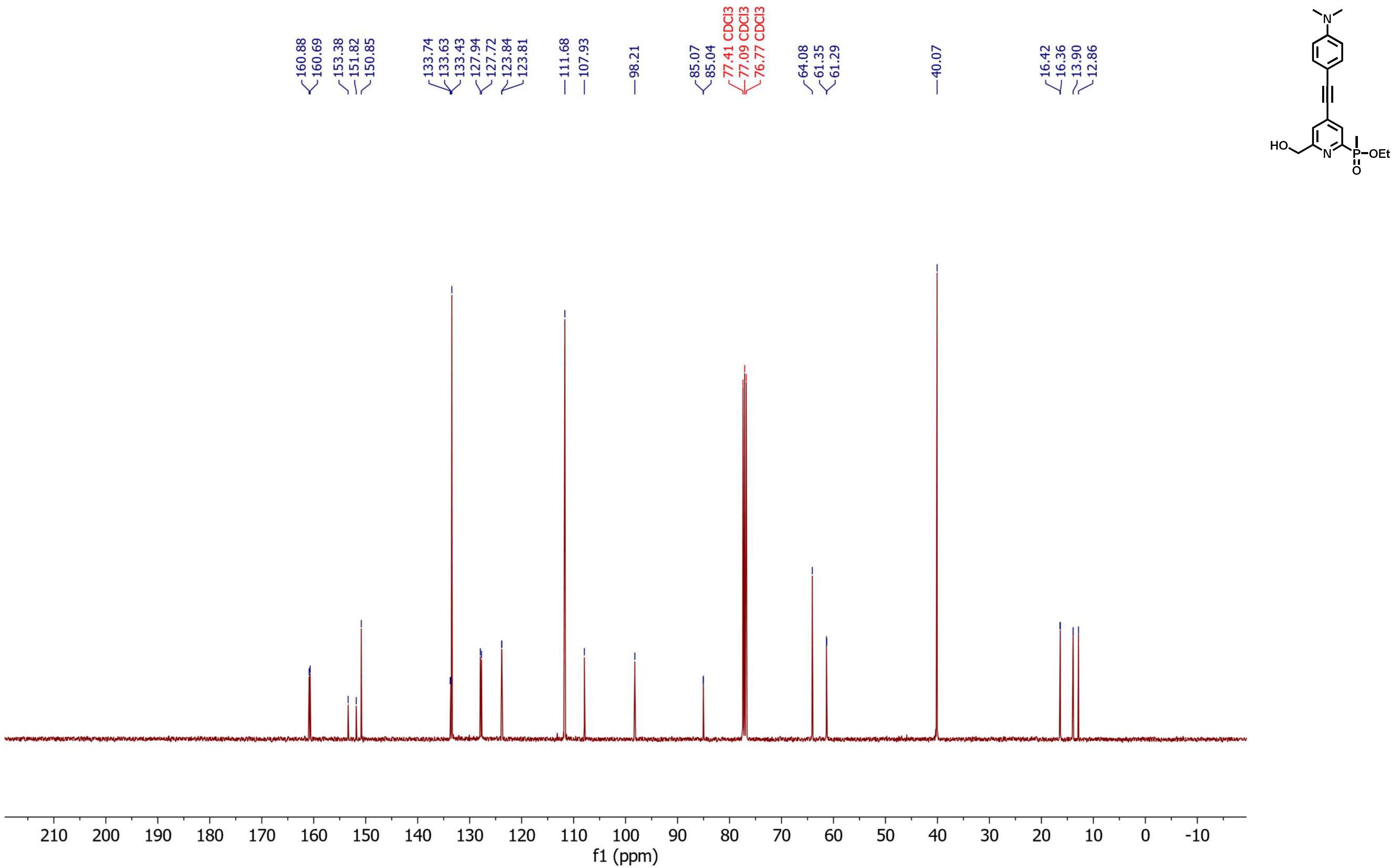

Figure S9. ^{13}C NMR ($CDCl_3$, 100 MHz) spectrum of compound **10**.

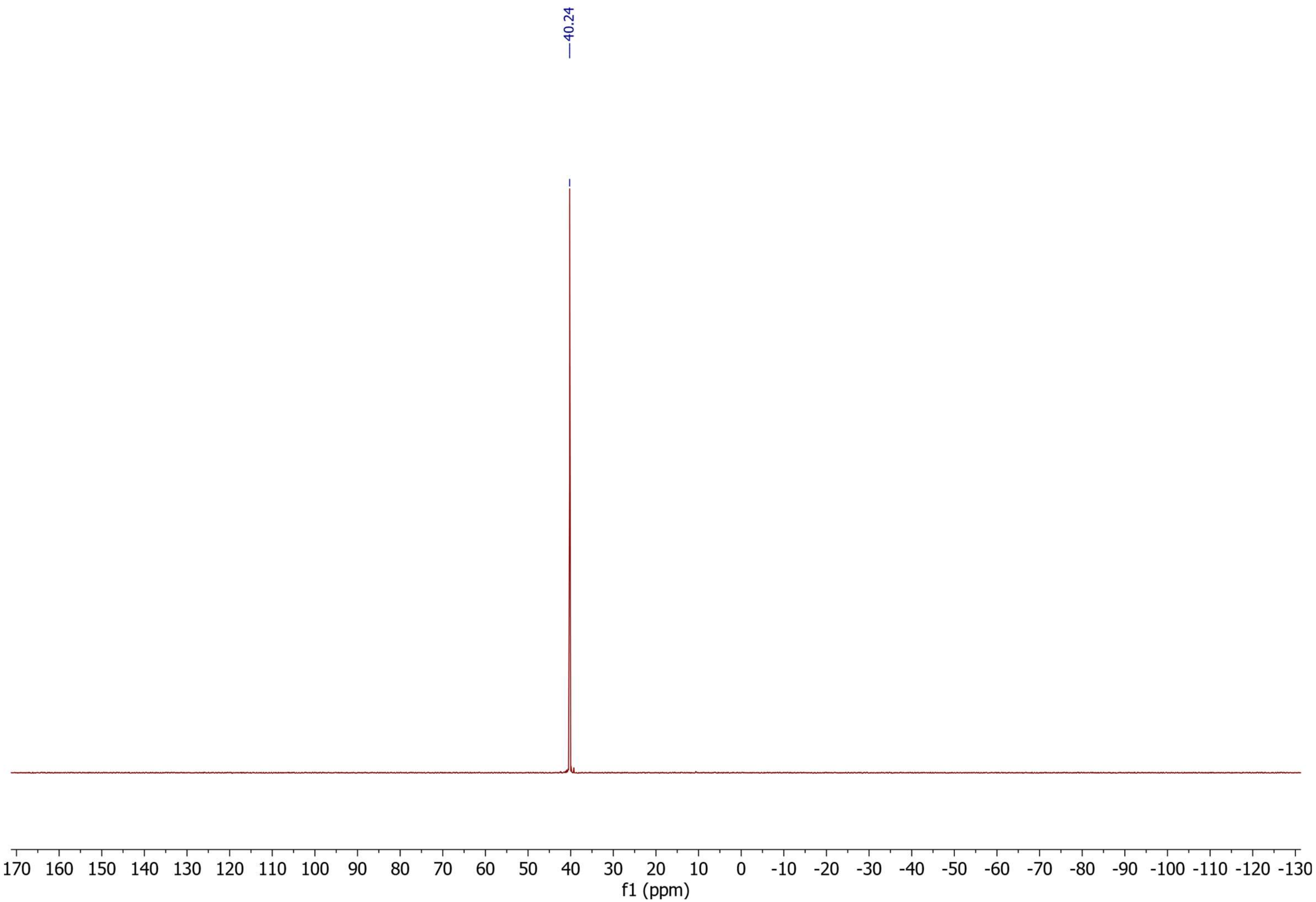

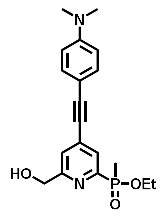

Figure S10. ^{31}P NMR ($CDCl_3$, 162 MHz) spectrum of compound **10.**

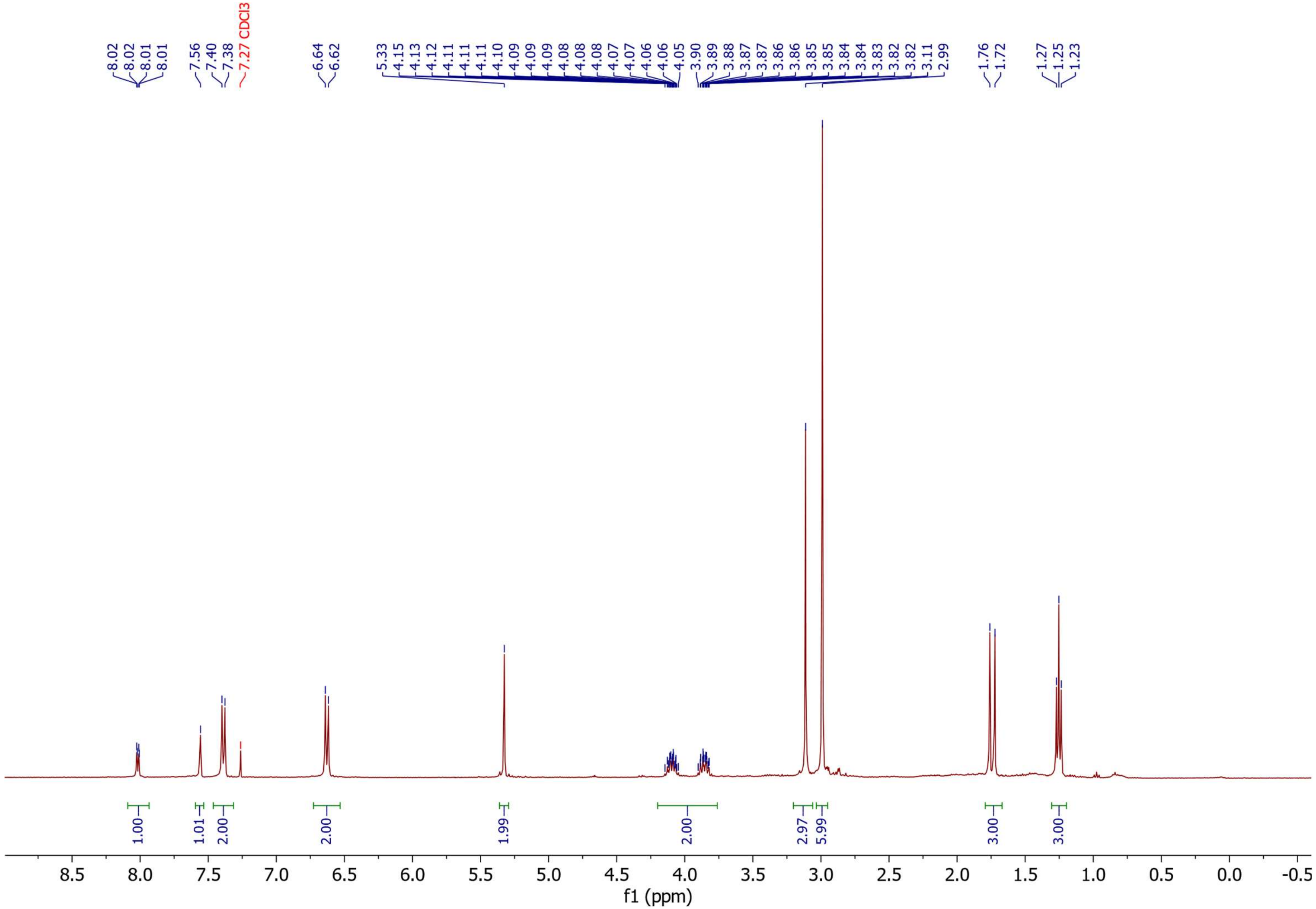

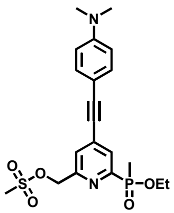

Figure S11. ^{1}H NMR ($CDCl_3$, 400 MHz) spectrum of compound **11.**

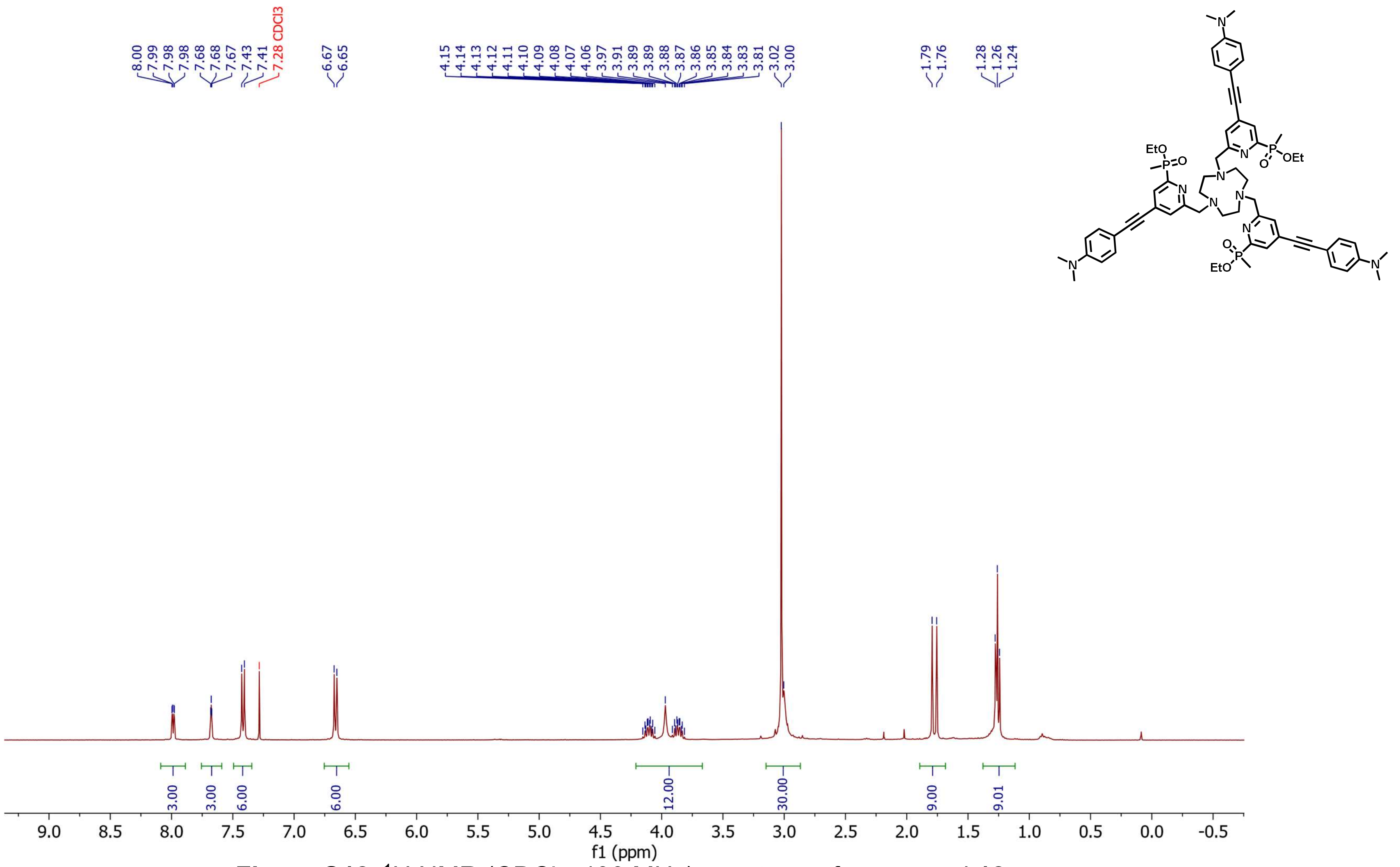

Figure S12. ^1H NMR ($CDCl_3$, 400 MHz) spectrum of compound **12**.

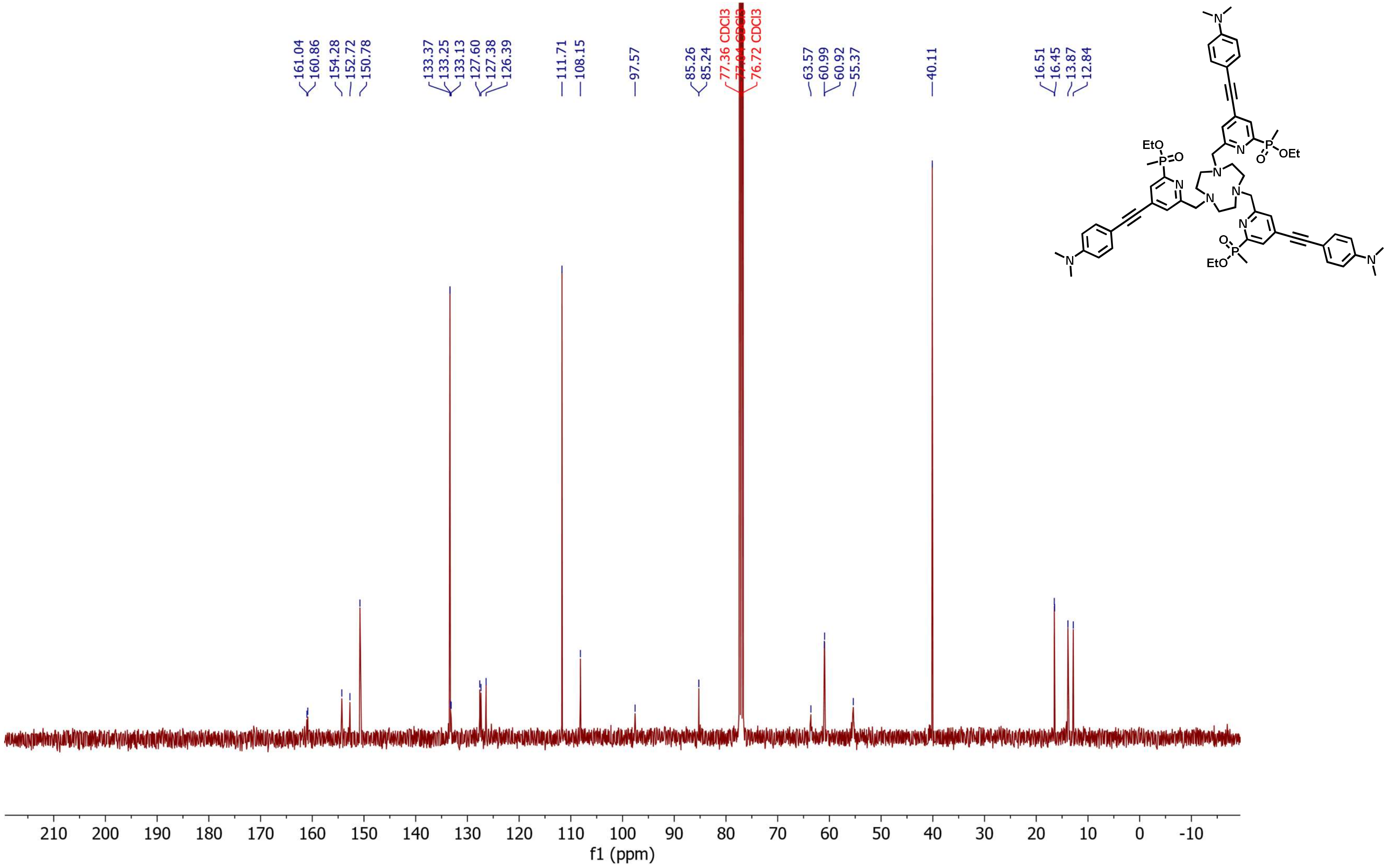

Figure S13. ^{13}C NMR ($CDCl_3$, 100 MHz) spectrum of compound **12**.

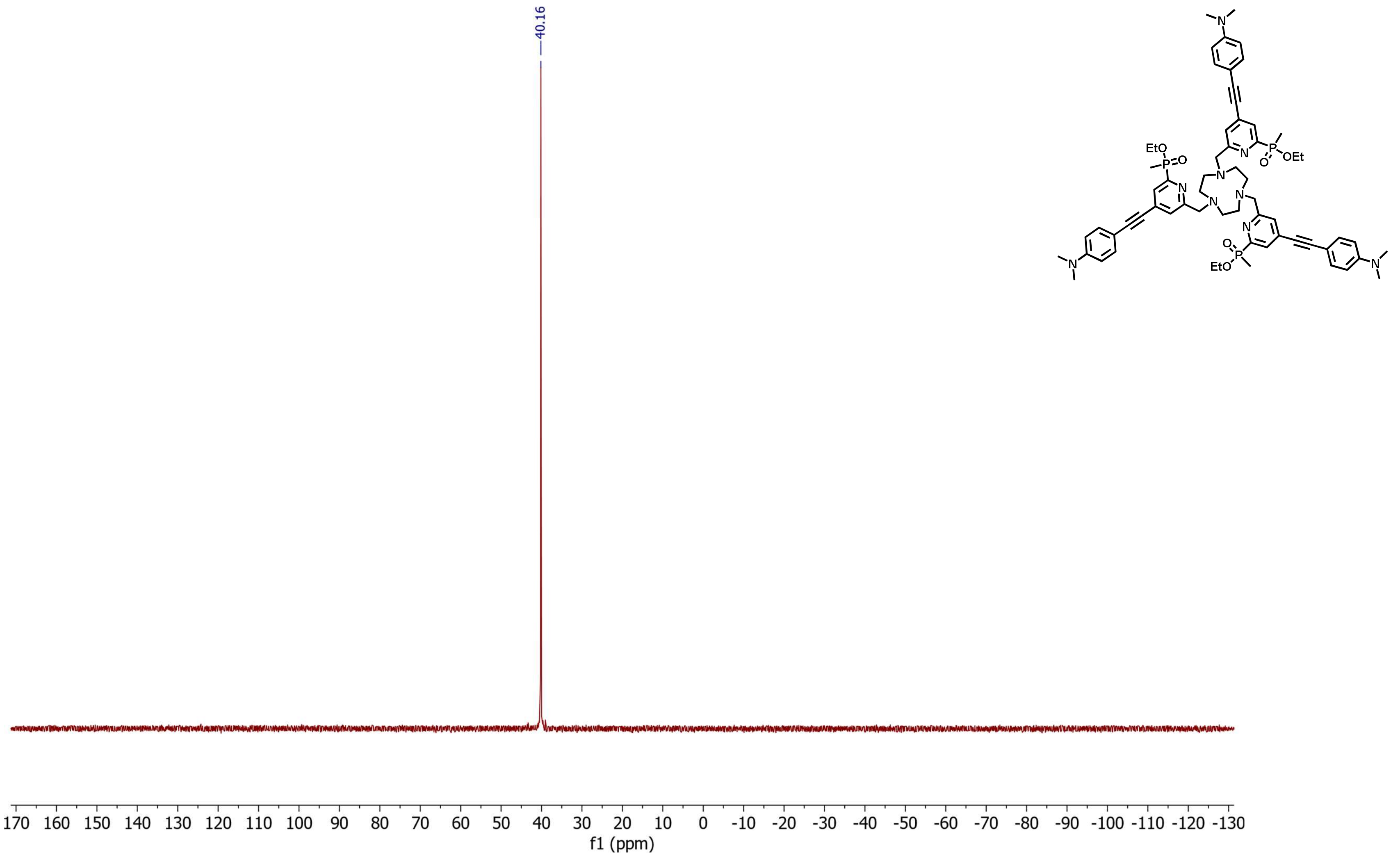

Figure S14. ^{31}P NMR ($CDCl_3$, 162 MHz) spectrum of compound **12**.

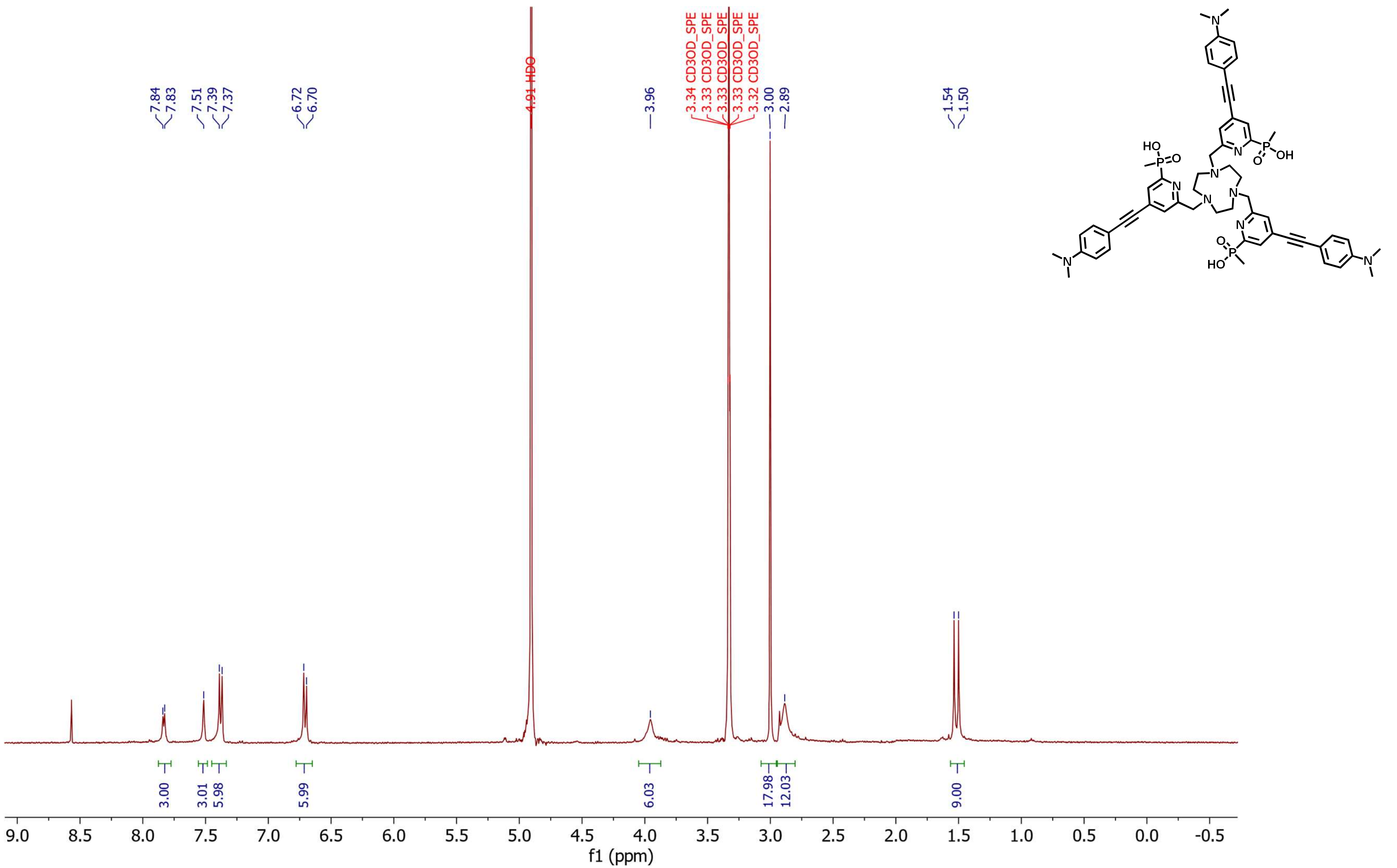

Figure S15. ^{1}H NMR ($CDCl_3$, 400 MHz) spectrum of ligand ***L*^{4}**.

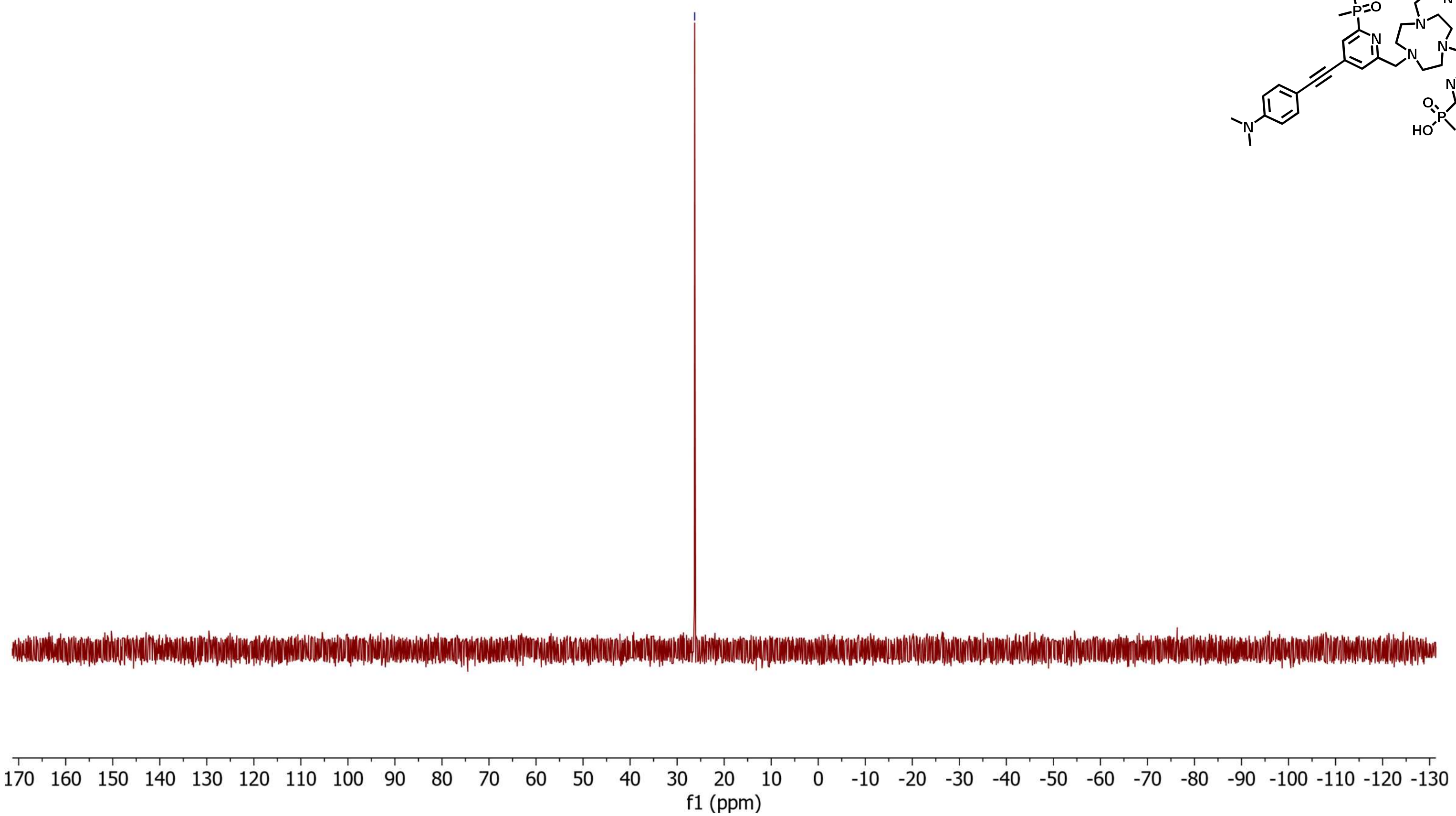

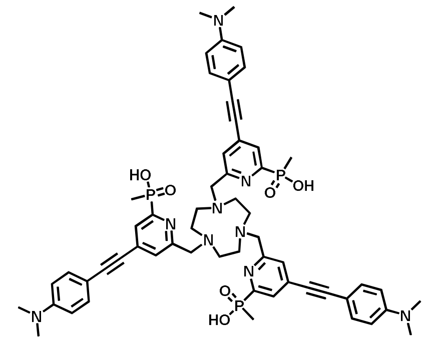

Figure S16. ^{31}P NMR ($CDCl_3$, 162 MHz) spectrum of ligand ***L⁴***.

HPLC Traces

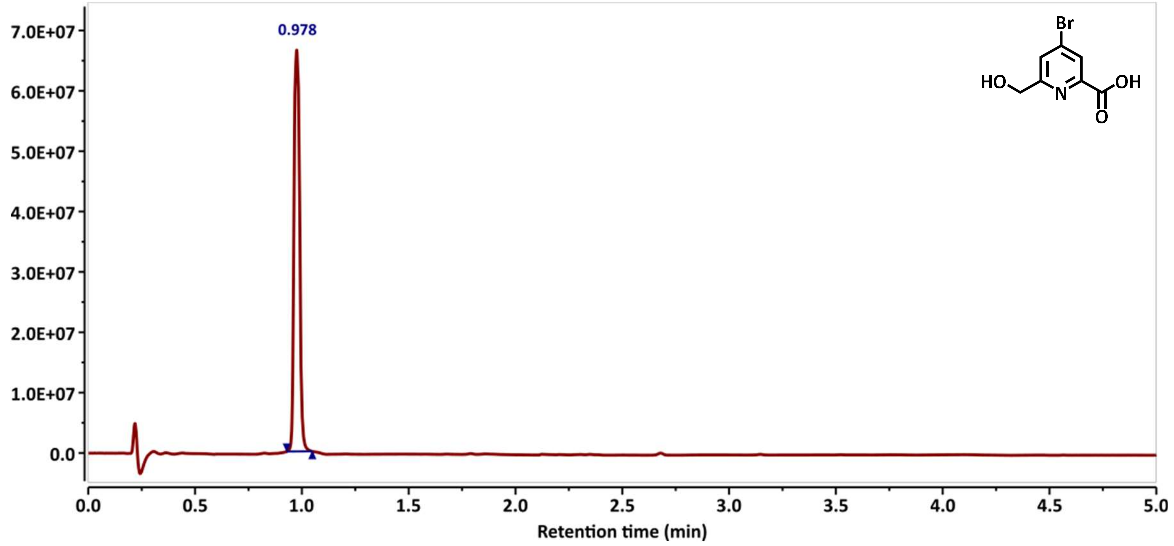

Figure S17. HPLC trace of compound **2.**

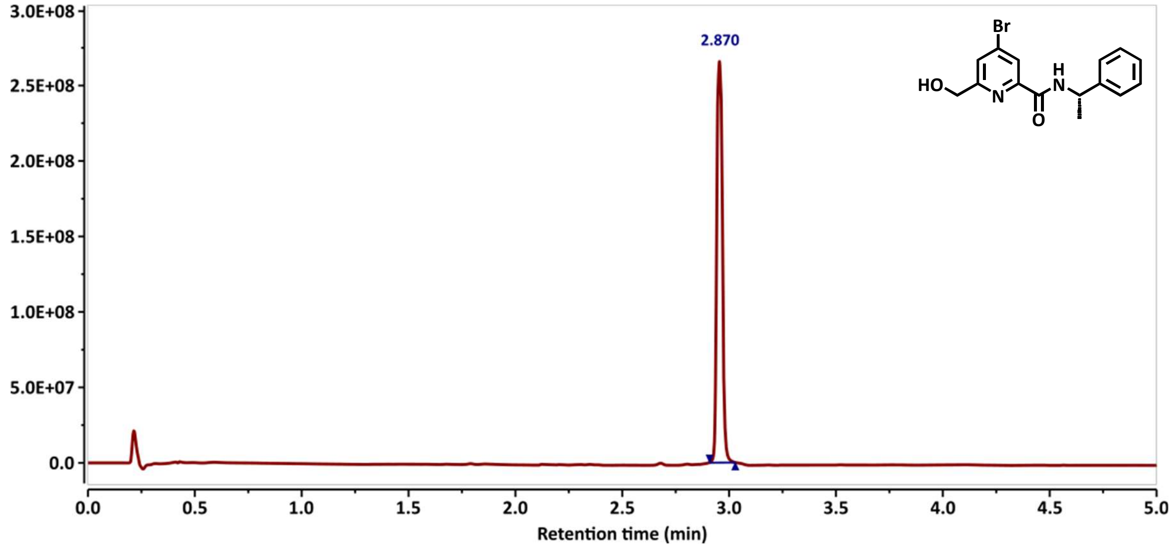

Figure S18. HPLC trace of compound **4.**

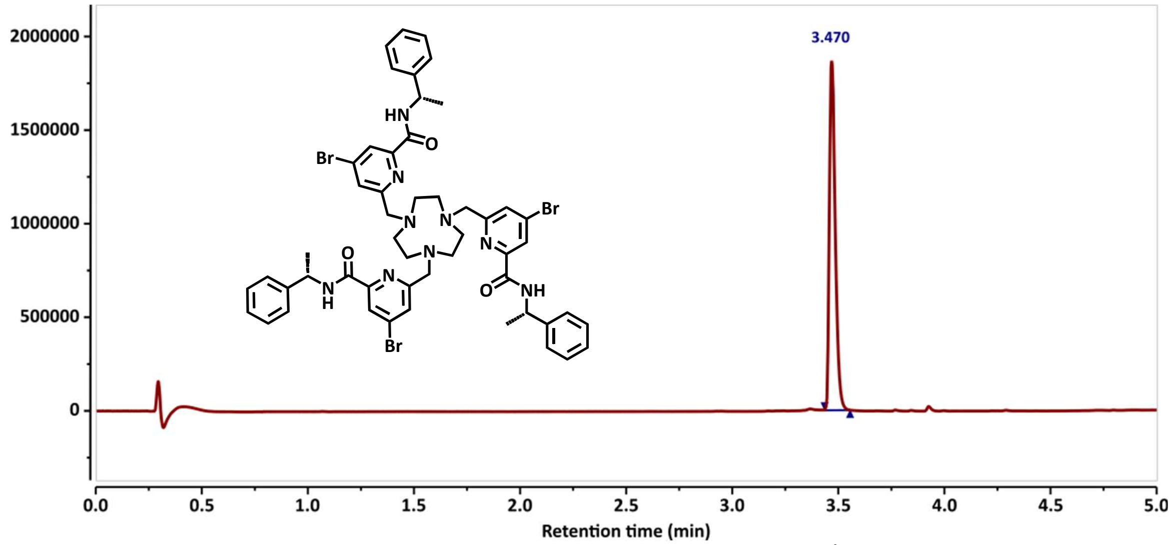

Figure S19. HPLC trace of ligand $\boldsymbol{L^{1a}}$.

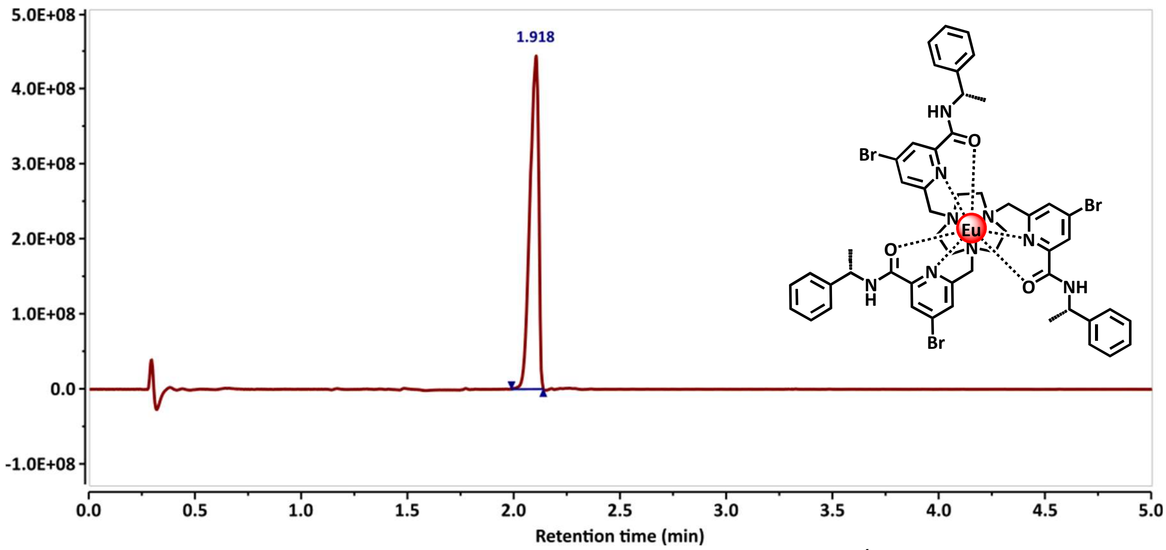

Figure S20. HPLC trace of complex **[Eu*L*[1a]]**.

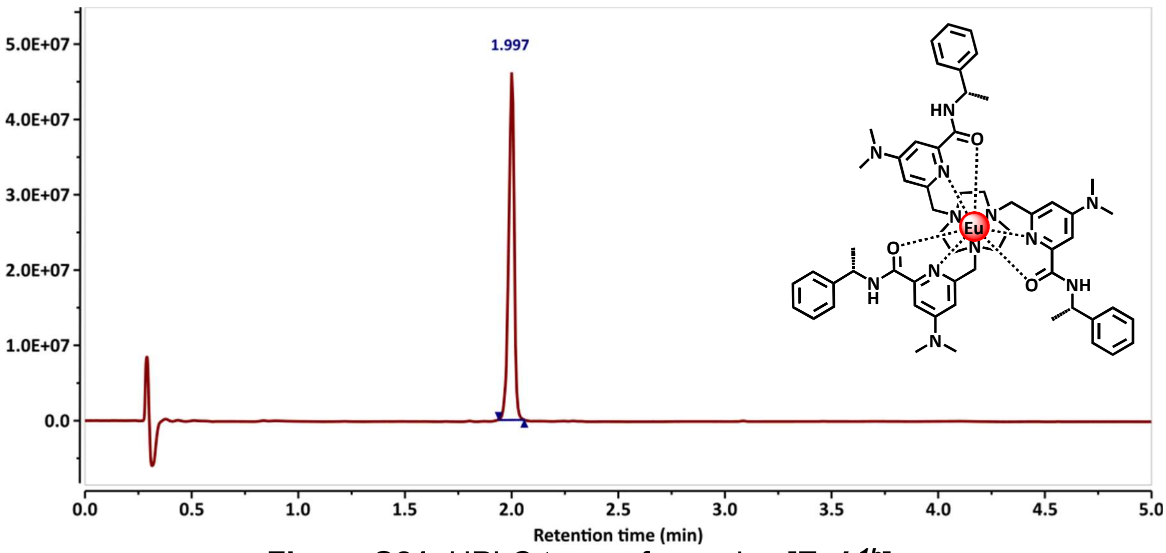

Figure S21. HPLC trace of complex **[Eu*L*[1b]]**.

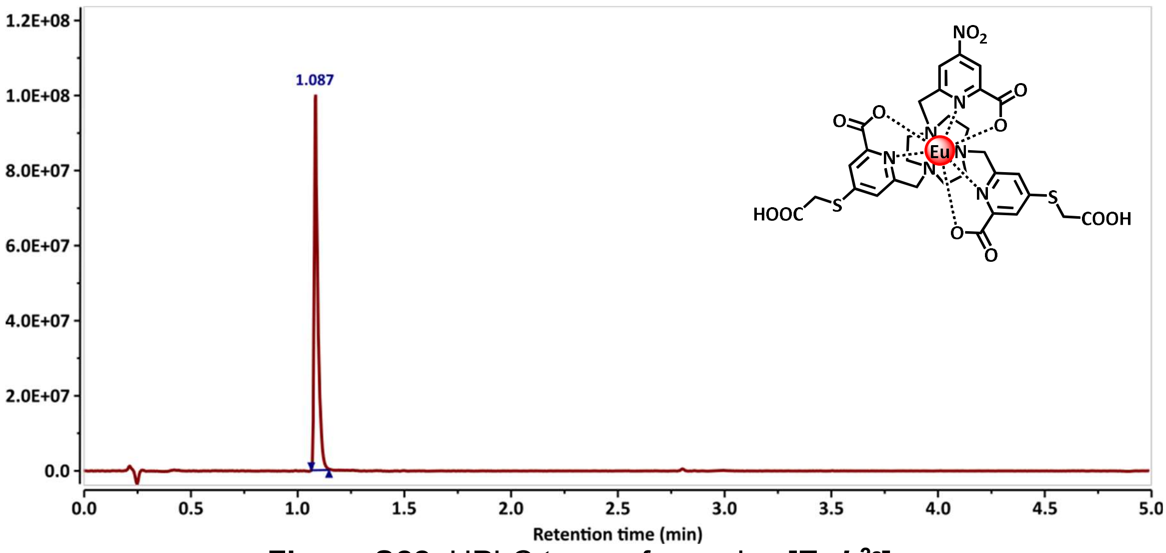

Figure S22. HPLC trace of complex **[Eu*L*[2a]]**.

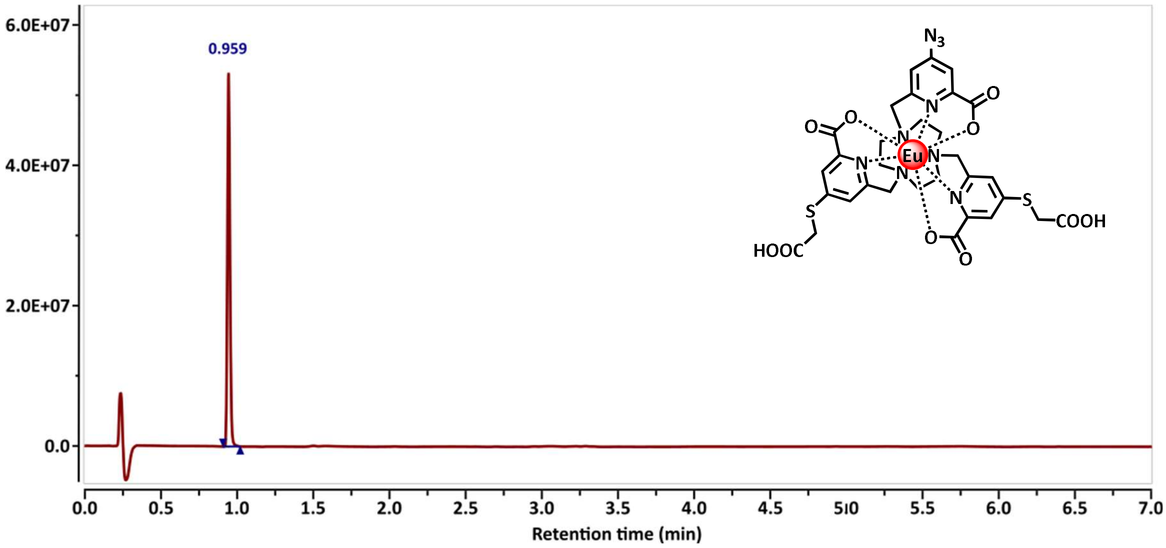

Figure S23. HPLC trace of complex **[Eu*L*[2b]]**.

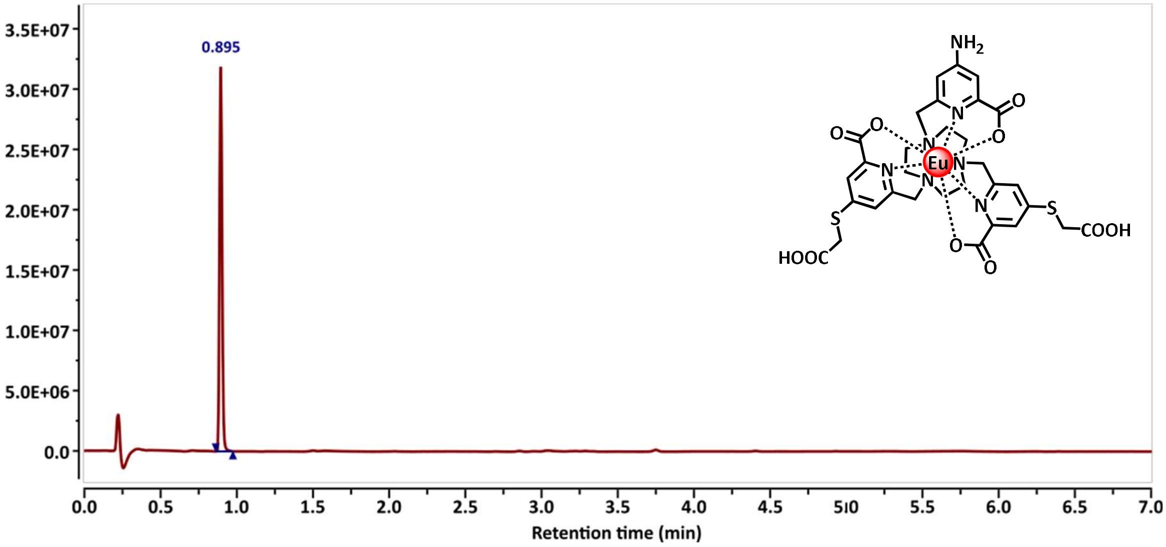

Figure S24. HPLC trace of complex **[Eu*L*[2c]]**.

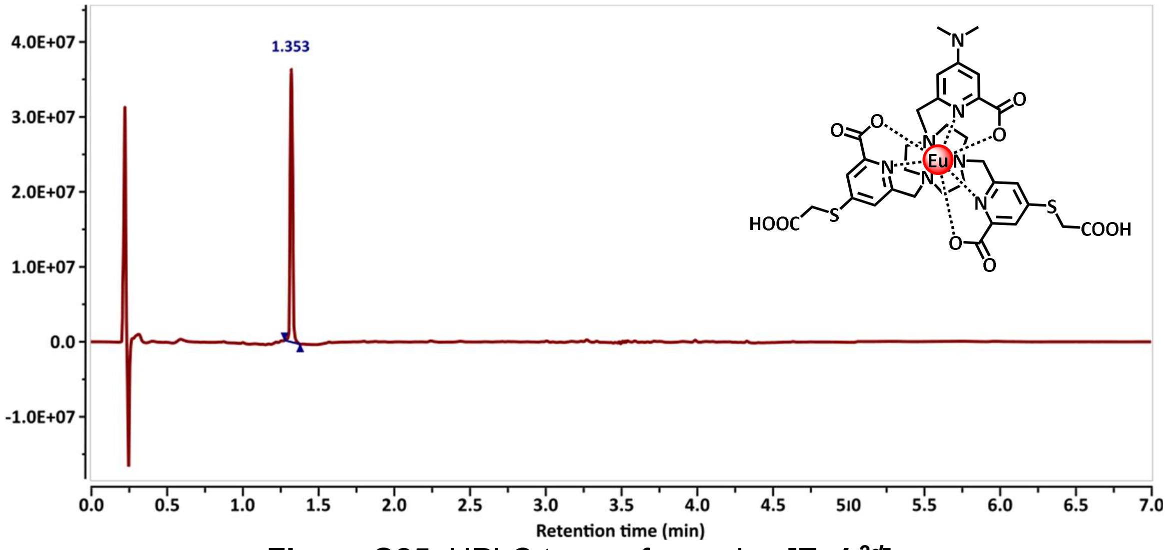

Figure S25. HPLC trace of complex **[Eu*L*[2d]]**.

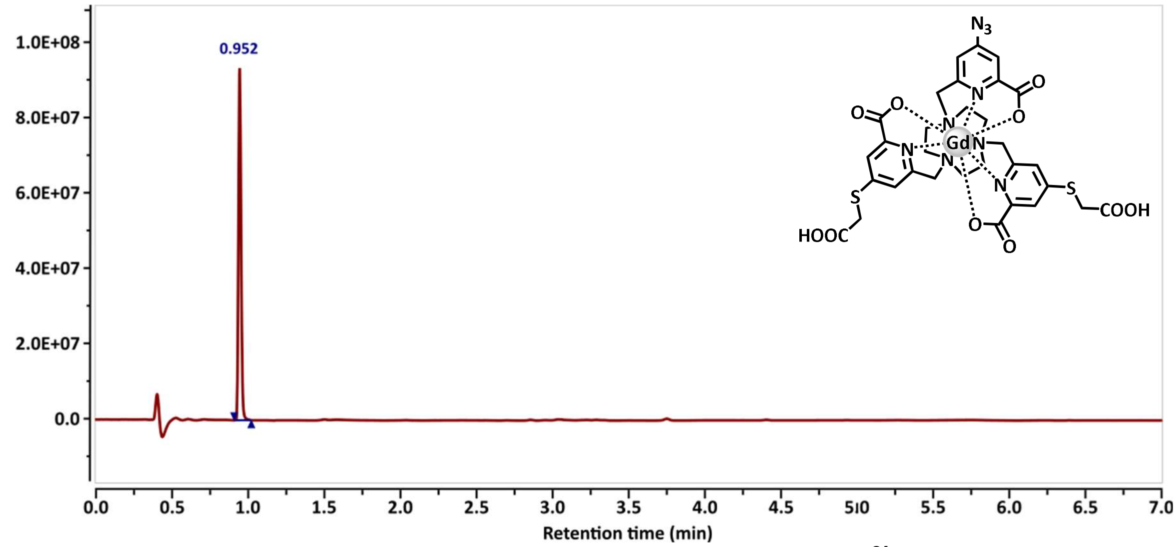

Figure S26. HPLC trace of complex **[Gd*L*^2b^]**.

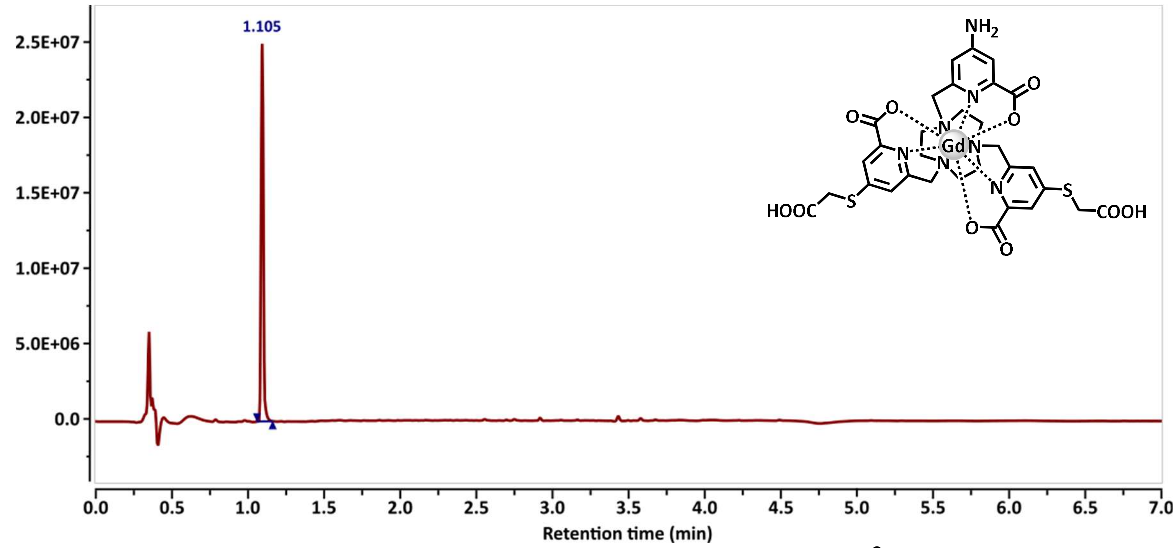

Figure S27. HPLC trace of complex **[Gd*L*^2c^]**.

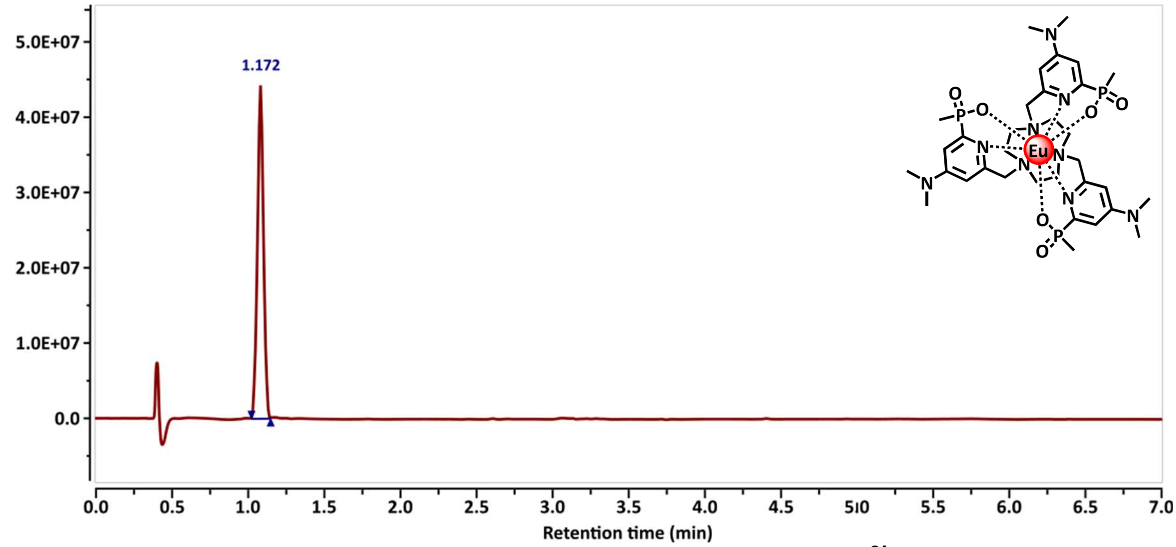

Figure S28. HPLC trace of complex **[Eu*L*^3b^]**.

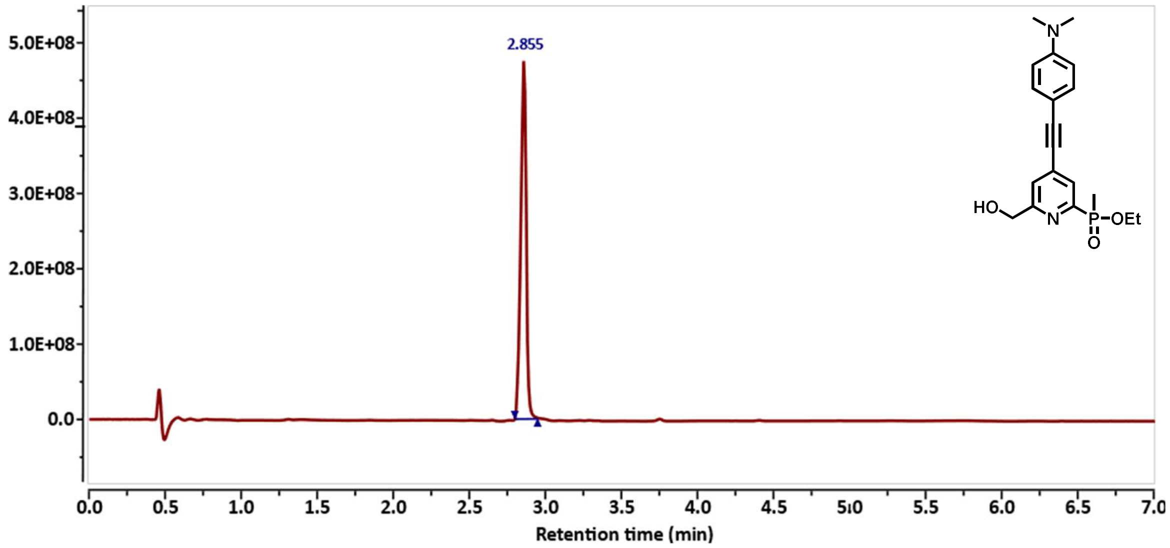

Figure S29. HPLC trace of compound **10**.

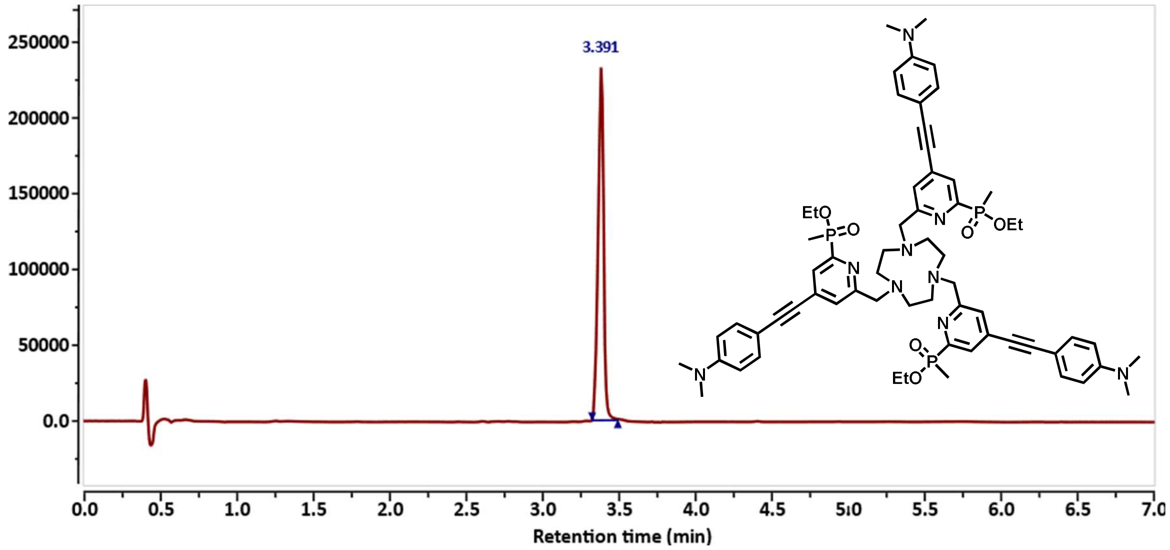

Figure S30. HPLC trace of compound **12**.

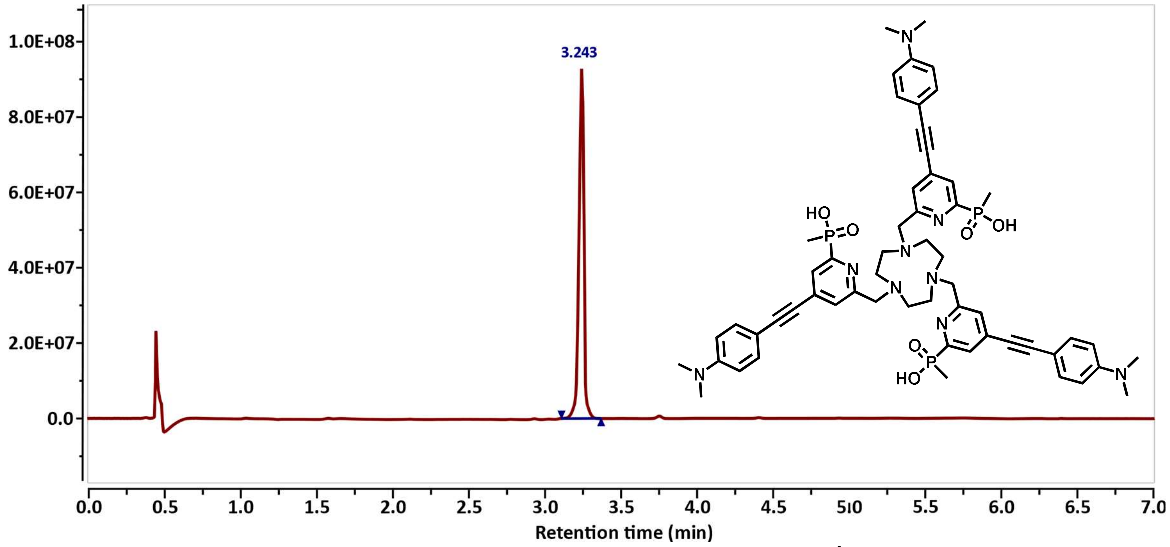

Figure S31. HPLC trace of ligand ***L⁴***.

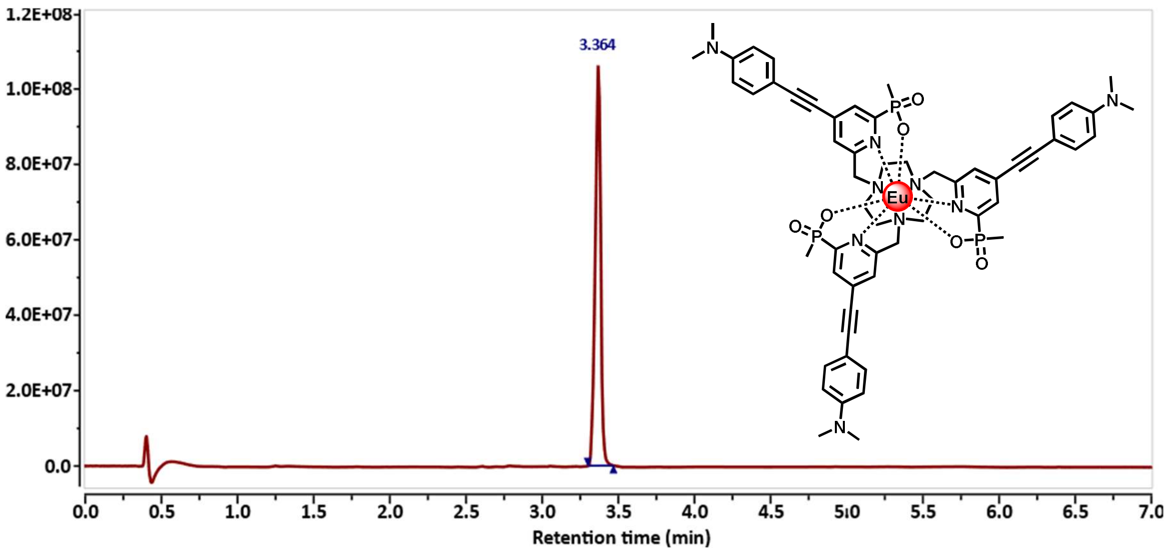

Figure S32. HPLC trace of complex **[Eu*L*[4]]**.

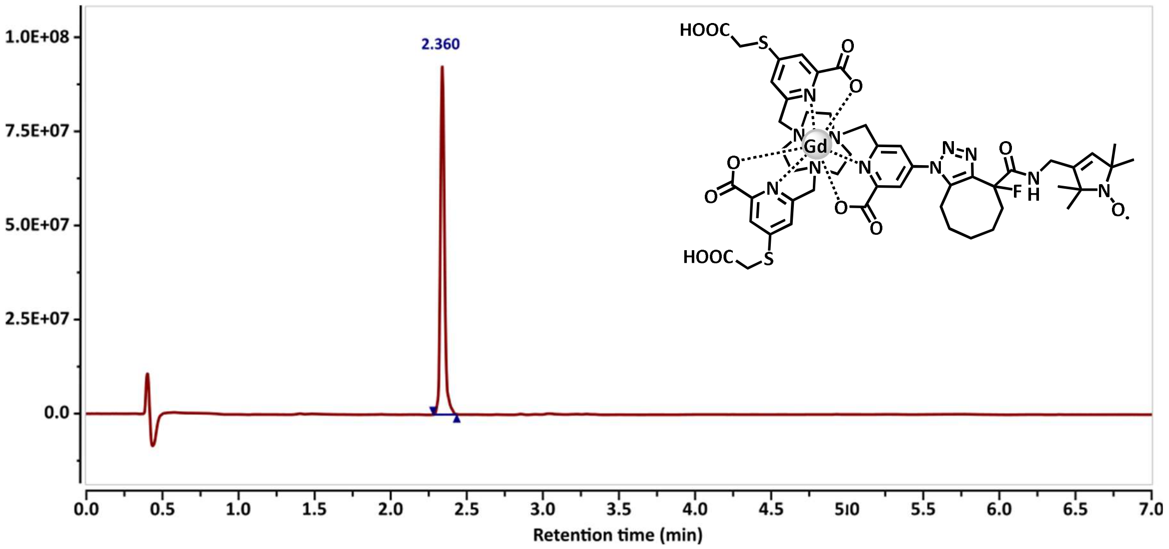

Figure S33. HPLC trace of complex **[Gd*L*[5]]**.

Optical measurements and photophysical data of Eu(III) complexes

Absorption spectroscopy. UV/Vis absorption measurements were recorded using a Perkin-Elmer Lambda 900 absorption spectrophotometer, using matched quartz cells.

Luminescence spectroscopy. Emission spectra were measured using a Horiba-Jobin Yvon Fluorolog-3® spectrofluorimeter. The steady-state luminescence was excited by unpolarised light from a 450W xenon CW lamp and detected at an angle of 90° for diluted solution measurements (10 mm quartz cell) by a red-sensitive Hamamatsu R928 photomultiplier tube. Spectra were reference corrected for both the excitation source light intensity variation (lamp and grating) and the emission spectral response (detector and grating). Phosphorescence lifetimes (> 30 μs) were obtained by pulsed excitation using a FL-1040 UP Xenon Lamp. Luminescence decay curves were fitted by least-squares analysis using Origin®. Luminescence quantum yields *Q* were measured in diluted aqueous solution with an absorbance lower than 0.1 using the following equation (1):

$$\frac{Q_{\mathrm{x}}}{Q_{\mathrm{r}}} = \left[\frac{A_{\mathrm{r}}(\lambda)}{A_{\mathrm{x}}(\lambda)}\right]\left[\frac{n_{\mathrm{x}}^2}{n_{\mathrm{r}}^2}\right]\left[\frac{D_{\mathrm{x}}}{D_{\mathrm{r}}}\right]\left[\frac{I_{\mathrm{lamp}}(\lambda)_{\mathrm{r}}}{I_{\mathrm{lamp}}(\lambda)_{\mathrm{x}}}\right] \quad (1)$$

where *A* is the absorbance at the excitation wavelength (λ), *n* the refractive index, *D* the integrated luminescence intensity, I_{lamp} the energy intensity contribution of the excitation lamp at the excitation wavelength (λ). “r” and “x” stand for reference and sample. Here, the reference is $[Ru(bipy)_3]Cl_2$ in non-degassed water (Φ=2.8%).[6] Estimated errors are ±15%. Spectrometric titrations were performed according to methods described in the literature.[7]

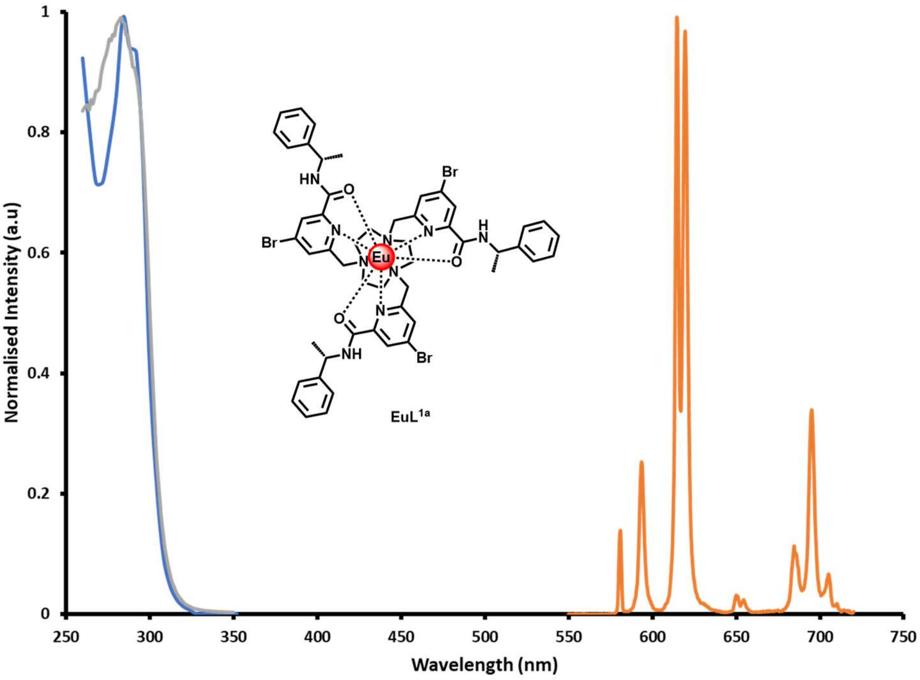

Figure S34. The normalised absorption (*blue*), excitation (*grey* λ_{em} *= 615 nm*), and emission (*orange,* λ_{exc} = 280 nm) spectra for [Eu***L***^{1a}]$^{3+}$ (295 K, MeOH).

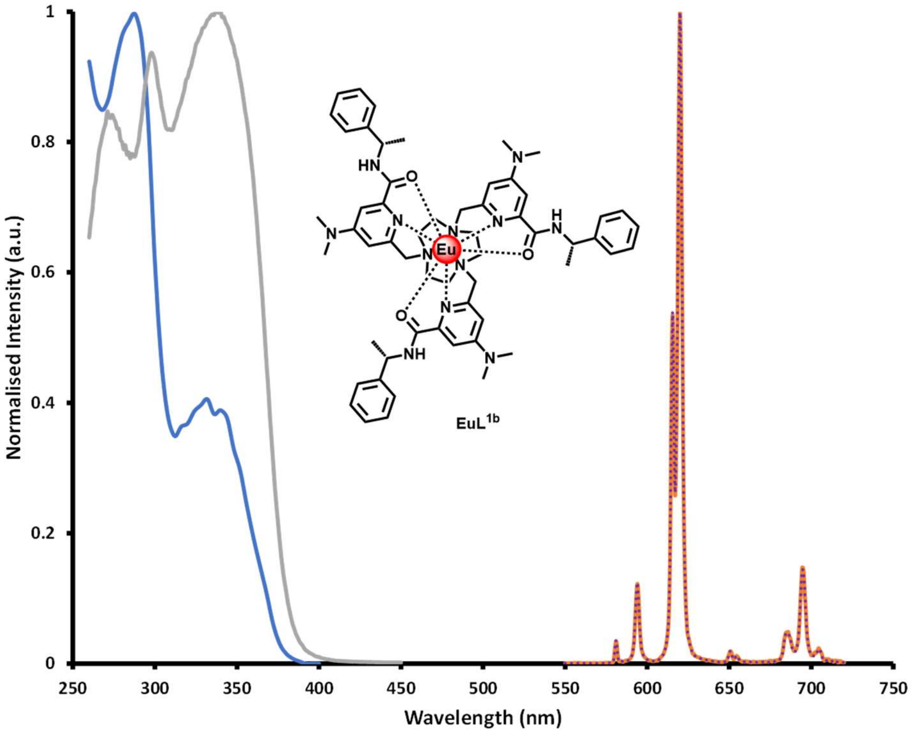

Figure S35. The normalised absorption (*blue*), excitation (*grey* λ_{em} *= 615 nm*) and emission (*orange,* λ_{exc} = 290 nm; *purple dots,* λ_{exc} = 340 nm) spectra for [Eu***L***^{1b}]$^{3+}$ (MeOH, 295 K).

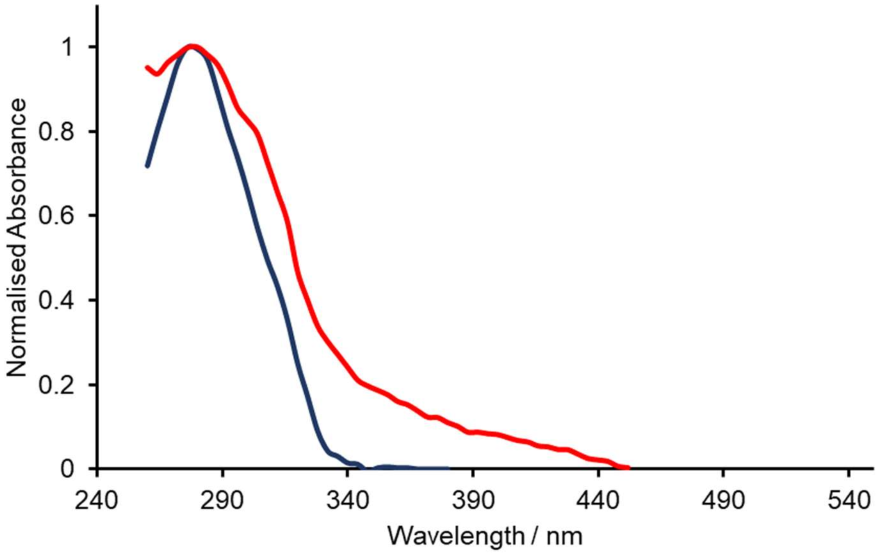

Figure S36. Comparison of the absorption spectra of [Eu***L***^{2b}] and [Eu***L***^{2c}] (*blue* and *red,* respectively, H_2O, 295 K).

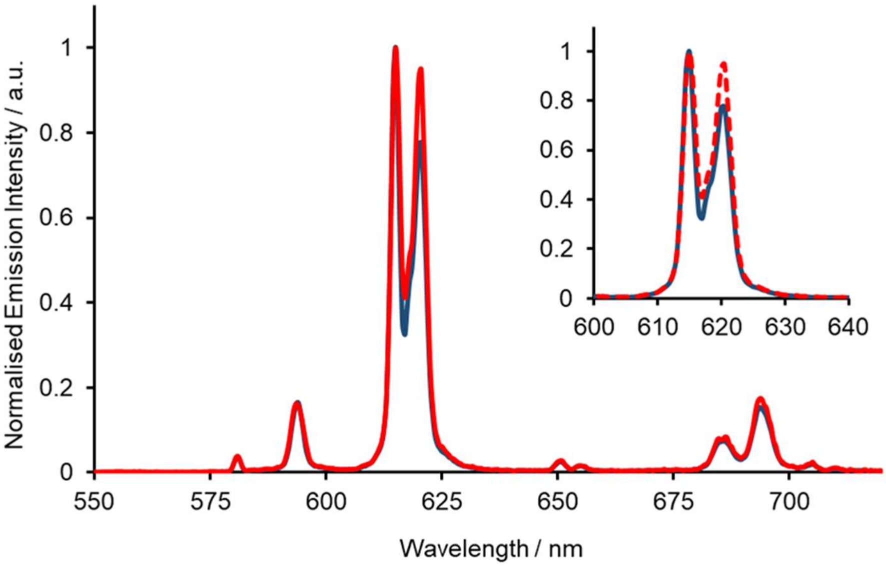

Figure S37. Comparison of the emission spectra of [Eu***L***^{2b}] and [Eu***L***^{2c}] (*blue* and *red,* respectively, H_2O, 295 K). An enlarged view of the ΔJ = 2 band is shown (inset).

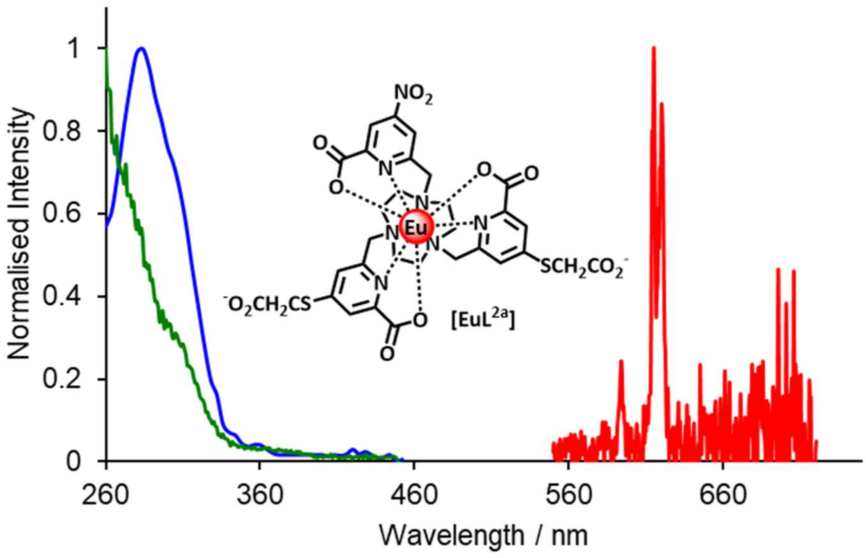

Figure S38. The normalised absorption (*blue*), excitation (*green*, λ_{em} 615 nm), and emission (*red*, λ_{exc} 284 nm) spectra, respectively, for [Eu$\boldsymbol{L^{2a}}$] (H_2O, 295 K).

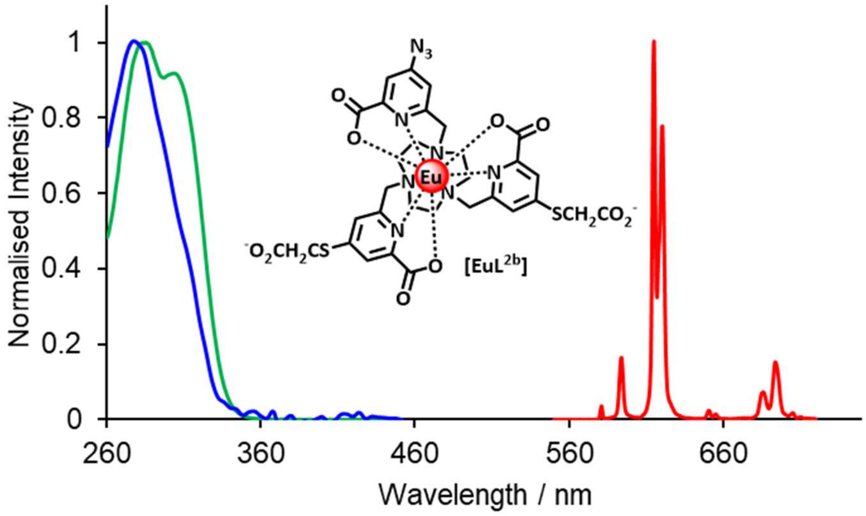

Figure S39. The normalised absorption (*blue*), excitation (*green*, λ_{em} 615 nm), and emission (*red*, λ_{exc} 276 nm) spectra, respectively, for [Eu$\boldsymbol{L^{2b}}$] (H_2O, 295 K).

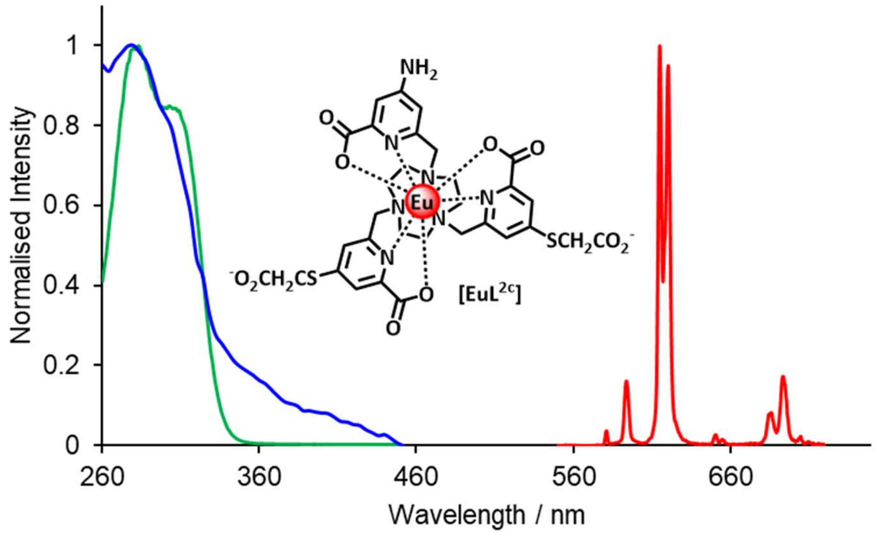

Figure S40. The normalised absorption (*blue*), excitation (*green*, λ_{em} 615 nm), and emission (*red*, λ_{exc} 280 nm) spectra, respectively, for [Eu$\boldsymbol{L^{2c}}$] (H_2O, 295 K).

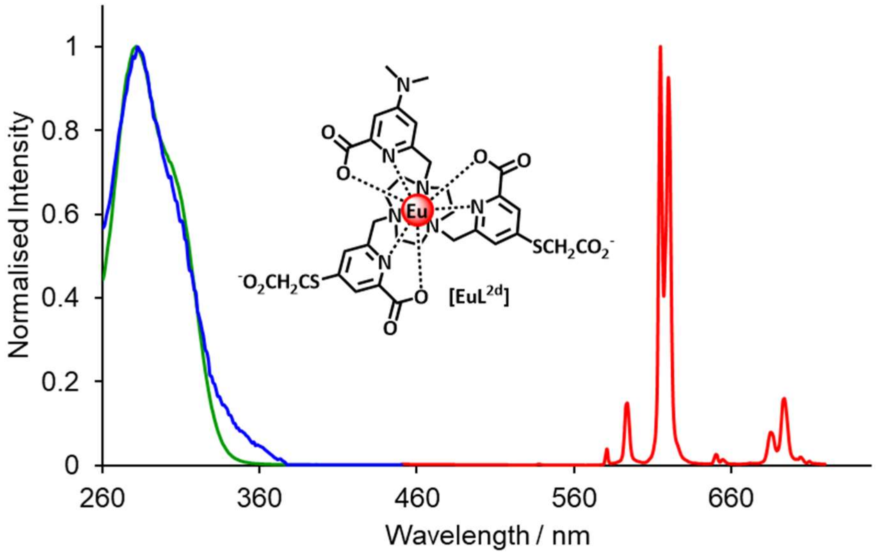

Figure S41. The normalised absorption (*blue*), excitation (*green*, λ_{em} 615 nm), and emission (*red*, λ_{exc} 282 nm) spectra, respectively, for [Eu$\boldsymbol{L^{2d}}$] (H_2O, 295 K).

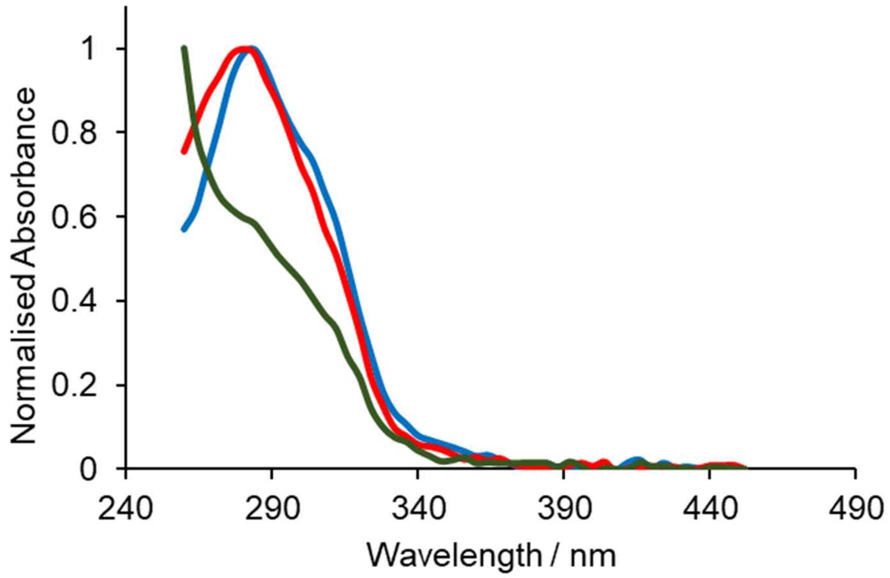

Figure S42. Comparison of the absorption spectra of [Gd$\boldsymbol{L}^{2a-c}$] (*blue, red,* and *green* respectively, H_2O, 295 K).

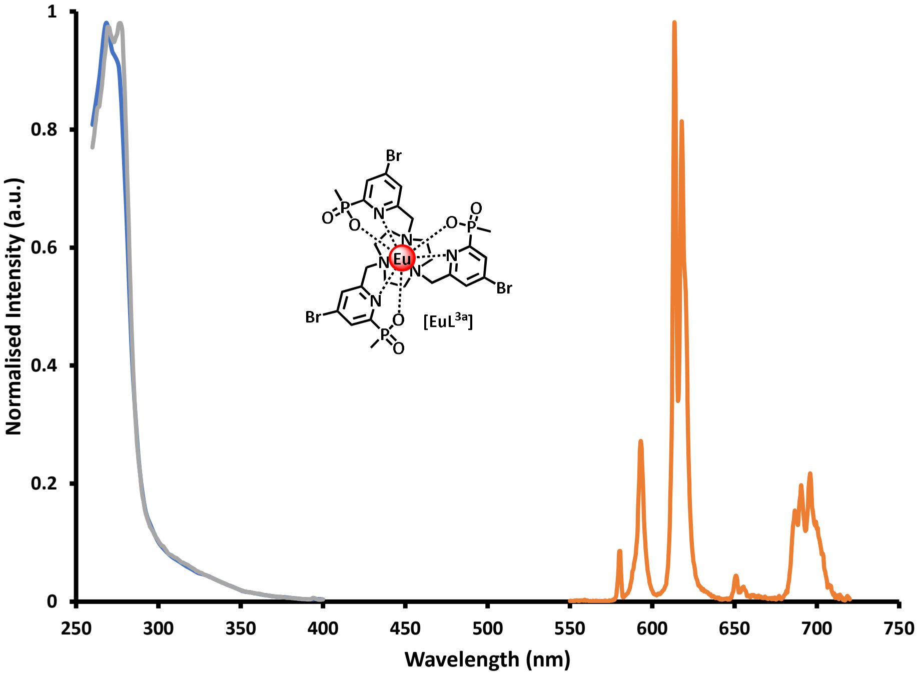

Figure S43. The normalised absorption (*blue*), excitation (*grey*, λ_{em} 615 nm), and emission (*orange*, λ_{exc} 270 nm) spectra, respectively, for [Eu$\boldsymbol{L}^{3a}$] (MeOH, 295 K).

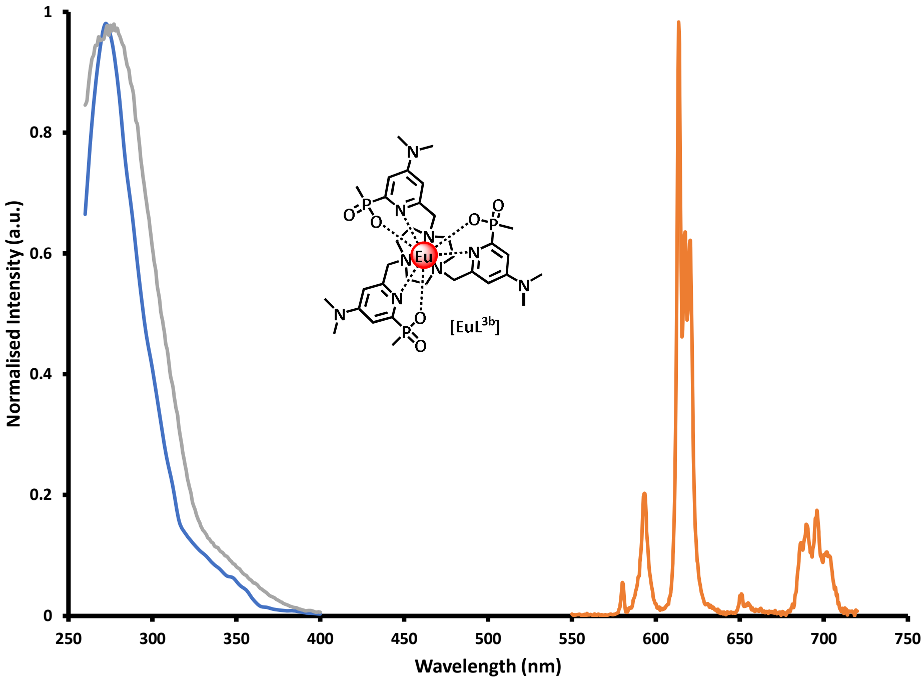

Figure S44. The normalised absorption (*blue*), excitation (*grey*, λ_{em} 615 nm), and emission (*orange*, λ_{exc} 273 nm) spectra, respectively, for [Eu***L***^{3b}] (MeOH, 295 K).

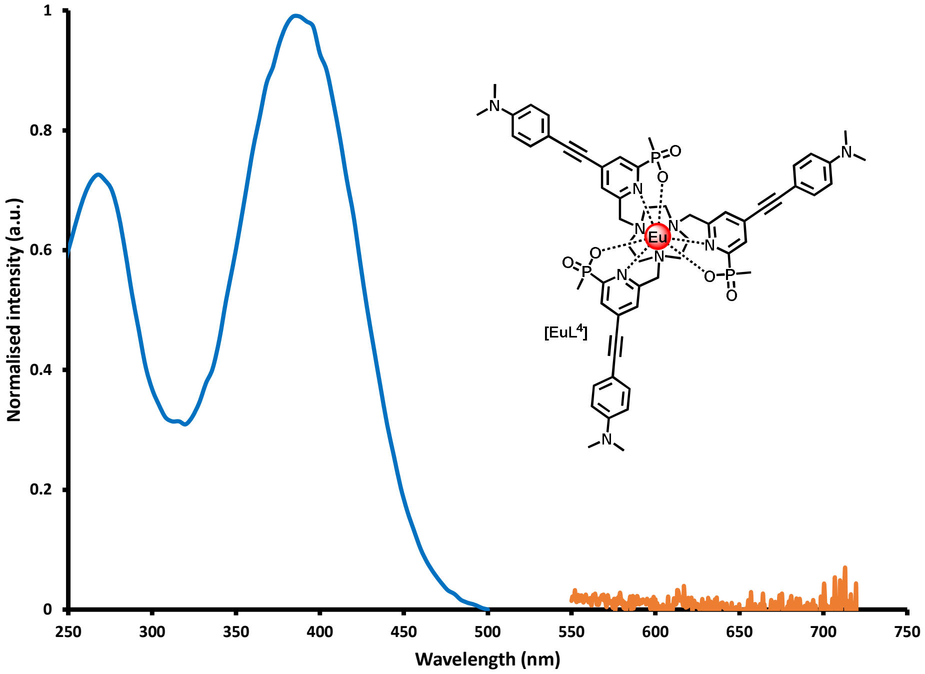

Figure S45. The normalised absorption (*blue*) and emission (*orange*, λ_{exc} 268 nm and 388 nm) spectra for [Eu***L***^{4}] (MeOH, 295 K).

Table S1. Summary of photophysical properties of Eu(III) complexes

	λ_{abs} / nm	ε / M^{-1} cm^{-1}	ϕ_{Eu} / %	τ / ms
**[Eu*L^1a^*]**	280[a], 290[a]	13000[a], 12300[a]	0.6[a]	0.75[a]
**[Eu*L^1b^*]**	290[a], 340[a]	12000[a], 4800[a]	1.3[a]	0.76[a]
**[Eu*L^2a^*]**	284[b]	15000[b]	< 0.05[b]	1.26[b]
**[Eu*L^2b^*]**	276[b]	22000[b]	0.4[b]	0.90[b]
**[Eu*L^2c^*]**	280[b]	19000[b]	1[b]	0.83[b]
**[Eu*L^2d^*]**	282[b]	18000[b]	< 0.05[b]	0.74[b]
**[Eu*L^3a^*]**	270[a], 277[a]	12000[a], 11400[a]	2.7[a]	0.62[a]
**[Eu*L^3b^*]**	273[a]	11000[a]	1.7[a]	0.83[a]
**[Eu*L^5^*]**	268[a], 388[a]	32000[a], 45000[a]	Non emissive	-

a) Data recorded in MeOH at 295 K, b) Data recorded in water at 295 K

X-ray diffraction

X-ray diffraction experiments were carried out on a Bruker 3-circle D8 Venture diffractometer with a PHOTON 100 CMOS area detector, using Mo-K_α radiation (λ=0.71073 Å) from an Incoatec IμS microsource with focussing mirrors. Crystals were cooled using a Cryostream (Oxford Cryosystems) open-flow N_2 gas cryostat. The data were processed using APEX3 v.2016.1-0 and reflection intensities integrated using SAINT v8.38A software (Bruker AXS, 2016). The data were corrected for absorption by numerical integration based on crystal face-indexing, using SADABS program.[8] The structures were solved by dual-space intrinsic phasing method using SHELXT 2018/2 program,[9] and refined by full-matrix least squares using SHELXL 2018/3 software [10] on OLEX2 platform.[11]

Eu(II) complexes characterisation

Chemical Reduction of the Compounds: Each compound (approximately two mg) was dissolved in degassed MeOH (2 mL) under an atmosphere of N_2 in a wet-glovebox (no O_2 but water allowed). To each of the resulting solutions, zinc dust (150 mg) was added, and the mixtures were stirred for 16 h. The resulting solutions were filtered using 20 μm Teflon syringe filters.

UV–visible spectroscopy: UV–visible spectra were acquired using a Shimadzu UV mini-1240 spectrophotometer, and samples were placed into quartz cuvettes that were sealed with paraffin wax under an atmosphere of N_2. Spectra were acquired in methanol at ambient temperature.

Luminescence: Emission spectra were acquired on a Horiba Jobin-Yvon FluoroMax-4 spectrofluorometer. Samples were loaded into quartz cuvettes and sealed with paraffin wax under an atmosphere of N_2. Spectra were acquired with λ_{exc} 350 or 375 nm (Eu$\boldsymbol{L^{1c}}$ only) in methanol at ambient temperature.

EPR spectroscopy: EPR spectra were acquired using a Bruker EMX X-band spectrometer with an Oxford variable-temperature cryostat. EPR samples were prepared in Wilmad Labglass 4mm 707SQ250M tubes to a volume of 300 μL and sealed with paraffin wax under an atmosphere of N_2. Spectra were acquired in methanol at 100 K with a microwave frequency of 9.678654 GHz. Power was modulated for optimal signal.

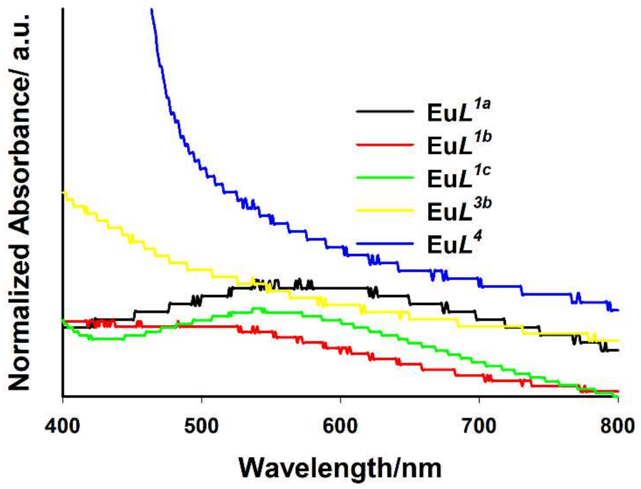

Figure S46. Expansion of the 400 to 800 nm range of UV–visible absorbance spectra of methanol solutions at ambient conditions.

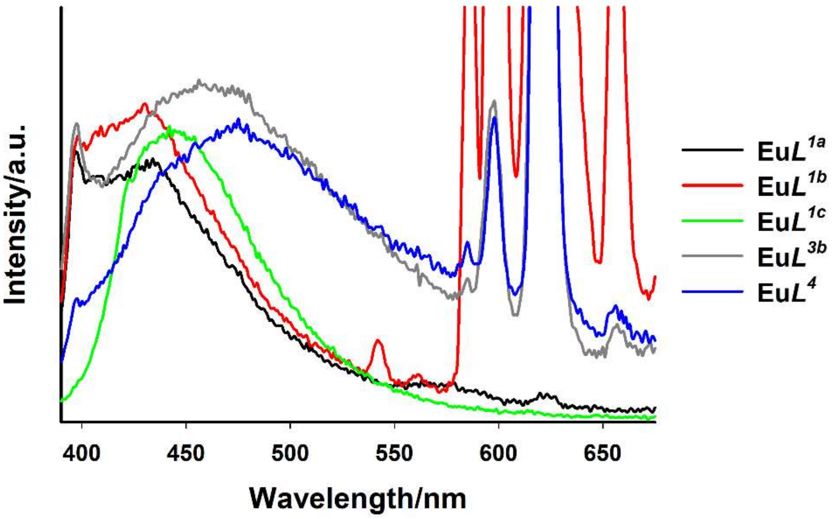

Figure S47. Luminescence spectra of each compound in methanol at ambient temperature. λ_{exc} 350 or 375 nm (Eu***L*^1c^**only).

Electron paramagnetic resonance of Gd(III) complexes

Pulsed EPR measurements were carried on the high-power Q-band (34 GHz) Bruker ELEXSYS E580 with an ER 5106QT-2w cylindrical resonator equipped with a Cryogen-Free Variable Temperature Cryostat (CF-VTC) from Cryogenic Ltd at an operating temperature of 10 K. The solutions, as detailed directly below, were loaded into EPR tubes and flash frozen using liquid nitrogen.

EPR sample preparation: 133 µL of water was added to 0.6 mg of the [GdL^{2c}] complex, but it was found to not dissolve well in water alone. Dissolution was improved with the addition of 133 µL methanol, but the solution was slightly cloudy in appearance so two 50 µL additions of methanol were added to see if this would clear the solution. No changes to the solution's appearance were noted after the additional volume. A benchtop centrifuge was used to remove solid before the EPR sample was made. If all the [GdL^{2c}] had dissolved the concentration would have been 1.81 mM.

It was found both [GdL^{2a}] and [GdL^{2b}] samples were soluble in water but were additionally mixed with methanol to the same ratio as [GdL^{2c}] to keep the conditions reasonably comparable. The mass of each solid was 0.6 mg and 0.8 mg, resulting in final stock concentrations of 1.82 mM and 2.43 mM for the [GdL^{2a}] and [GdL^{2b}] complexes respectively.

Samples were made to be 50 µM for [GdL^{2a}] and [GdL^{2b}] in 100 µL final solution which also contained 50% v/v glycerol. Two separate samples were made from the stock solution. The EPR sample for [GdL^{2c}] was made from the stock solution twice assuming that the stock solution was at 1.81 mM. Comparison of ED-FS signal strengths across all the data estimates that the actual stock concentration was four times less concentrated. A 4 times more concentrated sample was therefore also measured (with consequently more methanol in the final solution) and validated the estimate. All reported data in the SI includes Greek superscripts to denote separate experiments: α-γ are the original measurements and and δ-ζ are repeats of Gd*L* measurements; η is the measurement of the GdL^{2c} complex with four times more of the original stock. The fits of the ED-FS, inversion recovery curves and spin-echo decays are shown for the repeated sets of data for [GdL^{2a}]$^{\delta}$, [GdL^{2b}]$^{\varepsilon}$ and [GdL^{2c}]$^{\zeta}$; parameters are highlighted in results tables with an octothorpe. The results of these fits are the numbers reported in the main paper. However, the results of fitting all the

data are shown in the tables below, and all data sets are freely available at https://doi.org/10.17630/128d8c87-d13a-45d8-a5f2-5d5422b14d1a.

Echo-detected field sweeps: The ED-FS was performed by integrating over a Hahn echo following the $\frac{\pi}{2} - \tau - \pi - \tau - echo$ pulse sequence. The inter-pulse delay (τ) was 200 ns with 16 ns/32 ns $\pi/\frac{\pi}{2}$ pulses lengths respectively and the shot repetition time was 2 ms. The full-width at half-height (FWHH) is found from the ED-FS for each complex and is provided in the tables of results. The ED-FS were collected with 2048 data points which is a relatively course sampling for automated extraction of the FWHH. To combat this, the experimental data points a 1D spline interpolation with 1e09 points was used to provide a more accurate sampling when obtaining the FWHH in MATLAB.

Fits to the experimental ED-FS were performed using the ZFS library toolbox and are given in figures S1-3.[12] The toolbox uses the EasySpin framework to generate a library of simulated EPR spectra which are used in order to fit to the experimental spectra, and for computing the error map from the library of fits and their resultant RMSD when compared to the experimental data.[13] The RMSD is given by the equation (2) where x_i is the scaled simulated data and $\widehat{x_i}$ is the normalised experimental data.

$$\mathrm{RMSD} = \sqrt{\frac{\sum_{i=1}^{N}(x_i - \widehat{x_i})^2}{N}} \qquad (2)$$

The generated library contains a full set of simulations for a given parameter space of *D* and σ*D* for Q-band at 10 K. The parameter space covers a wide range of parameters so that it should include the ZFS parameters of the complex under study, which led to library of values spanning *D* = 300 – 1950, where *E* = *D*/3 in the fitting. The full breakdown of the scripts employed to produce and further fit this library can be found in the supplementary information of the cited paper. The toolbox takes a coarse sampling of the ZFS parameters in 50 MHz steps to obtain the best fit. In order to better estimate the ZFS parameters the minimum RMSD fit is estimated from a fine grid sampling which is interpolated by the fitting of an ellipse to the contour that binds the twice the mean RMSD value (blue asterisk in Fig. S1, S2, and S3 panels b and d). This should be smooth for arbitrary sampling and therefore can be estimated

from the fit (red fitted curve) where the final best fit values can be taken from its centre (red asterisk). A conservative error estimate of these values is taken from the semi-major and semi-minor axes of the ellipse**.** This allowed for a reasonable estimate of the ZFS parameter values from the best fit though it is noted that a multifrequency data set would improve this fitting and confidence. Tables S1 & S2 present the data obtained from these fits and associated errors for the ZFS parameter *D*, and the FWHH of the distribution (strain) *σD*. An area of the central transition is excluded from the fit (green, Fig. S1-3a) since this was seen to improve the quality of the fit to the overall lineshape, and these are the values presented in the main paper (Table 2), but fits including the central transition are also provided.

Relaxation measurements: T_1 relaxation measurements were made using an inversion recovery $\pi - \tau_1 - \frac{\pi}{2} - \tau_2 - \pi - \tau_2 - echo$ sequence. τ_1 was stepped in 1 μs steps after an initial value of 200 ns, τ_2 was fixed at 200 ns. T_m measurements used a $\frac{\pi}{2} - \tau - \pi - \tau - echo$ sequence with τ set initially at 200 ns and incremented by 16 ns. Both experiments were performed at the maximum signal intensity of the ED-FS with a shot repetition time of 4 ms with a 2-step phase cycle.

EPR ED-FS Fits

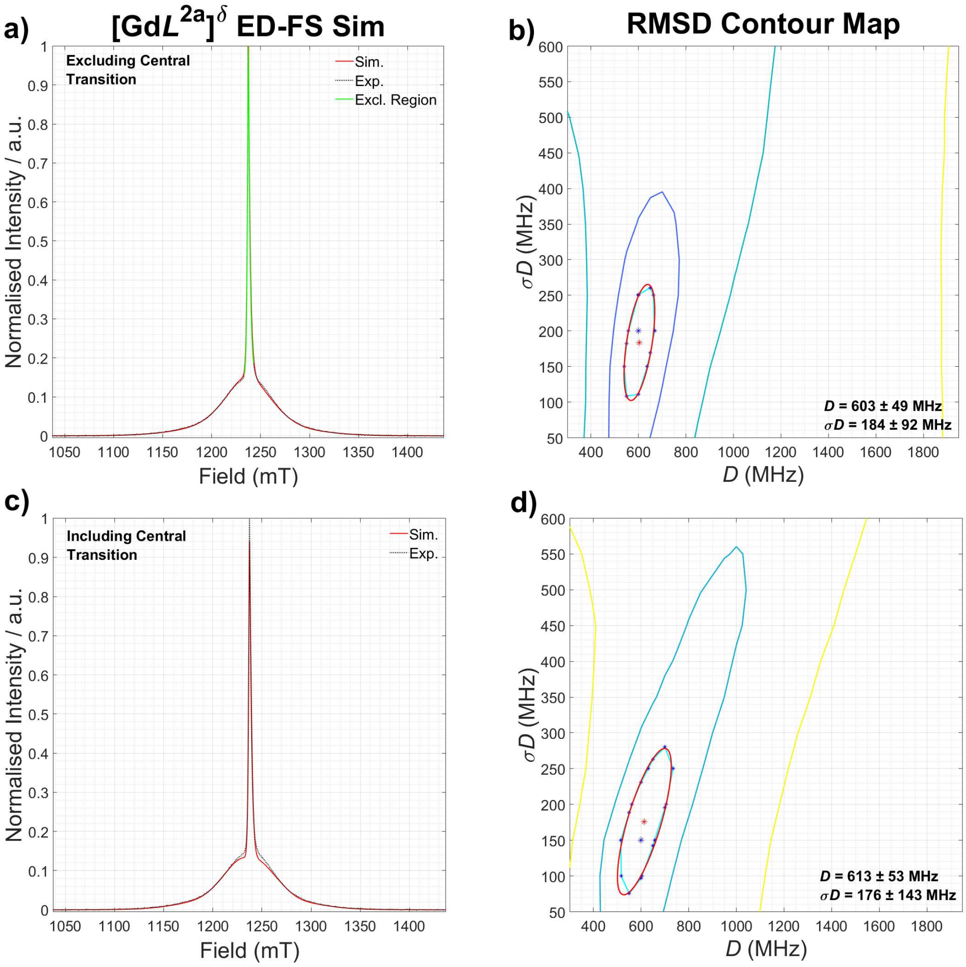

Figure S48. Simulations using the best-fit ZFS parameters for $[Gd\boldsymbol{L}^{2a}]^{\delta}$, without (a) and with (c) the region of the central transition included in the RMSD error map analyses. Contours of constant RMSD as a function of *D* and σ*D* parameter values for the simulated fit without (b) and with (d) the central transition respectively. Each contour line represents a doubling of the minimum RMSD value, the blue asterisk shows the minimum from the set of parameter values on the ZFS library of simulations, and the red asterisk shows the interpolated lowest RMSD value. The full set of parameters for each model is given in Tables S2 and S3.

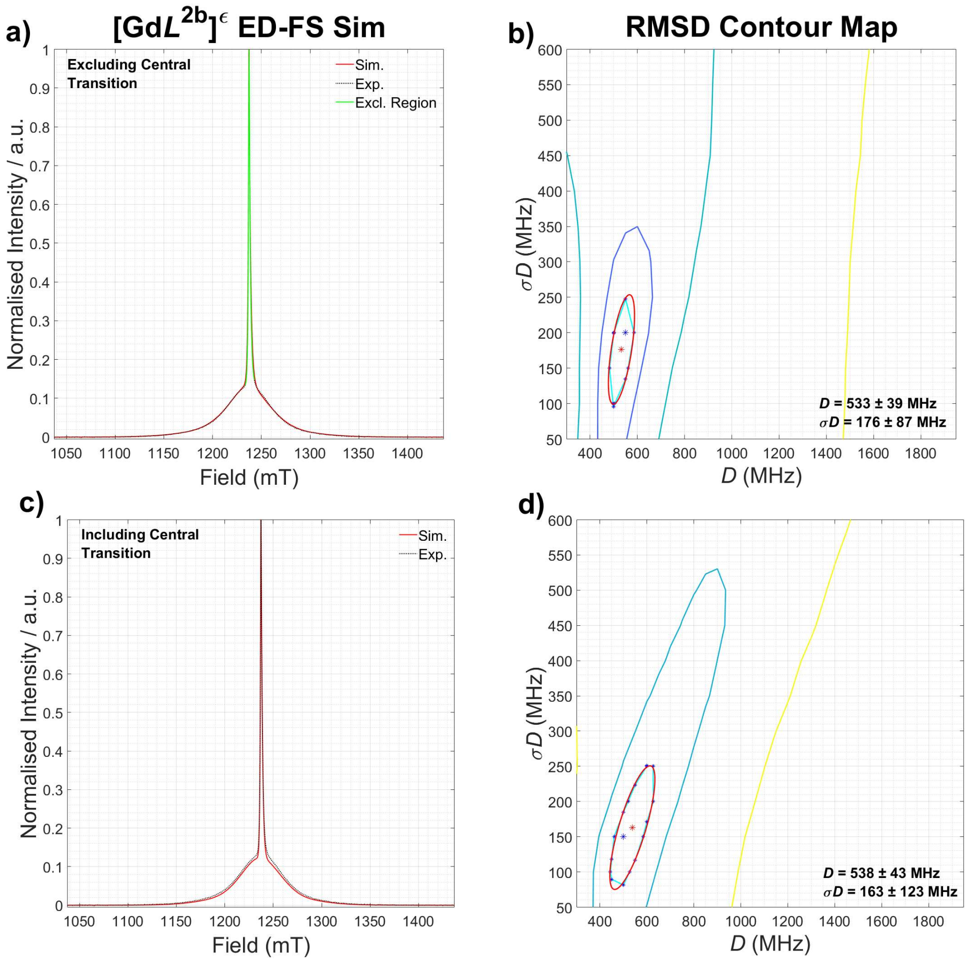

Figure S49. Simulations using the best-fit ZFS parameters for [Gd$\boldsymbol{L}^{2b}$]$^{\epsilon}$, without (a) and with (c) the region of the central transition included in the RMSD error map analyses. Contours of constant RMSD as a function of D and σD parameter values for the simulated fit without (b) and with (d) the central transition respectively. Each contour line represents a doubling of the minimum RMSD value, the blue asterisk shows the minimum from the set of parameter values on the ZFS library of simulations, and the red asterisk shows the interpolated lowest RMSD value. The full set of parameters for each model is given in Tables S2 and S3.

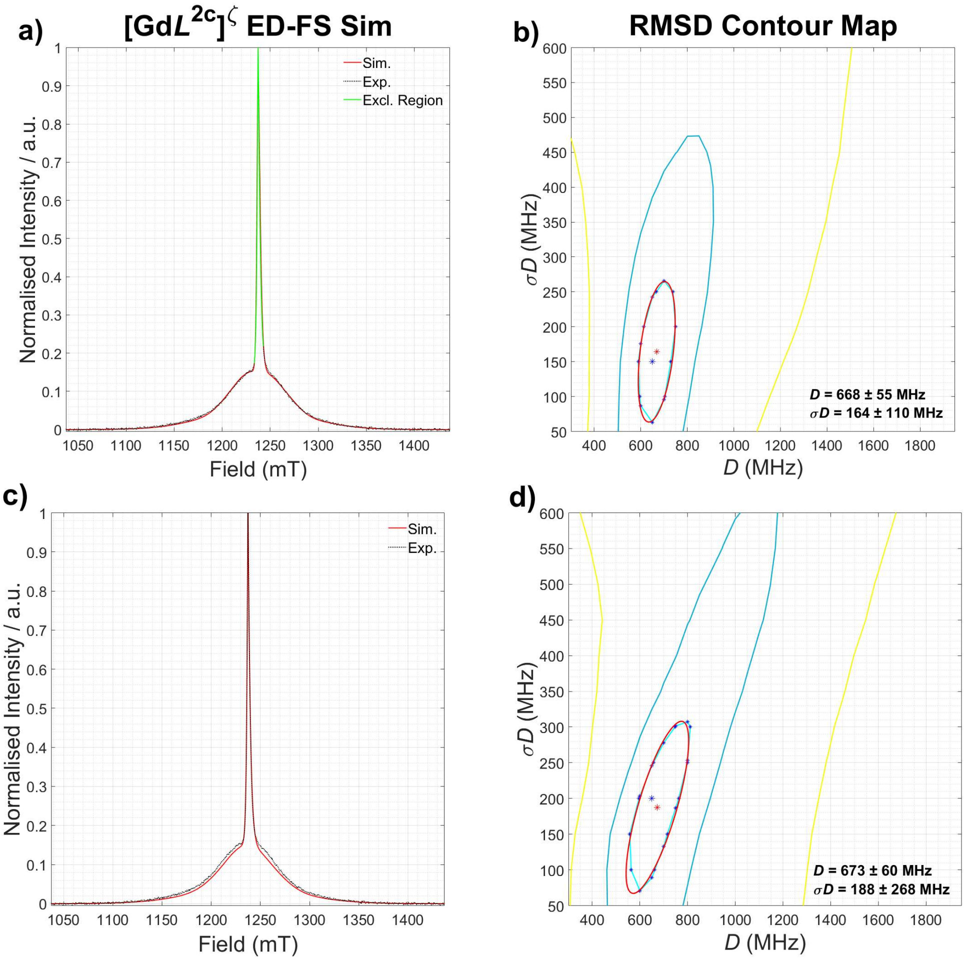

Figure S50. Simulations using the best-fit ZFS parameters for [Gd$\boldsymbol{L^{2c}}$]$^{\zeta}$, without (a) and with (c) the region of the central transition included in the RMSD error map analyses. Contours of constant RMSD as a function of D and σD parameter values for the simulated fit without (b) and with (d) the central transition respectively. Each contour line represents a doubling of the minimum RMSD value, the blue asterisk shows the minimum from the set of parameter values on the ZFS library of simulations, and the red asterisk shows the interpolated lowest RMSD value. The full set of parameters for each model is given in Tables S2 and S3.

Table S2. Parameters found from the analysis of the ED-FS EPR data, excluding the central transition

System	*D*/ MHz [a]	*σD* /MHz [b]	FWHH/ mT [c]
**[Gd*L*^{2a}]^{α}**	636 ± 58	189 ± 108	2.81
**[Gd*L*^{2b}]^{β}**	572 ± 60	193 ± 125	1.95
**[Gd*L*^{2c}]^{γ}**	667 ± 56	167 ± 93	3.36
**[Gd*L*^{2a}]$^{δ\#}$**	603 ± 49	184 ± 92	2.78
**[Gd*L*^{2b}]$^{ε\#}$**	533 ± 39	176 ± 87	1.94
**[Gd*L*^{2c}]$^{ζ\#}$**	668 ± 55	164 ± 110	3.41
**[Gd*L*^{2c}]^{η}**	645 ± 47	175 ± 83	3.28

a) The ZFS parameter *D* found from fits to the experimental data. b) Strain in the ZFS parameter σ*D* from fits to the experimental data. c) FWHH estimated from the central transition line of the ED-FS spectra recorded at 10 K at Q-band. Values are taken straight from the simulated fits, where the central transition was excluded from the fit to improve accuracy. Greek letters are repeated measurements as defined in *EPR Sample Preparation* above, the octothorpe denotes the data sets used for the reported values in the main paper and their fits are shown in Fig. S48-S50.

Table S3. Parameters found from the analysis of the ED-FS EPR data, including the central transition

System	*D*/ MHz [a]	*σD* /MHz [b]	FWHH/ mT [c]
**[Gd*L*^{2a}]^{α}**	642 ± 64	190 ± 200	2.81
**[Gd*L*^{2b}]^{β}**	558 ± 60	175 ± 196	1.95
**[Gd*L*^{2c}]^{γ}**	672 ± 63	186 ± 177	3.36
**[Gd*L*^{2a}]$^{δ\#}$**	613 ± 53	176 ± 143	2.78
**[Gd*L*^{2b}]$^{ε\#}$**	538 ± 43	163 ± 123	1.94
**[Gd*L*^{2c}]$^{ζ\#}$**	673 ± 60	188 ± 268	3.41
**[Gd*L*^{2c}]^{η}**	625 ± 24	162 ± 78	3.28

a) The ZFS parameter *D* found from fits to the experimental data. b) Strain in the ZFS parameter σ*D* from fits to the experimental data. c) FWHH estimated from the central transition line of the ED-FS spectra recorded at 10 K at Q-band. Values are taken straight from the simulated fits, where the central transition was included in the fit. Greek letters are repeated measurements as defined in *EPR Sample Preparation* above, the octothorpe denotes the data sets used for the reported values in the main paper where the values are taken from Table S3, and their fits are shown in Fig. S48-S50.

EPR Relaxation Fits

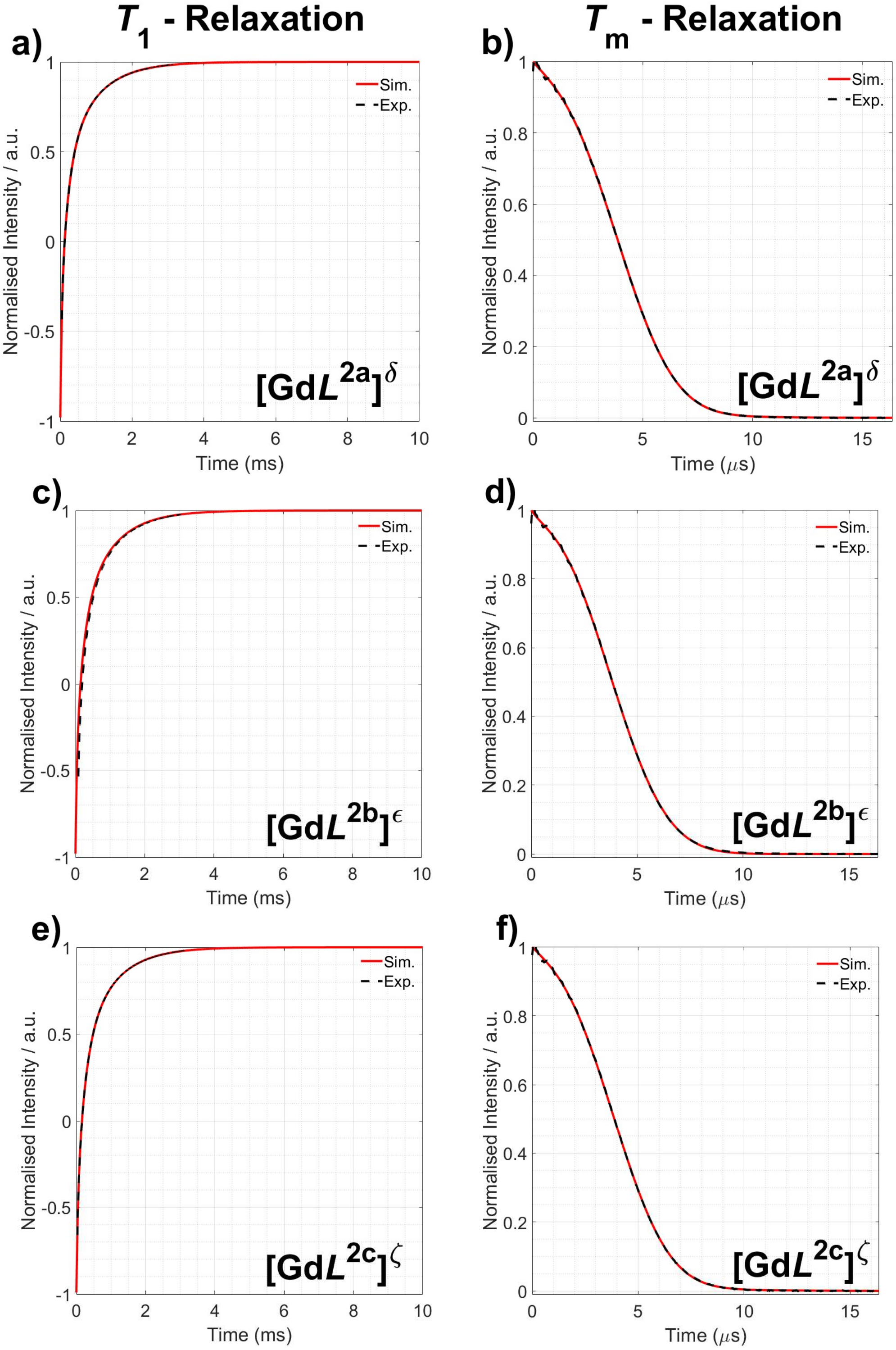

Figure S51. Inversion recovery (to measure T_1 relaxation) and echo-decay (to measure T_m relaxation) data for Gd*L*. The experimental data is shown in black dash, with the fits shown in red and the data are normalised to the maximum of the fits. All parameters for the fits are given in the notes for **Tables S4 and S5**.

Table S4. T_1 times, and associated parameters, derived from the fit of the inversion-recovery experimental data shown in Figure S51.

System	T_1^a / ms	A / %	T_1^b / ms	B / %	$T_1^{\#}$ / ms
**[Gd*L*^{2a}]$^{\alpha}$**	0.032 ± (0.001)	40.1 (± 0.4)	0.476 (± 0.004)	59.9 (±0.4)	0.253
**[Gd*L*^{2b}]$^{\beta}$**	0.027 ± (0.001)	37.8 (± 0.4)	0.503 (± 0.004)	62.1 (± 0.3)	0.306
**[Gd*L*^{2c}]$^{\gamma}$**	0.035 ± (0.001)	38.6 (± 0.4)	0.521 (± 0.005)	61.4 (± 0.4)	0.289
**[Gd*L*^{2a}]$^{\delta\#}$**	0.010 ± (0.001)	43.2 (± 0.4)	0.445 (± 0.004)	56.8 (±0.4)	0.226
**[Gd*L*^{2b}]$^{\varepsilon\#}$**	0.013 ± (0.001)	41.3 (± 0.4)	0.493 (± 0.004)	58.7 (± 0.3)	0.270
**[Gd*L*^{2c}]$^{\zeta\#}$**	0.030 (0.001)	38.6 (± 0.4)	0.508 (± 0.005)	61.4 (± 0.4)	0.287
**[Gd*L*^{2c}]$^{\eta}$**	0.024 ± (0.001)	39.7 (± 0.4)	0.472 (± 0.005)	60.3 (± 0.4)	0.256

The data were fit with a sum of two exponential functions:

$$A * \left(1 - 2 * exp\left(-\frac{t}{T_1^a}\right)\right) + B * \left(1 - 2 * exp\left(-\frac{t}{T_1^b}\right)\right). \quad (3)$$

Values for A, B and T_1^a and T_1^b for this fit are given in the table, A and B are given in % of the total (sum of A and B) amplitude. $T_1^{\#}$ values were derived by assuming a single exponential Bloch model for relaxation where the intensity value at 63% recovery is reported (corresponding to the x value when y = 0.26 for the curves normalised to their maximum), this is extracted from the full interpolated simulation when normalised to 1 since the experimental data has not fully recovered. This is the value for T_1 reported in the main paper's Table 2. All values were obtained using the custom function with the Curve Fitting Toolbox within MATLAB R2020b. Greek letters are repeated measurements as defined in EPR Sample Preparation above, the octothorpe denotes the data sets used for the reported values in the main paper.

Table S5. T_m times, and associated parameters, derived from the fit of the echo-decay experimental data shown in Figure S51.

**System**	T_m^a **(μs)**	***A* / %**	T_m^b **/ μs**	***B* / %**	$T_m^{\#}$**, / μs**
**[Gd*L*^{2a}]$^{\alpha}$**	0.14 (+/- 0.05)	0.7 (+/- 0.1)	4.42 (+/- 0.01)	99.3 (+/- 0.1)	6.432
**[Gd*L*^{2b}]$^{\beta}$**	0.02 (+/- 0.01)	1.2 (+/- 0.3)	4.41 (+/- 0.01)	98.8 (+/- 0.1)	6.528
**[Gd*L*^{2c}]$^{\gamma}$**	0.01 (+/- 0.01)	0.01 (+/- 0.01)	4.32 (+/- 0.01)	99.9 (+/- 0.1)	6.528
**[Gd*L*^{2a}]$^{\delta\#}$**	0.04 (+/- 0.05)	1.7 (+/- 0.1)	4.51 (+/- 0.01)	98.3 (+/- 0.1)	6.528
**[Gd*L*^{2b}]$^{\varepsilon\#}$**	0.02 (+/- 0.01)	1.4 (+/- 0.3)	4.49 (+/- 0.01)	98.6 (+/- 0.1)	6.496
**[Gd*L*^{2c}]$^{\zeta\#}$**	0.01 (+/- 0.01)	0.03 (+/- 0.01)	4.51 (+/- 0.01)	99.7 (+/- 0.1)	6.544
**[Gd*L*^{2c}]$^{\eta}$**	0.02 (+/- 0.01)	0.05 (+/- 0.01)	4.49 (+/- 0.01)	99.5 (+/- 0.1)	6.446

The data were fitted by the sum of two exponential functions with stretching exponents of 1 and 2:

$$A * \exp\left(-\frac{t}{T_m^a}\right)^1 + B * \exp\left(-\frac{t}{T_m^b}\right)^2. \quad (4)$$

The resultant values for *A*, *B*, T_m^a and T_m^b are shown in the table. *A* and *B* are given in % of the total (sum of *A* and *B*) amplitude. $T_m^{\#}$, is the time taken (from the π/2 pulse) for the echo intensity to reach 10% of its initial value. This is the value for T_m reported in the main paper's Table 2. All values were obtained using the custom functions with the Curve Fitting Toolbox within MATLAB R2020b. Greek letters are repeated measurements as defined in EPR Sample Preparation above, the octothorpe denotes the data sets used for the reported values in the main paper.

References

[1] H. Takalo, I. Hemmilä, T. Sutela, M. Latva, *Helv. Chim. Acta* **1996**, *79*, 789-802.

[2] N. H. Evans, R. Carr, M. Delbianco, R. Pal, D. S. Yufit, D. Parker, *Dalton Trans.* **2013**, *42*, 15610-15616.

[3] T. Kálai, M. R. Fleissner, J. Jekő, W. L. Hubbell, K. Hideg, *Tetrahedron Lett.* **2011**, *52*, 2747-2749.

[4] E. R. Neil, A. M. Funk, D. S. Yufit, D. Parker, *Dalton Trans.* **2014**, *43*, 5490-5504.

[5] A. Shah, A. Roux, M. Starck, J. A. Mosely, M. Stevens, D. G. Norman, R. I. Hunter, H. E. Mkami, G. M. Smith, D. Parker, J. E. Lovett, *Inorg. Chem.* **2019**, *58*, 3015-3025.

[6] Y. Haas, G. Stein, *J. Phys. Chem.* **1971**, *75*, 3668-3677.

[7] L. J. Charbonnière, R. Schurhammer, S. Mameri, G. Wipff, R. F. Ziessel, *Inorg. Chem.* **2005**, *44*, 7151-7160.

[8] L. Krause, R. Herbst-Irmer, G. M. Sheldrick, D. Stalke, *J. Appl. Crystallogr.* **2015**, *48*, 3-10.

[9] G. M. Sheldrick, *Acta Crystallogr. A* **2015**, *71*, 3-8.

[10] G. M. Sheldrick, *Acta Crystallogr. Sect. C: Cryst. Struct. Commun.* **2015**, *71*, 3-8.

[11] O. V. Dolomanov, L. J. Bourhis, R. J. Gildea, J. A. K. Howard, H. Puschmann, *J. Appl. Crystallogr.* **2009**, *42*, 339-341.

[12] J. A. Clayton, K. Keller, M. Qi, J. Wegner, V. Koch, H. Hintz, A. Godt, S. Han, G. Jeschke, M. S. Sherwin, M. Yulikov, *Phys. Chem. Chem. Phys.* **2018**, *20*, 10470-10492.

[13] S. Stoll, A. Schweiger, *J. Magn. Reson.* **2006**, *178*, 42-55.
